# Smoothened transduces Hedgehog signals via activity-dependent sequestration of PKA catalytic subunits

**DOI:** 10.1371/journal.pbio.3001191

**Published:** 2021-04-22

**Authors:** Corvin D. Arveseth, John T. Happ, Danielle S. Hedeen, Ju-Fen Zhu, Jacob L. Capener, Dana Klatt Shaw, Ishan Deshpande, Jiahao Liang, Jiewei Xu, Sara L. Stubben, Isaac B. Nelson, Madison F. Walker, Kouki Kawakami, Asuka Inoue, Nevan J. Krogan, David J. Grunwald, Ruth Hüttenhain, Aashish Manglik, Benjamin R. Myers

**Affiliations:** 1 Department of Oncological Sciences, Department of Biochemistry, Department of Bioengineering, University of Utah School of Medicine, Salt Lake City, Utah, United States of America; 2 Department of Human Genetics, University of Utah School of Medicine, Salt Lake City, Utah, United States of America; 3 Department of Pharmaceutical Chemistry, Department of Anaesthesia and Perioperative Care, University of California, San Francisco, California, United States of America; 4 Department of Cellular and Molecular Pharmacology, Quantitative Biosciences Institute, University of California, San Francisco, California, United States of America; 5 J. David Gladstone Institutes, San Francisco, California, United States of America; 6 Graduate School of Pharmaceutical Sciences, Tohoku University, Sendai, Miyagi, Japan; University of Zurich, SWITZERLAND

## Abstract

The Hedgehog (Hh) pathway is essential for organ development, homeostasis, and regeneration. Dysfunction of this cascade drives several cancers. To control expression of pathway target genes, the G protein–coupled receptor (GPCR) Smoothened (SMO) activates glioma-associated (GLI) transcription factors via an unknown mechanism. Here, we show that, rather than conforming to traditional GPCR signaling paradigms, SMO activates GLI by binding and sequestering protein kinase A (PKA) catalytic subunits at the membrane. This sequestration, triggered by GPCR kinase (GRK)-mediated phosphorylation of SMO intracellular domains, prevents PKA from phosphorylating soluble substrates, releasing GLI from PKA-mediated inhibition. Our work provides a mechanism directly linking Hh signal transduction at the membrane to GLI transcription in the nucleus. This process is more fundamentally similar between species than prevailing hypotheses suggest. The mechanism described here may apply broadly to other GPCR- and PKA-containing cascades in diverse areas of biology.

## Abstract 2

This study shows that Hedgehog signal transduction during embryonic development and in cancer employs an unusual biochemical mechanism involving sequestration of protein kinase A at the membrane, mediated by the GPCR Smoothened.

## Introduction

The Hedgehog (Hh) pathway controls the development of nearly every vertebrate organ [1–4]. It also plays critical roles in stem cell biology and injury-induced tissue regeneration [5,6]. Insufficient pathway activation during embryogenesis gives rise to birth defects [7], whereas ectopic pathway activity drives several malignancies, including basal cell carcinoma of the skin and pediatric medulloblastoma [8,9].

Hh signal reception at the membrane is tightly coupled to transcriptional regulation of pathway target genes in the nucleus [1,4,10,11]. In the pathway “off” state, Patched1 (PTCH1) inhibits the G protein–coupled receptor (GPCR) Smoothened (SMO). In the pathway “on” state, Hh proteins bind to and inactivate PTCH1, relieving SMO from inhibition [1,4,10,11]. SMO activation ultimately results in the conversion of glioma-associated (GLI) transcription factors from repressor to activator forms [1,4,11]. Active GLI regulates expression of Hh pathway target genes that drive cell differentiation or proliferation [12]. The process by which vertebrate SMO activates GLI, however, is largely a mystery.

An appealing model suggests that SMO activates GLI by blocking protein kinase A (PKA), thereby releasing GLI from PKA-mediated inhibition [4,13–15]. In support of this model, inactivation of PKA catalytic subunits (PKA-C) induces the Hh pathway to near-maximal levels [16–20]. In addition, PKA phosphorylation of GLI hinders its transcriptional activity, while SMO activation results in loss of phosphorylation at these sites [21–25]. Furthermore, SMO, PKA, and GLI may communicate directly with one another within a cell surface organelle known as the primary cilium, as all 3 proteins localize in or near this subcellular compartment [20,26–31]. Nevertheless, the above model is controversial, in part, because the role of G proteins, which canonically link GPCR activation to PKA inhibition, remains a matter of debate in SMO–GLI communication. While some studies point to roles for inhibitory (Gα_i/o/z_) G proteins in SMO activation of GLI [32,33], others have concluded that inhibitory G proteins are not absolutely required for this process [34–36]. Thus, although PKA has been implicated in communication between SMO and GLI, the molecular mechanism by which SMO activates GLI remains poorly understood [1,4,11,14].

To dissect SMO–GLI communication, we used a heterologous cellular system to identify and reconstitute the Hh pathway step immediately downstream of SMO. We then characterized the underlying biochemical mechanism and assessed the physiological relevance of our findings using established cellular and embryological assays of Hh signal transduction. Using this approach, we found that activated SMO blocks PKA substrate phosphorylation by directly binding and sequestering PKA-C subunits at the membrane. This prevents PKA phosphorylation of GLI, thereby triggering GLI activation. PKA-C binding to SMO is controlled by GRK family kinases that selectively phosphorylate the SMO active conformation on conserved residues in the intracellular domain. Our work reveals an unconventional route by which GPCRs can control PKA activity—one that may also be utilized by other signaling pathways that employ these proteins.

## Results

### SMO inhibits PKA substrate phosphorylation in a G protein–independent manner

We hypothesized that SMO can inhibit PKA via a G protein–independent process. To test this hypothesis, we set up a model system to study SMO regulation of PKA. GLI-based readouts are problematic in this regard, as they are affected by manipulation of either SMO or PKA; this makes it difficult to determine whether SMO and PKA reside in the same linear pathway or constitute 2 separate influences that converge on GLI. To overcome this and other confounding factors (see “Methods”), we reconstituted SMO regulation of PKA in a HEK293 model system using a non-GLI readout of PKA activity. This approach also allows us to employ CRISPR, biochemical, and fluorescence-based tools that are uniquely robust in HEK293 cells.

**Fig 1:**
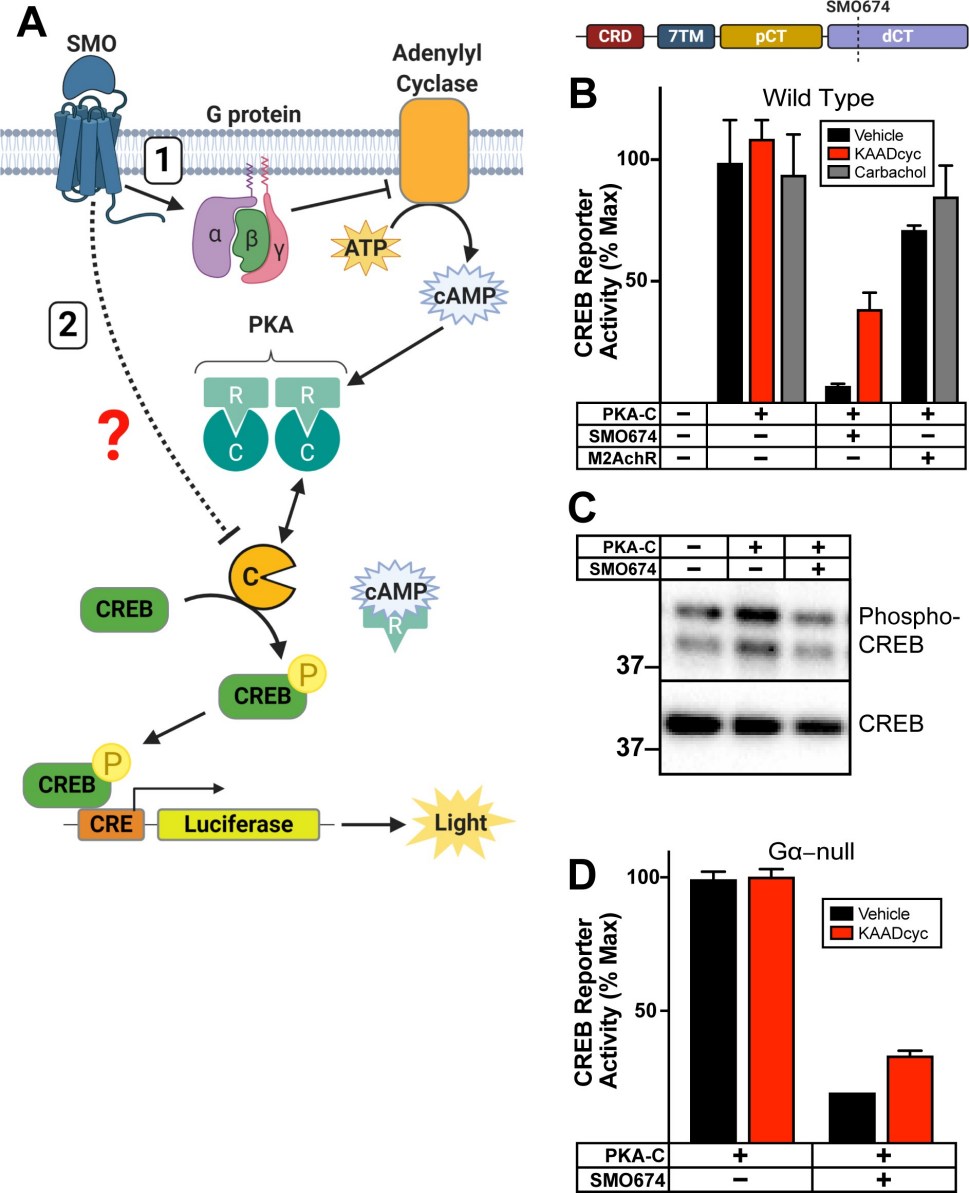
SMO inhibits PKA substrate phosphorylation in a G protein–independent manner. (**A**) Schematic of assay to report PKA activity toward soluble substrates. PKA-C phosphorylates CREB which binds CRE, inducing expression of luciferase. SMO can inhibit PKA-C by decreasing cAMP via inhibitory G proteins and AC (route “1”). Alternatively, SMO may inhibit PKA-C via a G protein–independent mechanism (route “2”). (**B**) Wild-type HEK293 cells were transfected with CRE-luciferase reporter plasmid and GFP (as a negative control) or PKA-C, either alone, with SMO674 (see cartoon above), or with a canonical Gα_i/o_-coupled GPCR, M2AchR. Transfected cells were treated with the indicated drugs (vehicle control, M2AchR ligand carbachol (3 μM), or SMO inverse agonist KAADcyc (1 μM)). Following drug treatment, cells were lysed and luminescence measured. Note that transfected SMO is constitutively active in HEK293 cells because its inhibitor PTCH1 is present at minimal levels [32,33,41,42], whereas M2AchR requires carbachol for activity. For the sake of clarity, the SMO constructs utilized in each experiment are indicated in the corresponding figure panel. (See **S1B Fig** for additional information.) (**C**) Wild-type HEK293 cells were transfected with GFP (as a negative control) or PKA-C, either alone or with SMO674. Lysates were separated via SDS-PAGE and probed with antibodies against phospho-CREB (top) or total CREB (bottom). Molecular masses are in kDa. (**D**) HEK293 Gα-null cells were transfected with PKA-C, either alone or with SMO674, and treated with vehicle or KAADcyc (1 μM). All CREB reporter data are normalized to 100%, which reflects reporter activation from PKA-C-transfected cells treated with vehicle (*n* = 3 biological replicates per condition; error bars = SEM). The underlying data for this figure can be found under **S1 Data**. The uncropped westerns are included in **S8 Data**. See **S1 Table** for statistical analysis. AC, adenylyl cyclase; ATP, adenosine triphosphate; cAMP, cyclic AMP; CRE, cyclic AMP response element; CREB, cyclic AMP response element binding protein; dCT, distal segment of the cytoplasmic tail; GPCR, G protein–coupled receptor; KAADcyc, KAAD-cyclopamine; M2AchR, M2 acetylcholine receptor; pCT, proximal segment of the cytoplasmic tail; PKA, protein kinase A; PKA-C, PKA catalytic subunits; SMO, Smoothened.

To report PKA activity, we utilized cyclic AMP response element (CRE) binding protein (CREB) transcription factors (**Fig 1A**). CREB is activated by PKA phosphorylation [37], but is not known to be subject to the other major mechanisms that regulate GLI activity [12,37]. Our studies employed carboxyl-terminally truncated versions of SMO (either SMO657 or SMO674; **S1A and S1B Fig**). These truncations contain the proximal segment of the cytoplasmic tail (pCT) that is essential for GLI activation but lack the nonessential distal segment of the cytoplasmic tail (dCT) (**S1A and S1C Fig**) [38,39]. Removing the dCT improves SMO expression levels and detergent solubility (**S1D Fig**), thereby facilitating our subsequent biochemical analyses.

In its active state, SMO, like many GPCRs, can block PKA-C by engaging inhibitory G proteins (Gα_i/o/z_) that inactivate adenylyl cyclase (AC), decrease cyclic AMP (cAMP), and promote PKA-C binding to regulatory (PKA-R) subunits to form an inactive holoenzyme (**Fig 1A**, route “1”). We hypothesized, however, that SMO may directly inhibit free PKA-C subunits via a G protein–independent mechanism (**Fig 1A**, route “2”). In this case, active SMO, but not other Gα_i/o/z_-coupled GPCRs, would block CREB reporter activation mediated by G protein–independent pathways.

To test this hypothesis, we expressed exogenous PKA-C, at levels likely to exceed those of endogenous PKA-R, to bypass G protein–dependent cascades. As expected, PKA-C expression strongly activated the CREB reporter (**Fig 1B**), indicating an excess of PKA-C over PKA-R. Cotransfection of SMO blocked PKA-C-mediated reporter activation (**Fig 1B**), indicating that SMO can inhibit PKA-C in a G protein–independent manner. (Note that SMO is constitutively active in HEK293 cells, as its inhibitor PTCH1 is expressed at minimal levels [32,33,40–42]). This blockade was partially reversed by coexpression of PTCH1 (**S1E Fig**), or by the specific SMO inverse agonist KAAD-cyclopamine (KAADcyc) (**Fig 1B**), indicating a dependence on SMO activity. The partial effects of KAADcyc in this assay may stem from the fact that CREB reporter activity is not a 1:1 readout of SMO conformational state and may not be linearly proportional to SMO activity over the entire range of SMO activity levels. Consistent with this hypothesis, KAADcyc more fully reversed effects of SMO in experiments where SMO blocked the reporter submaximally (**S1F Fig**). In contrast to SMO, activation of a canonical Gα_i/o_-coupled GPCR, the M2 acetylcholine receptor (M2AchR), with its ligand carbachol did not block the effects of PKA-C expression (**Fig 1B**). This result cannot be explained by issues with receptor expression, trafficking, or ligand stimulation, because carbachol treatment of M2AchR-expressing cells readily blocked AC-evoked reporter activation (**S1G Fig**). We also observed that SMO can attenuate PKA-C–induced phosphorylation of CREB in HEK293 cells, using immunoblotting with CREB and phospho-CREB antibodies (**Fig 1C**). These experiments indicate that SMO can regulate PKA-C in a G protein–independent manner.

We verified this conclusion by showing that SMO was also able to block PKA-C in HEK293 Gα-null cells harboring CRISPR-mediated deletions in all 13 human Gα genes [43] (**Fig 1D**). In contrast, M2AchR failed to inhibit PKA-C (**S1G Fig**), consistent with its function as a canonical Gα_i/o_-coupled GPCR. As in wild-type HEK293 cells, KAADcyc more fully reversed effects of SMO in experiments where SMO blocked PKA-C submaximally (**S1F Fig**). Taken together, our findings indicate that SMO can inhibit PKA substrate phosphorylation even when G proteins are absent.

### SMO uses its essential pCT domain to recruit PKA-C to the membrane

GPCRs control PKA in part via G protein–and cAMP-dependent regulation of its enzymatic activity [44,45]. However, another critical determinant of substrate phosphorylation is PKA’s localization within the cell. Interactions of PKA with specific receptors, signaling scaffolds, and anchoring proteins can bias its enzymatic activity toward certain subcellular locations and away from others [46,47]. We hypothesized that SMO might control PKA subcellular localization, thereby restricting access of PKA to soluble substrates. Such a model could explain how SMO blocks CREB phosphorylation without strictly requiring G proteins (**Fig 1**).

**Fig 2:**
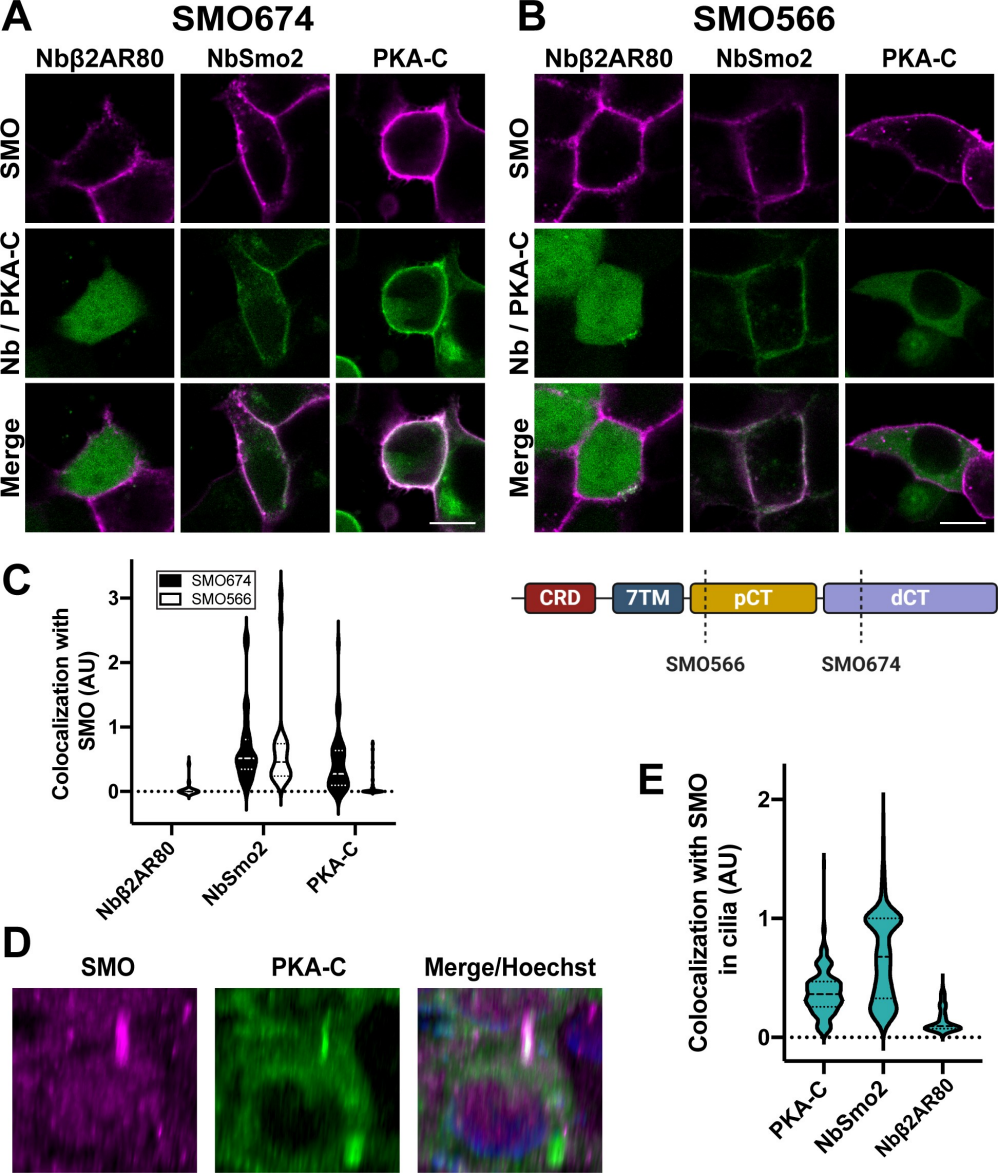
SMO uses its essential pCT domain to recruit PKA-C to the membrane. HEK293 cells expressing (**A**) FLAG-tagged SMO674 that contains the pCT or (**B**) FLAG-tagged SMO566 that lacks the pCT (see cartoon at lower right) were cotransfected with Nbβ2AR80-GFP, NbSmo2-YFP, or PKA-C-YFP. Confocal microscopy images show SMO (magenta) and co-expressed proteins (green). Scale bar = 10 μm. (**C**) Quantification of colocalization for studies in (A) and (B). (*n* = 29–121 cells per condition). Note that Nbβ2AR80 displayed a background-subtracted colocalization index of “0” in all SMO674-expressing cells examined. (**D**) Three-dimensional reconstruction of IMCD3 cells stably coexpressing FLAG-tagged SMO (magenta) with mNeonGreen-tagged PKA-C (green). After treatment with SAG21k to induce SMO accumulation in cilia, live cells were stained with anti-FLAG antibodies (magenta) and Hoechst (blue), then examined via confocal microscopy. See **S3 Fig** for images from cells expressing FLAG-tagged SMO with mNeonGreen-tagged NbSmo2 or Nbβ2AR80. (**E**) Quantification of colocalization between SMO and PKA-C, NbSmo2, or Nbβ2AR80 for the experiment described in (D) (*n* = 197–236 cilia per condition). The underlying data for this figure can be found under **S2 Data**. dCT, distal segment of the cytoplasmic tail; pCT, proximal segment of the cytoplasmic tail; PKA-C, PKA catalytic subunits; SMO, Smoothened.

We tested this hypothesis by examining the effect of SMO on the subcellular localization of PKA-C in HEK293 cells. In the presence of SMO, PKA-C localized to the membrane, colocalizing with SMO in a majority of cells (**Fig 2A and 2C**). In contrast, PKA-C did not display membrane localization when SMO was absent (**S2A Fig**). As a positive control, we examined SMO colocalization with nanobody (Nb) NbSmo2, which binds efficiently and specifically to the activated conformation of SMO (**Figs 2A and S2B and S2C**) via an intracellular epitope within the seven-transmembrane (7TM) domain (**S2C–S2E Fig**). As a negative control, the non-SMO-binding Nb, Nbβ2AR80 [48,49], did not colocalize with SMO (**Figs 2A and S3A**). Deletion of the SMO pCT abolished colocalization with PKA-C, but not with NbSmo2 (**Figs 2B and 2C and S3A**). Control experiments confirmed that SMO674 and SMO566 were present at similar levels on the cell surface (**S3B Fig**). Thus, the SMO pCT, which is essential for activation of GLI (**S1C Fig**), is also required to sequester PKA-C at the membrane.

We next tested whether our findings from imaging experiments in HEK293 cells hold true in primary cilia, the endogenous subcellular location for Hh signal transduction. To this end, we examined SMO and PKA-C localization in inner medullary collecting duct (IMCD3) cells, a robustly ciliated cell line that is widely utilized in studies of Hh signal transduction in primary cilia [50–53]. Consistent with our HEK293 studies, FLAG-tagged SMO and mNeonGreen-tagged PKA-C colocalized in primary cilia of live IMCD3 cells (**Fig 2D and 2E**). mNeonGreen-tagged NbSmo2 and Nbβ2AR80 served as positive and negative controls, respectively (**S3C Fig**). We conclude that SMO/PKA-C colocalization is not limited to HEK293 plasma membranes, but can also occur in a ciliary context.

### The SMO pCT interacts with PKA-C

**Fig 3:**
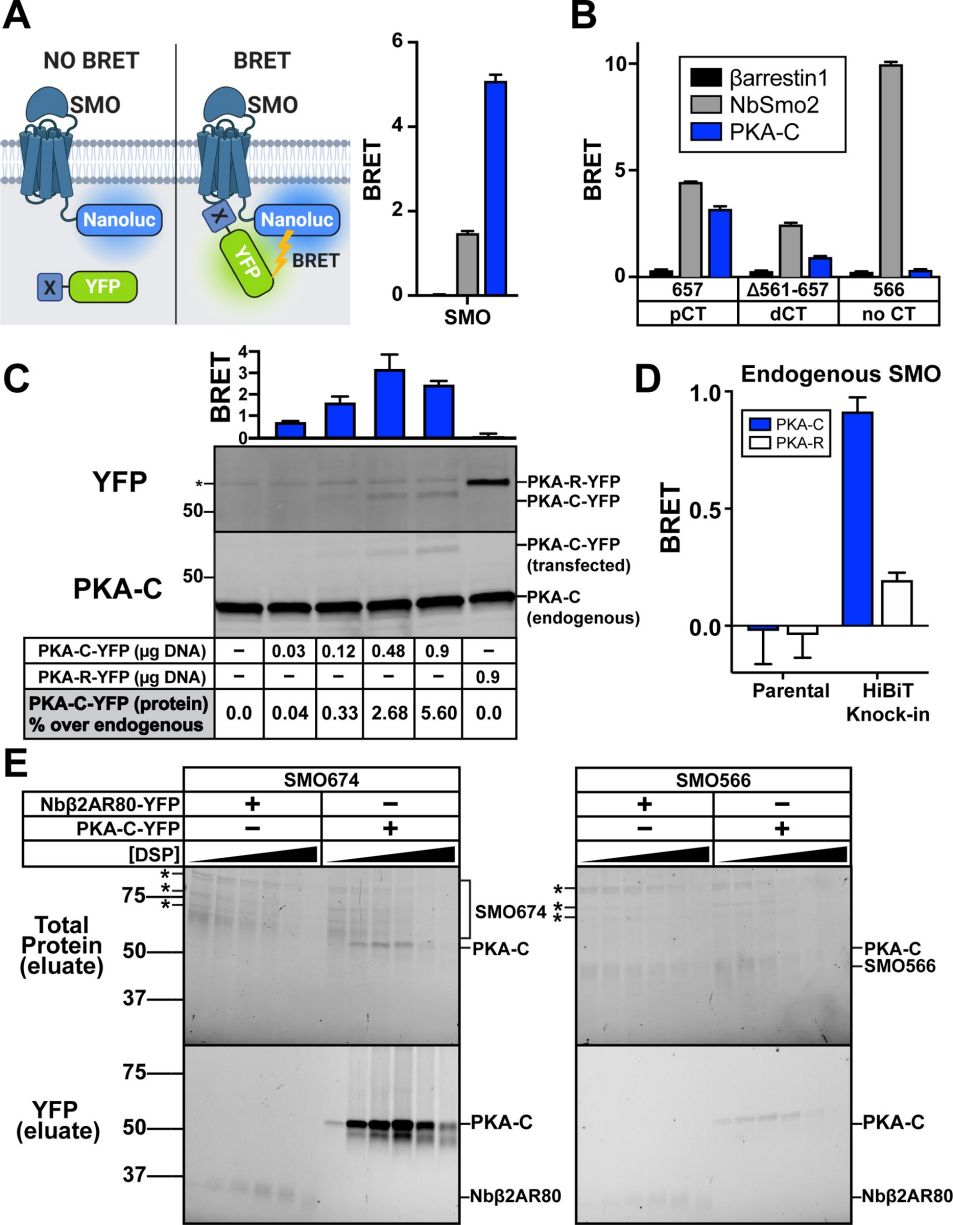
The SMO pCT interacts with PKA-C. (**A**) (Left) Schematic showing BRET between a nanoluc-tagged donor (SMO-nanoluc) and a YFP-tagged acceptor. (Right) HEK293 cells were transfected with nanoluc-tagged full-length SMO, along with YFP-tagged βarrestin1 (black), NbSmo2 (gray), or PKA-C (blue) and subject to BRET analysis. (**B**) HEK293 cells were transfected with nanoluc-tagged SMO657 (which contains the pCT), SMOΔ561–657 (which contains the dCT), or SMO566 (which lacks the CT entirely) as donors, along with the indicated acceptors. Color codes are the same as in (A). Note that NbSmo2 binding does not require the SMO CT (see **S2 Fig**). In fact, NbSmo2 BRET increases upon SMO CT truncation, likely because the decreased distance between the NbSmo2 binding site and the nanoluc tag leads to more efficient BRET. (**C**) Top: IMCD3 cells were transfected with nanoluc-tagged full-length SMO along with the indicated amounts of PKA-C-YFP plasmid (in μg) and subject to BRET analysis. PKA-R, which does not bind SMO, serves as a negative control (see **Fig 4**). Bottom: Following BRET analysis, cell lysates were separated by SDS-PAGE and probed with anti-PKA-C antibodies to detect total (endogenous + YFP-tagged) PKA-C or anti-YFP antibodies to detect YFP-tagged PKA-C (or -R). Blots were quantified via densitometry to estimate the level of PKA-C-YFP expression relative to endogenous PKA-C in each condition. * = cross reactive band. See “Methods” for more information. (**D**) A clonal IMCD3 cell line in which the endogenous SMO locus was modified to contain a BRET donor (“HiBiT Knock-in”; see **S5A–S5C Fig**) was transiently transfected with 0.9 μg of YFP-tagged PKA-R or an equivalent amount of PKA-C-YFP, corresponding to a 5% increase in PKA-C compared to endogenous levels (see western blots performed with parental IMCD3 cells in (C)). Cells were then subjected to BRET analysis. Parental (non-modified) IMCD3 cells serve as a negative control. (**E**) HEK293 cells were infected with viruses encoding FLAG-tagged SMO674 or SMO566 and YFP-tagged PKA-C or Nbβ2AR80 and treated with increasing concentrations of DSP crosslinker (0, 0.125, 0.25, 0.5, 1, or 2 mM). Following DSP quenching, cell lysis, and FLAG purification of SMO complexes, purified samples were separated on reducing SDS-PAGE. Total protein (top) and in-gel YFP fluorescence scans (bottom) for FLAG eluates are shown. * = copurifying contaminant proteins. Molecular masses are in kDa. Recovery of SMO/PKA-C complexes declines at DSP concentrations above 1 mM, likely because high DSP concentrations induce protein aggregation which decreases soluble protein yields in total cell lysates (see **S6 Fig**). All BRET data are reported as BRET ratios (YFP/nanoluc), and background BRET values derived from cells expressing SMO-nanoluc alone were subtracted from all measurements. (*n* = 3–6 biological replicates per condition; error bars = SEM). The underlying data for this figure can be found under **S3 Data**. The uncropped westerns and protein gels are included in **S8 Data**. See **S1 Table** for statistical analysis. BRET, bioluminescence resonance energy transfer; dCT, distal segment of the cytoplasmic tail; DSP, dithiobis(succinimidyl propionate); IMCD3, inner medullary collecting duct; pCT, proximal segment of the cytoplasmic tail; PKA-C, PKA catalytic subunits; SMO, Smoothened.

Vertebrate SMO may recruit PKA-C to the membrane (**Fig 2**) via a direct protein–protein interaction. Consistent with this hypothesis, *Drosophila* Smo was found to associate with PKA-C subunits [54,55]. These studies, however, did not examine whether PKA-C also interacts with vertebrate SMO, leaving open the question of whether a similar principle applies to vertebrate Hh signal transduction. To allow sensitive detection of protein–protein interactions in living cells without solubilization or wash steps that can disrupt labile interactions in conventional biochemical assays, we used bioluminescence resonance energy transfer (BRET) [56]. We fused SMO to a luciferase energy donor (nanoluc) and PKA-C or other candidate interactors to a YFP acceptor. Upon interaction (i.e., within approximately10 nm of SMO), light produced by luciferase excites YFP (**Fig 3A**, left). The YFP/luciferase emission ratio thus provides a normalized metric for protein interactions with SMO.

SMO and PKA-C produced extremely strong BRET signals that often exceeded those of our NbSmo2 positive control (**Fig 3A**, right). SMO BRET with PKA-C mainly required the pCT but not the dCT (compare SMO657 to SMOΔ561–657) (**Fig 3B**). As expected, SMO BRET with NbSmo2 was efficient regardless of the cytoplasmic tail (CT; **Fig 3B**). We also explicitly examined the exact SMO truncations we used in our SMO/PKA-C imaging analysis (SMO674 versus SMO566, **Fig 2**), confirming that the pCT is essential for both SMO/PKA-C colocalization and BRET (**S4A Fig**). Further truncations within the SMO pCT revealed that amino acids 574 to 657 are crucial for BRET with PKA-C (**S1B and S4B Figs**). SMO and PKA-C therefore interact in living cells in a pCT-dependent manner.

The BRET between SMO and PKA-C reflects a bona fide protein–protein interaction, based on several observations. First, titration of a fixed amount of SMO against a varying amount of PKA-C revealed a saturable BRET response (**S4C Fig**), indicative of specific binding rather than nonspecific crowding-induced collisions. Second, 2 control BRET donors, PTCH1 or the D2 dopamine receptor (DRD2), either failed to exhibit BRET with PKA-C or exhibited only weak, nonsaturable BRET responses (**S4C Fig**). Several other BRET acceptors, including Nbβ2AR80, suppressor of Fused (SUFU) [4], and βarrestins [57], also showed minimal or no BRET with SMO (**Figs 3A and S4D and S4E**). Third, BRET is specific, since it requires defined sequences within SMO (**Figs 3B and S4A and S4B**). Fourth, BRET is unlikely an artifact of protein overexpression, as BRET signals do not correlate with expression levels of acceptor proteins (**S4F Fig**). Taken together, these experiments reveal that BRET between SMO and PKA-C is stringent and efficient.

We next asked whether BRET can occur within a physiological range of SMO and PKA-C expression levels in a more canonical model of vertebrate Hh signal transduction. Working in IMCD3 cells, we found that BRET between SMO and PKA-C occurs even under conditions where the exogenously expressed PKA-C-YFP acceptor increases total PKA-C pools by only 0.04% (**Fig 3C**). Similar to our results in HEK293 cells, little or no BRET occurred with SMO lacking the pCT, or with PTCH1 or DRD2 control donors (**S5A Fig**). We also determined that BRET can occur between PKA-C-YFP and endogenous SMO modified via CRISPR/Cas9 to contain a carboxyl-terminal BRET donor (**Figs 3D and S5B and S5C**). Thus, the SMO/PKA-C interaction reported by BRET occurs at endogenous or near-physiological expression levels of both proteins.

To test whether the SMO pCT is sufficient to interact with PKA-C, we studied a soluble SMO construct containing this domain but lacking the extracellular and 7TM regions (SMO555-674). While the SMO pCT alone did not localize to the plasma membrane (**S5D Fig**), it nevertheless showed specific BRET with PKA-C (**S5E Fig**), albeit at lower levels than SMO containing the 7TM domain (compare to **Fig 3A and 3B**). We therefore hypothesize that the pCT provides the core determinants of PKA-C binding, while other regions of SMO may boost the efficiency of the interaction.

To verify the conclusions of our BRET studies biochemically, we tested whether SMO and PKA-C copurify from detergent-solubilized HEK293 cells expressing both proteins. We found that PKA-C copurified with SMO, and the amount is dramatically enhanced by dithiobis(succinimidyl propionate) (DSP), a membrane-permeable amine-specific crosslinker added prior to cell lysis to stabilize protein complexes. In contrast, Nbβ2AR80 did not copurify with SMO (**Figs 3E and S6**). Consistent with our BRET studies, SMO/PKA-C copurification required the SMO pCT (**Figs 3E and S6**). These data biochemically confirm our BRET findings that SMO and PKA-C interact specifically. SMO/PKA-C complexes may contain additional proteins or lipids. However, SMO and PKA-C copurify in similar quantities, and no other proteins were present at comparable levels other than nonspecific contaminants (**Fig 3E**), suggesting that SMO and PKA-C interact directly.

### SMO interacts with free PKA-C subunits rather than PKA holoenzymes

**Fig 4:**
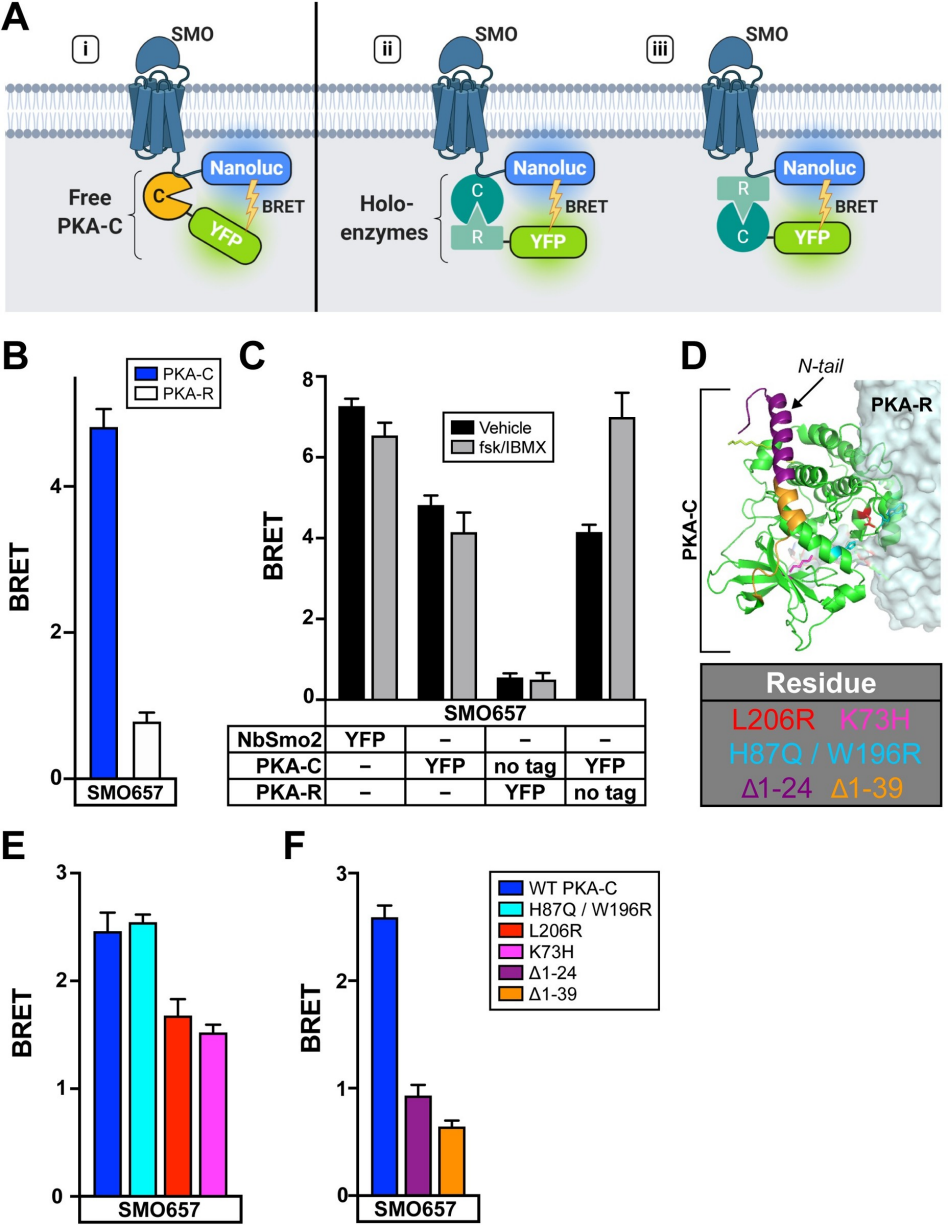
SMO interacts with free PKA-C subunits rather than PKA holoenzymes. (**A**) Schematic of BRET assays to test whether SMO interacts with (i) free PKA-C or intact PKA holoenzymes via (ii) PKA-C or (iii) PKA-R subunits. (**B**) BRET between an SMO657-nanoluc donor and YFP-tagged PKA-C or PKA-R in HEK293 cells. (**C**) HEK293 cells were transfected with an SMO657-nanoluc donor and untagged or YFP-tagged PKA-C or PKA-R subunits, as described in the table. To stimulate cAMP production, cells were treated for 4 hours with forskolin (10 μM) + the phosphodiesterase inhibitor IBMX (1 mM), which blocks cAMP degradation, prior to BRET measurements. (**D**) Structure of PKA holoenzyme (PDB: 4X6R). Key PKA-C residues are colored in the structure and indicated in the table (below). (**E**) BRET between SMO and PKA-C harboring mutations in various regions of the PKA-R binding interface (H87Q/W196R or L206R) or the active site (K73H). (**F**) BRET between SMO and PKA-C harboring deletions of the first 24, or all 39, amino acids from the N-tail. Data are reported as BRET ratios and background-subtracted as in **Fig 3** (*n* = 3–6 biological replicates per condition; error bars = SEM). The underlying data for this figure can be found under **S4 Data**. See **S1 Table** for statistical analysis. BRET, bioluminescence resonance energy transfer; cAMP, cyclic AMP; IBMX, isobutylmethylxanthine; PKA, protein kinase A; PKA-C, PKA catalytic subunits; PKA-R, PKA regulatory subunits; SMO, Smoothened; WT, wild-type.

Many GPCRs employ A-kinase anchoring proteins (AKAPs) to bind PKA holoenzymes via their PKA-R subunits [46,47]. However, our biochemical studies (**Fig 3E**) suggest that SMO interacts directly with free PKA-C without participation from PKA-R. To test this hypothesis, we performed BRET experiments in cells expressing SMO and PKA-C or PKA-R or both (**Fig 4A**). YFP-tagged PKA-R exhibited only weak, nonsaturable BRET with SMO (**Figs 4B and S7A**; see also **Fig 3C**), and coexpression of untagged PKA-C did not increase this signal (**Fig 4C**, Vehicle). These data indicate that PKA-C neither interacts with SMO via PKA-R (**Fig 4A**, iii), nor recruits PKA-R–containing holoenzymes to SMO (**Fig 4A**, ii). Control immunoblots confirmed efficient expression of PKA-R in these experiments (**S7B Fig**; see also **Fig 3C**). SMO therefore does not bind holoenzymes that require cAMP for activation; instead, SMO binds free, catalytically active PKA-C subunits (**Fig 4A**, i).

Although SMO/PKA-C complexes do not directly involve PKA-R, cAMP signals may still affect SMO/PKA-C interactions by dissociating holoenzymes, thereby increasing concentrations of free PKA-C within the cell. We therefore examined the effect of cAMP production on SMO/PKA-C BRET in the presence of untagged PKA-R. cAMP production increased BRET under these conditions (**Fig 4C**). Effects of cAMP production on SMO/PKA-C BRET required PKA-R expression, as expected (**Fig 4C**). We conclude that cAMP can act on PKA holoenzymes to tune the SMO/PKA-C interaction.

PKA-C forms a bilobed structure with an active site involved in substrate binding and phosphoryl transfer on one face and an extended PKA-R binding surface on the opposite face [44,45]. In addition, an N-terminal tail (“N-tail”) region undergoes myristoylation and mediates interactions with accessory factors (**Fig 4D**) [58–61]. A K73H mutation in the active site [62,63] modestly inhibited BRET between SMO and PKA-C. An L206R mutation that affects substrate recognition and the PKA-R binding interface [64] gave similar results, while H87Q W196R [65], which affects a distinct region of the PKA-R binding interface [63], did not block BRET (**Fig 4E**). In contrast, deleting portions of the N-tail significantly reduced BRET between SMO and PKA-C (**Fig 4F**) without affecting PKA-C expression level (**S7C Fig**). These experiments highlight a critical role for the N-tail of PKA-C in mediating interactions with SMO.

### SMO/PKA-C interactions depend on SMO and GRK2/3 activity

PKA phosphorylation of GLI in Hh pathway-responsive tissues or CREB in our heterologous cell assays (**Fig 1**) depends on SMO activity state: It is low when SMO is active and high when SMO is inactive. One way for PKA phosphorylation of these substrates to reflect SMO activity would be for SMO/PKA-C interactions to increase when SMO is active and decrease when SMO is inactive.

**Fig 5:**
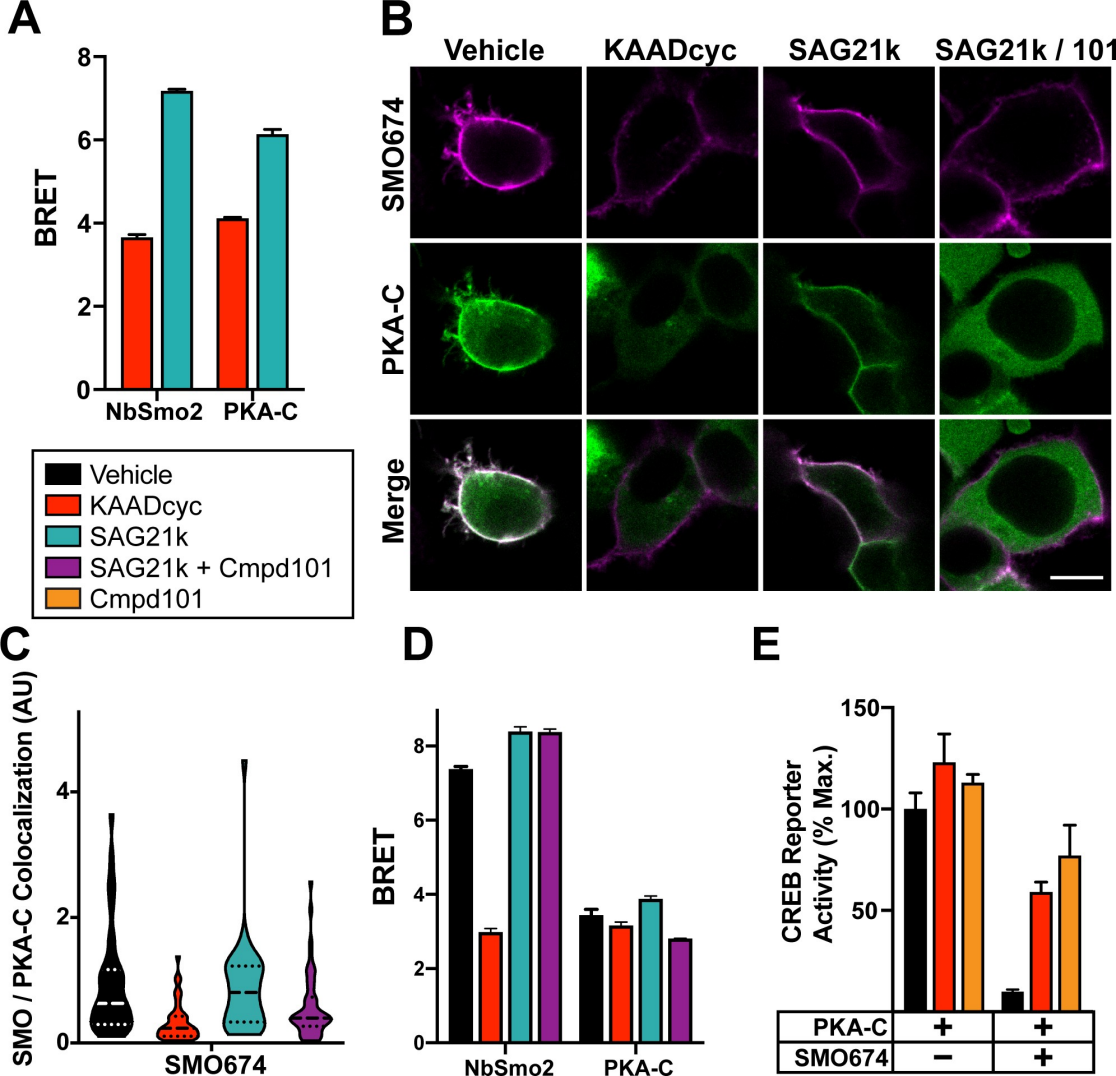
SMO/PKA-C interactions depend on SMO and GRK2/3 activity. (**A**) HEK293 cells transfected with SMO657-nanoluc and PKA-C-YFP were treated with SMO inverse agonist KAADcyc (1 μM) or agonist SAG21k (1 μM) for 1 hour prior to BRET measurements. (**B**) Images of HEK293 cells transfected with FLAG-tagged SMO674 (magenta) and YFP-tagged PKA-C (green) and treated with vehicle, KAADcyc (300 nM), or SAG21k (100 nM) alone or with the GRK2/3 inhibitor Cmpd101 (“101”, 30 μM). Scale bar = 10 μm. (**C**) Quantification of colocalization between SMO and PKA-C for the experiment in (B) (see “Methods’). (**D**) HEK293 cells were transfected with SMO657-nanoluc and YFP-tagged versions of either NbSmo2 or PKA-C and treated with vehicle, KAADcyc (1 μM), or SAG21k (1 μM) for 1 hour or with Cmpd101 (30 μM) for 4 hours. (**E**) Effect of KAADcyc (1 μM) or Cmpd101 (30 μM) on SMO inhibition of the CREB reporter in HEK293 cells. For (E), CREB reporter was normalized to 100%, which reflects reporter activation from PKA-C-transfected cells treated with vehicle. Data in (A), (D), and (E): *n* = 3–6 biological replicates per condition. Error bars = SEM. Data in (C): *n* = 119–216 cells per condition pooled from 2 or more independent experiments. The underlying data for this figure can be found under **S5 Data**. See **S1 Table** for statistical analysis. BRET, bioluminescence resonance energy transfer; GRK, GPCR kinase; Cmpd101, Compound 101; CREB, cyclic AMP response element binding protein; KAADcyc, KAAD-cyclopamine; PKA-C, PKA catalytic subunits; SMO, Smoothened.

We tested this hypothesis by using BRET to examine the impact of small molecule SMO ligands on SMO interactions with either PKA-C or NbSmo2 (which strongly prefers to bind active SMO over inactive SMO; **S2B and S2C Fig**). In these experiments, we measured interactions over the full dynamic range of SMO activity by comparing effects of a high-efficacy SMO agonist, SAG21k, to those of KAADcyc. SMO BRET with PKA-C or NbSmo2 was high with SAG21k but decreased with KAADcyc (**Fig 5A**). SMO modulators produced similar effects on PKA-C or NbSmo2 membrane colocalization (**Figs 5B and 5C and S8A and S8C**). In these BRET experiments, SMO modulators exerted somewhat weaker effects on PKA-C than they did on the NbSmo2 positive control. Nevertheless, these effects may be highly relevant under physiological conditions in the presence of other regulatory influences (see “Discussion”). In any event, these data indicate that SMO/PKA-C interactions vary with SMO activity state—they are significantly enhanced when SMO is active.

In considering how activation of SMO leads to enhanced PKA-C binding, GRK family kinases emerged as leading candidates for controlling this process. GRKs selectively phosphorylate the active states of many GPCRs on their intracellular domains, thereby triggering interactions with cytoplasmic regulatory factors [66–68]. In keeping with this paradigm, PKA-C binds active SMO via its pCT. Moreover, GRK2 can phosphorylate active SMO [69, 70], and inhibition of GRK2 and GRK3 strongly disrupts Hh signal transduction [71–76].

We tested the effect of GRK2/3 activity on SMO/PKA-C colocalization and binding. The selective GRK2/3 inhibitor Takeda Compound 101 (Cmpd101) inhibited PKA-C colocalization (**Figs 5B and 5C and S8B**) and BRET (**Fig 5D**) with SMO. As a control, Cmpd101 did not affect BRET with NbSmo2 (**Fig 5D**). Furthermore, the decreases in BRET are not attributable to changes in levels of SMO and PKA-C following treatment with SMO modulators or Cmpd101 (**S8D Fig**). Cmpd101, like KAADcyc, also reversed SMO-dependent inhibition of the CREB reporter (**Fig 5E**). Cmpd101 exhibited an IC_50_ of 1.87 μM in this assay (**S8E Fig**), similar to its IC_50_ in other cell-based assays [75,77–79]. Consistent with these pharmacological results, we found that SMO inhibition of PKA-C was drastically reduced in HEK293 cells harboring genetic knockouts of *GRK* genes, and this defect was reversed upon reintroduction of GRK2 into the knockout cells (**S8F Fig**). Taken together, these findings show that GRK phosphorylation mediates the activity-dependent binding of SMO to PKA-C.

### GRK2/3 phosphorylation of conserved SMO pCT residues mediates PKA-C binding

A parsimonious interpretation of the above results is that PKA-C recruitment to SMO is dependent on GRK2/3-mediated phosphorylation of the SMO intracellular domains. To test this hypothesis, we identified GRK2/3 phosphorylation sites in SMO purified from HEK293 cells stimulated with SAG21k, KAADcyc, or Cmpd101. We then tested whether alanine substitution of the corresponding residues blocked PKA-C interactions.

**Fig 6:**
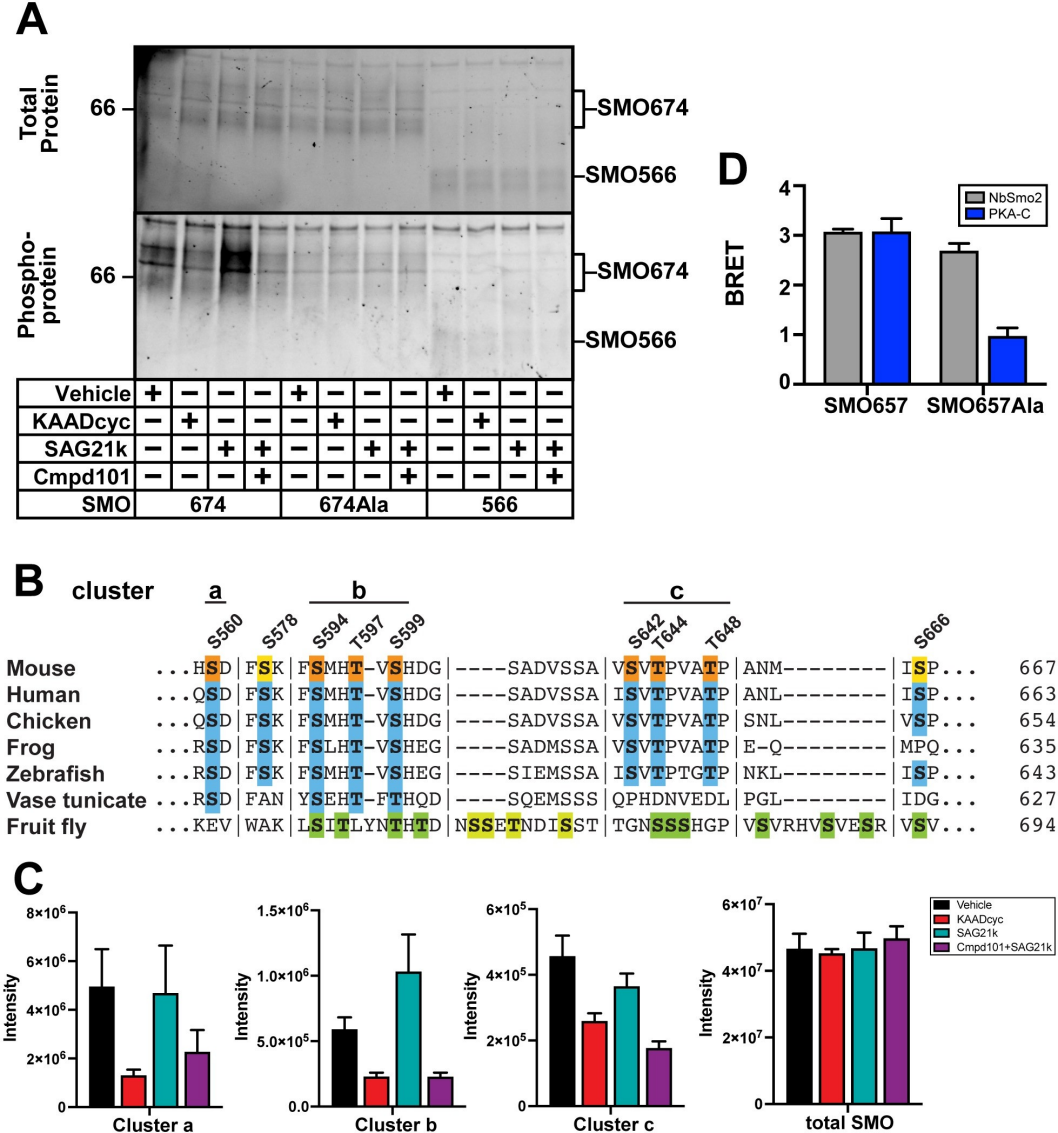
GRK2/3 phosphorylation of conserved SMO pCT residues mediates PKA-C binding. (**A**) HEK293 cells expressing GRK2 and either SMO674 (lanes 1–4), SMO674Ala, which carries mutations in 7 GRK2/3 phosphorylation sites (lanes 5–8), or SMO566 (lanes 9–12). Following treatment with SMO modulators or Cmpd101 (4 hours), SMO was isolated via FLAG affinity chromatography, and total protein or phosphoprotein was visualized using Stain-Free imaging or Pro-Q Diamond staining, respectively. Although GRKs often phosphorylate GPCRs on the intracellular loops of their 7TM domains [67,68], we did not observe phosphorylation within this region of SMO via phosphoprotein staining (A) or MS (**S10 Fig**). Molecular masses are in kDa. (**B**) Clusters of phosphorylated residues identified by MS are labeled above the sequence of mouse SMO. Orange indicates phosphorylation that depends on SMO and GRK2/3 activity, while yellow indicates non-GRK phosphorylation sites. Alignment with SMO from other species reveals sequence conservation (blue), particularly among vertebrates. Green indicates GRK phosphorylation sites previously mapped in *Drosophila* Smo [80]. Vertical lines indicate breaks in sequence. See **S9A Fig** for complete alignment. (**C**) Targeted MS-based quantification of phosphorylation at each of the 3 activity- and GRK2/3-dependent clusters in the SMO pCT (left 3 graphs) and total SMO protein in each sample (right-most graph). “Intensity” is a measurement of the abundance of phosphorylation sites (left) or total protein (right), derived from model-based estimation in MSstats which combines individual peptide intensities (see “Methods”). (**D**) BRET between PKA-C and wild-type SMO657 or SMO657Ala. Data in (C): *n* = 3 biological and 3 technical replicates per condition. Data in (D): *n* = 3–6 biological replicates per condition. Error bars = SEM. The underlying data for this figure can be found under **S6 Data**. The uncropped protein gels are included in **S8 Data**. See **S1 Table** for statistical analysis. Cmpd101, Compound 101; GPCR, G protein–coupled receptor; GRK, GPCR kinase; KAADcyc, KAAD-cyclopamine; MS, mass spectrometry; pCT, proximal segment of the cytoplasmic tail; PKA-C, PKA catalytic subunits; SMO, Smoothened.

Phosphoprotein staining of purified SMO samples revealed SMO activity- and GRK2/3-dependent phosphorylation that required the pCT (**Fig 6A**). We mapped these phosphorylation sites using quantitative mass spectrometry (MS). First, we employed an untargeted strategy to identify and quantify sites within SMO that depended on SMO and GRK2/3 activity and then validated these findings via a targeted MS experiment to quantify phosphorylation with maximal sensitivity, accuracy, and consistency. Our experiments identified 7 sites within 3 clusters (*a*: S560, *b*: S594/T597/S599, and *c*: S642/T644/T648) exhibiting phosphorylation that depended on SMO and GRK2/3 activity. We also uncovered 1 phosphorylation site that exhibited weak dependence on SMO activity but was GRK2/3-independent (S666), along with 1 constitutive phosphorylation site (S578) (**Figs 6B and 6C and S9A and S9B and S2 Table**). Several of these sites are evolutionarily conserved [80], particularly in vertebrates (**Figs 6B and S9A**). Four of these sites (S560, S578, T644, and T648) were not detected in previous studies of vertebrate SMO phosphorylation, which involved in vitro kinase assays with soluble SMO CT fragments [70]. We obtained efficient MS coverage of the entire pCT and all 3 SMO intracellular loops (**S10 Fig**), suggesting that our analysis successfully captured the major phosphorylation sites within these regions of SMO. As the majority of these sites depend on GRK2/3 activity, we conclude that GRK2/3 are the principal kinases that recognize and phosphorylate active SMO in our experiments.

Mutation of the 7 SMO activity- and GRK2/3-dependent phosphorylation sites to alanine substantially reduced SMO phosphoprotein staining (**Fig 6A**) and SMO BRET with PKA-C (**Fig 6D**). As a control, BRET between SMO and NbSmo2 occurred at nearly wild-type levels (**Fig 6D**). These data indicate that GRK2/3 phosphorylation sites in the pCT domain are critical for PKA-C interaction.

### Hh signal transduction is blocked when SMO cannot bind PKA-C

Our heterologous cell model enabled identification and mapping of a GRK2/3-dependent SMO/PKA-C interaction that interferes with PKA phosphorylation of a heterologous soluble transcription factor. To address whether this mechanism contributes to GLI activation in the Hh pathway, we turned to 2 models of Hh signal transduction: (i) activation of a GLI transcriptional reporter in cultured fibroblasts upon treatment with Hh ligands, and (ii) specification of slow muscle cell subtypes during zebrafish development, which is exquisitely sensitive to Hh pathway activity [81–83]. Transduction of Hh signals in these models strictly requires SMO, PKA, and GLI [20,39,83–86] and also depends strongly on the presence of primary cilia [29,87–89].

**Fig 7:**
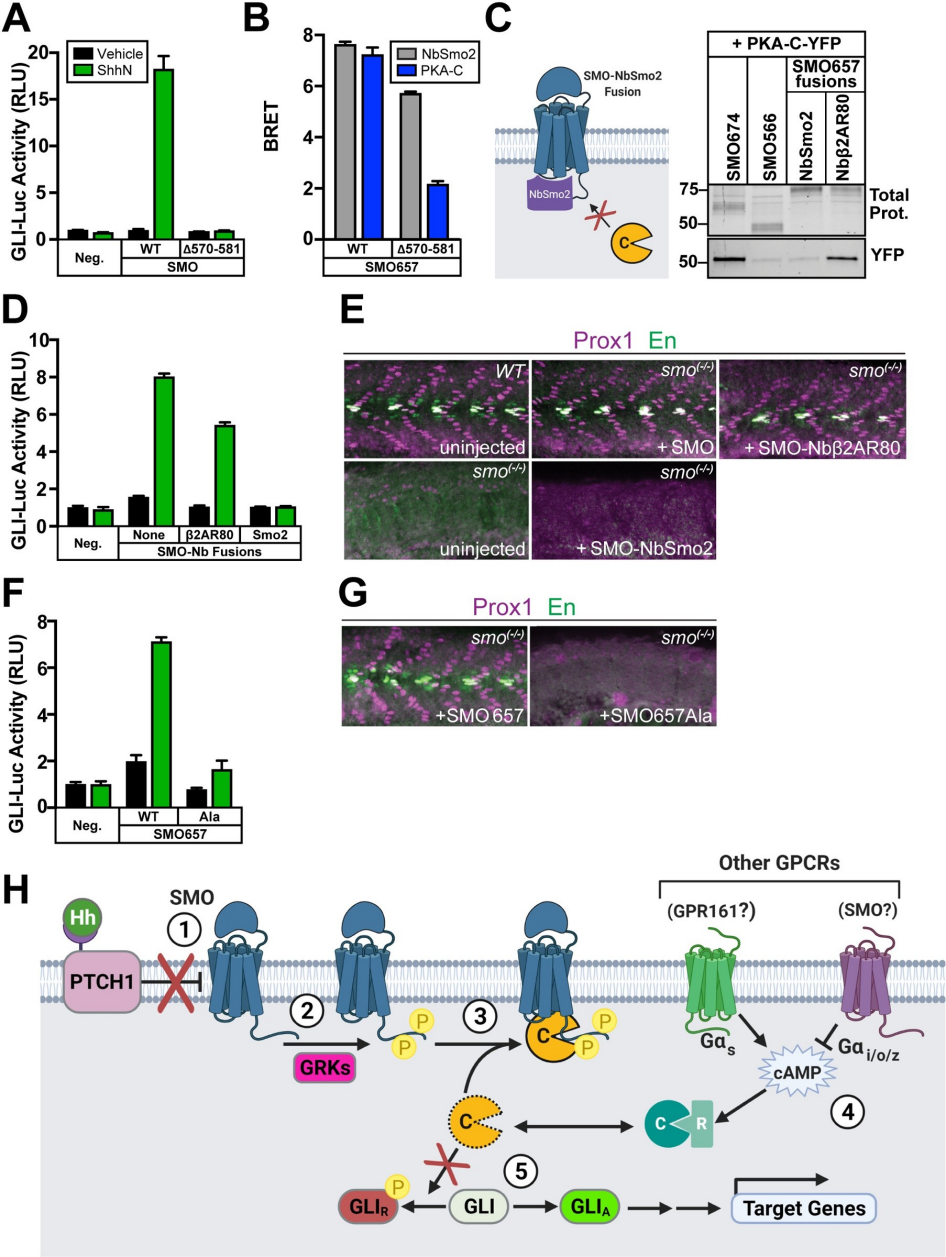
Hh signal transduction is blocked when SMO cannot bind PKA-C. (**A**) GLI transcriptional reporter assay in *Smo*^−/−^ MEFs expressing wild-type SMO or SMOΔ570–581, treated with conditioned medium containing the N-terminal signaling domain of Sonic hedgehog (ShhN, green) or control, non-ShhN-containing conditioned medium (Vehicle, black). GFP serves as a negative control (“Neg.”). (**B**) BRET in HEK293 cells between nanoluc-tagged wild-type or Δ570–581 forms of SMO657 as donor, with YFP-tagged NbSmo2 (gray) or PKA-C (blue) as acceptor. (**C**) Left: Schematic of SMO-NbSmo2 fusion, predicted to block interactions with SMO that require the intracellular face of the 7TM domain. Right: YFP-tagged PKA-C was coexpressed in HEK293 cells with FLAG-tagged SMO674 (lane 1), SMO566 (lane 2), SMO657-NbSmo2 (lane 3), or SMO657-Nbβ2AR80 (lane 4). Following cell lysis and FLAG purification of SMO complexes, samples were separated on SDS-PAGE. Fluorescence scans of total protein (top) and YFP (bottom) in FLAG eluates are shown. Note that DSP crosslinker was not used in this experiment; thus, copurification of PKA-C was less efficient than in **Fig 3E**. Molecular masses are in kDa. (**D**) GLI transcriptional reporter assay in *Smo*^−/−^ MEFs expressing fusions to NbSmo2 or Nbβ2AR80. Non-Nb-fused SMO (“None”) serves as a positive control. (**E**) Confocal images of whole-mount wild-type zebrafish embryos or *smo*^−/−^ mutants injected with mRNAs encoding either wild-type SMO, SMO-Nbβ2AR80, or SMO-NbSmo2. Embryos 26 hpf were fixed and stained with antibodies against Prox1 (magenta) or En (green) to mark populations of muscle fiber nuclei. (**F**) GLI transcriptional reporter assay in *Smo*^−/−^ MEFs expressing wild-type SMO657 (“WT”) or SMO657Ala. (**G**) Zebrafish were injected with mRNAs encoding wild-type SMO657 or SMO657Ala, then stained for muscle fiber nuclei as described in (E). (**H**) Proposed model for SMO activation of GLI via PKA-C membrane sequestration: (1) Hh proteins bind to and inhibit PTCH1, inducing an activating conformational change in SMO; (2) Active SMO is recognized and phosphorylated by GRKs; (3) Phosphorylated SMO recruits PKA-C to the membrane, preventing PKA-C from phosphorylating and inhibiting GLI; (4) GPCRs that couple to Gα_s_ (such as GPR161) [92] or Gα_i/o/z_ (perhaps including SMO itself, which can couple to Gα_i/o/z_ [32,33,40–42]) can raise or lower cAMP levels, respectively, thereby affecting SMO/PKA-C interactions by regulating the size of the free PKA-C pool. (5) GLI is converted from a repressed (GLI_R_) to an active (GLI_A_) form and regulates transcription of Hh target genes. Data in (A), (B), (D), and (F): *n* = 3 biological replicates per condition. Error bars = SEM. Data in (E) and (G): *n* = 78 (SMO), 61 (SMO-NbSmo2), 63 (SMO-Nbβ2AR80), 62 (SMO657), 70 (SMO657Ala), and 100 (uninjected). The underlying data for this figure can be found under **S7 Data**. The uncropped protein gels are included in **S8 Data**. See **S1 Table** for statistical analysis. cAMP, cyclic AMP; DSP, dithiobis(succinimidyl propionate); En, Engrailed; GLI, glioma-associated; GPCR, G protein–coupled receptor; Hh, Hedgehog; hpf, hours postfertilization; MEF, mouse embryonic fibroblast; PKA-C, PKA catalytic subunits; RLU, relative luciferase unit; ShhN, N-terminal signaling domain of Sonic hedgehog; SMO, Smoothened.

First, we deleted a small stretch of sequence (SMOΔ570–581) that lies within a region of the pCT critical for interaction with PKA-C (**S4B Fig**). This SMO deletion abolishes activation of GLI in cultured *Smo*^−/−^ fibroblasts [30,39] (**Fig 7A**) without affecting SMO ciliary localization [30]. Accordingly, the Δ570–581 deletion severely reduced SMO BRET with PKA-C (**Fig 7B**). BRET with NbSmo2 was substantially less affected (**Fig 7B**), suggesting that the defect in PKA-C interaction does not stem from issues with expression or ability to assume an active conformation. Thus, SMOΔ570–581 fails to bind PKA-C and to activate GLI.

Next, we harnessed our insight that SMO/PKA-C interactions depend on SMO activity and GRK2/3 phosphorylation to design a different non-PKA-C-binding SMO mutant. The intracellular region of the SMO 7TM domain changes conformation dramatically upon SMO activation [90]. An analogous region is also necessary for recruitment of GRKs to the active states of other GPCRs [67,91]. To assess whether SMO interactions with PKA-C also required this region, we fused NbSmo2 to the end of the CT. As a result, NbSmo2 is expected to bind SMO and dissociate minimally, if at all; interactions with the 7TM domain’s intracellular region that involve other proteins, domains of SMO, or both, will be efficiently blocked (**Fig 7C**, left). SMO-NbSmo2 failed to bind PKA-C, while a negative control fusion of similar size and expression level, SMO-Nbβ2AR80, bound robustly (**Fig 7C**, right). In cultured *Smo*^−/−^ fibroblasts, SMO-NbSmo2 also failed to stimulate GLI-dependent transcription in response to Hh ligands, whereas SMO-Nbβ2AR80 still mediated strong transcriptional responses (**Fig 7D**). In zebrafish, expression of mRNA encoding wild-type SMO or SMO-Nbβ2AR80 restored Hh pathway–dependent muscle development to *smo*^−/−^ embryos, whereas SMO-NbSmo2 did not (**Fig 7E**). Control experiments confirmed that SMO-NbSmo2 and SMO-Nbβ2AR80 accumulate normally in cilia in response to SMO activation (**S11A Fig**). These findings argue that blockade of Nb-binding regions in the SMO 7TM domain hinders interactions with PKA-C and activation of GLI. They also further establish a correlation between SMO/PKA-C binding and GLI activation.

Lastly, we mutated the 7 critical GRK2/3-dependent phosphorylation sites in SMO. These mutations not only dramatically impaired SMO-dependent GLI transcription in cultured *Smo*^−/−^ fibroblasts (**Fig 7F**) but also failed to restore normal muscle specification to *smo*^−/−^ zebrafish embryos (**Fig 7G**). Control experiments showed that these mutations did not block SMO localization to cilia, ruling out ciliary trafficking defects as possible trivial explanations for these phenotypes (**S11B and S11C Fig**).

Taken together, these results highlight an important role for GRK2/3-mediated SMO/PKA-C binding and subsequent PKA-C membrane sequestration in controlling GLI activation in cellular models as well as embryonic patterning in vivo.

## Discussion

We have identified and characterized a novel interaction between vertebrate SMO and PKA-C, demonstrated how this recruits PKA-C to membranes, and showed how it can dramatically affect the activity of PKA-regulated transcription factors as well as GLI-dependent outputs in cultured cells and in vivo. These insights enable a deeper understanding of how Hh signal transduction orchestrates cell proliferation and differentiation events in nearly all of its biological roles.

Based on these findings, we propose the following mechanism (**Fig 7H**). In the pathway “off” state, SMO is inactive and inefficiently binds PKA-C. PKA-C is thus available to phosphorylate and inactivate GLI. In the pathway “on” state, SMO activation triggers GRK phosphorylation of the pCT, increasing PKA-C binding and siphoning PKA-C to the membrane. SMO-bound PKA-C cannot access soluble GLI substrates. This leads to loss of inhibitory GLI phosphorylation, which could occur via GLI protein turnover [12,22,24], tonic action of phosphatases on GLI [93], or both.

Our proposed mechanism helps to explain key aspects of SMO-to-GLI communication not easily reconciled with existing models. Prior studies have invoked a variety of explanations for how SMO might activate GLI [4], including (i) utilizing canonical Gα_i/o/z_ and cAMP-dependent pathways to inhibit PKA [14]; (ii) controlling ciliary cAMP, and thus PKA-C, by influencing the ciliary localization of GPR161, a constitutively active *Gα*_*s*_-coupled GPCR [92]; and (iii) interacting with βarrestin1/2 [94] or Ellis van Creveld protein 2 (EVC2) [95], which might regulate GLI by as yet undefined mechanisms. However, the role of Gα_i/o/z_ signaling in SMO–GLI communication remains a matter of debate, as several studies found that pharmacological or genetic inactivation of Gα_i/o/z_ signaling only partially or incompletely blocks SMO-mediated GLI activation [33–36,96]. In addition, whether SMO activation reduces ciliary cAMP remains controversial [36,97,98]. Finally, mouse knockouts of *βarrestin1/2* [99], *EVC2* [100], or *GPR161* [92,101,102] fail to exhibit the severe, widespread developmental defects expected with disruption of core Hh pathway components [20,103,104]. Therefore, existing models neither fully explain how SMO activates GLI nor rule PKA in or out as a mediator of this process. Our work reveals a route by which SMO can affect PKA substrate phosphorylation that does not absolutely require any of these factors, offering an explanation for prior conflicting observations.

Our use of a simplified experimental system enabled us to uncover an SMO/PKA-C regulatory mechanism that had been overlooked previously. Nevertheless, the approaches we employed have clear limitations. For example, heterologous reconstitution does not necessarily capture all aspects of Hh signal transduction under physiological conditions. In addition, while our study focused on SMO-mediated PKA-C membrane sequestration, it does not rule out possible additional routes by which SMO could affect PKA-C phosphorylation of GLI, such as altering PKA-C stability, enzymatic activity, or substrate specificity. Future reconstitution-based studies may allow us to further understand the biochemical mechanism by which SMO affects PKA-C signaling and study how other Hh pathway components influence this process.

### GRKs and Hh signal transduction

Our work also sheds light on how GRKs control Hh signal transduction. Pathway activation strongly requires these kinases in vertebrates, but their underlying target and mechanism of action remained poorly defined [4,72,75,76,105]. While GRKs can phosphorylate SMO [69], mutation of the previously mapped GRK sites [70] to alanine does not disrupt embryonic patterning in vivo [76]. Consequently, it was unknown whether the physiological target of GRKs in the Hh pathway is SMO or a different protein altogether [4,75,76,105]. Furthermore, how GRK phosphorylation of its substrate(s) regulates GLI activity remained unclear. A key limitation is that prior studies defined GRK sites based largely on in silico predictions or in vitro kinase assays with soluble SMO CT fragments [70]. GRKs lack a strict consensus motif, and capturing physiological activity-induced phosphorylation of GPCRs requires their 7TM domains to be embedded in a membrane lipid environment [91,106–108], making phenotypic interpretation of existing alanine mutants difficult.

Here, we studied phosphorylation of SMO (with an intact 7TM domain) expressed in mammalian cells. We also used specific pharmacological agents to define the activity and GRK dependence of SMO phosphorylation events. Our analysis revealed several GRK phosphorylation sites in the pCT that were not previously reported. KAADcyc and Cmpd101 block phosphorylation to similar extents at nearly all of the sites we identified in the pCT. This indicates that GRK2/3 are the principal kinases that mediate SMO activity-dependent pCT phosphorylation in our system (although other kinases might also phosphorylate these sites in an SMO activity-independent manner, perhaps priming them for phosphorylation by GRK2/3). Furthermore, mutation of the sites we identified strongly affects GLI activation in cultured cells and in vivo, indicating that GRK phosphorylation of SMO is in fact critical for Hh signal transduction. Although GRKs may play multiple roles in Hh signal transduction [75,76,105], our PKA-C sequestration model is particularly appealing because it directly links GRK phosphorylation of SMO to stimulation of GLI.

A future challenge is to understand how GRK2/3 recognize and phosphorylate the SMO active state. Generally speaking, binding of an activating ligand to a GPCR’s 7TM domain induces an outward movement of transmembrane helices 5 and 6 (TM5-6), along with the intervening third intracellular loop (ICL3). GRKs recognize and transiently bind the GPCR active state, in part via interactions with ICL3. This activates the GRK and induces phosphorylation of the GPCR’s cytoplasmic domains. Because SMO activation by sterol ligands also induces outward movement of TM5-6 and ICL3 [90], we hypothesize that GRK interactions with SMO will follow the same general paradigm described above for other GPCRs. In this regard, the SmoNb2 fusion (Fig 7) might disrupt SMO/PKA-C binding by binding the intracellular surface of active SMO and interfering with some aspect of the GRK recognition process (although at present we cannot exclude the possibility that the Nb directly occludes an interaction between PKA-C and SMO ICL3). Future in vitro reconstitution of SMO-GRK complexes, and determination of their structures, will delineate the GRK phosphorylation process in more detail.

### Structural determinants of the SMO/PKA-C complex

SMO activation of GLI requires the pCT domain [38,39], but for reasons that have remained incompletely understood. The pCT contains essential ciliary trafficking motifs [38]. However, pCT mutations such as Δ570–581 disrupt GLI activation without affecting SMO ciliary trafficking [30], indicating that the pCT plays additional indispensable roles in GLI activation besides controlling SMO ciliary localization. Here, we show that one such function is to bind and sequester PKA-C when SMO and GRK2/3 are active. Structures of the pCT or of the SMO/PKA-C complex have not yet been reported. However, our mutational analysis suggests that this complex involves, at minimum, the phosphorylated pCT of SMO and the N-tail domain of PKA-C. Membrane lipid interactions may also contribute to these complexes, as the N-tail is myristoylated which can increase PKA-C membrane association in some settings [58, 63,109,110]. Intriguingly, recent structures of non-SMO GPCRs in complex with βarrestins have also revealed critical interactions with the receptor’s phosphorylated cytoplasmic tail and lipids in the surrounding membrane [111,112]. Thus, distinct GPCRs may engage a diverse set of downstream effectors, such as βarrestins or PKA-C, using similar structural principles.

### SMO control of PKA localization as an evolutionarily conserved rheostat?

The Hh pathway controls development and regeneration throughout the animal kingdom [3], but whether the underlying transduction mechanism is conserved remains a matter of debate [113]. Recent studies of SMO communication with GLI have emphasized aspects of the Hh pathway that are uniquely important to mammals but not insects, such as the primary cilium [28,113,114]. In contrast, the SMO/PKA-C interaction we describe here is conserved in *Drosophila* [54,55]. This interaction promotes *Drosophila* Hh pathway activation in part by titrating PKA-C out of a protein complex that promotes phosphorylation and inhibition of the GLI ortholog Ci [54,55]. This action is strikingly parallel to effects of SMO/PKA-C interactions on CREB reporter activation and GLI-dependent transcription observed here. Thus, SMO may utilize PKA to communicate with GLI via mechanisms that are more similar between species than previously appreciated. Besides its well-characterized inhibitory role in phosphorylating and inactivating Ci, PKA positively influences Hh signal transduction in *Drosophila*; it phosphorylates the Smo C-tail, thereby promoting binding of downstream accessory factors including the atypical kinesin Costal2 (Cos2). This positive role of PKA-C appears to be unique to *Drosophila*, as the PKA-C phosphorylation sites are not conserved in vertebrate SMO [20,115]. Nevertheless, it remains possible that PKA-C promotes vertebrate Hh signal transduction by phosphorylating the SMO pCT or dCT at sites that are, as of yet, unidentified.

We speculate that a mechanism based on direct SMO/PKA-C interactions is well suited to Hh signal transduction, as it may help to ensure graded blockade of PKA signaling over a broad range of SMO activity levels. As a result, SMO would acquire the capacity to accurately translate differences in amounts of extracellular Hh proteins (via PTCH1 binding and inactivation) into discrete changes in GLI activity. This is essential for Hh proteins to function in gradients as concentration-dependent (morphogenetic) signals in the limb bud, neural tube, and elsewhere. In contrast, with cascades that involve intermediary components present in limiting amounts, downstream responses may reach maximal levels even when upstream receptors are not fully activated [116], causing a loss of signal fidelity at high levels of receptor activity. Future studies of SMO/PKA-C interactions during embryonic development will help to address these hypotheses.

### The role of the cilium in SMO regulation of PKA activity

In our HEK293 model, SMO activation triggered changes in the interactions with and localization of a substantial fraction of cellular PKA-C. In contrast, under physiological conditions, SMO would mainly act on the much smaller pool of PKA-C in the primary cilium. In this manner, SMO could specifically regulate GLI transcription factors without affecting PKA-dependent processes elsewhere in the cell [20,92,97,98]. Our live-cell imaging studies revealed that SMO and PKA-C colocalize in the cilium, supporting the hypothesis that SMO has the capacity to act on ciliary PKA-C. These findings are consistent with a recent study which specifically inhibited PKA-C at defined locations within the cilium, revealing a labile pool of cilioplasmic PKA-C that is critical for GLI regulation [51]. Along similar lines, PKA-C activity has been detected within the cilioplasm using a FRET-based sensor of PKA substrate phosphorylation [36]. Interestingly, prior studies using conventional immunofluorescence microscopy did not report SMO/PKA-C colocalization in cilia [20,26]. The basis for this discrepancy is uncertain, but may arise from disruption of SMO/PKA-C complexes during sample fixation or permeabilization.

Upon Hh pathway activation, SMO not only changes conformation within the cilium [117,118], but also accumulates approximately 20-fold in the ciliary membrane [27,30,31]. This increase in SMO abundance, along with the SMO activity-dependent binding events described in our study, may synergize to effectively sequester a pool of ciliary PKA-C at the membrane, away from GLI proteins in the cilioplasm. In some of our experiments (e.g., **Fig 1B**), SMO interaction with and regulation of PKA-C appears to depend only partially on SMO activity state. However, a key limitation of our HEK293 system is that it underestimates the activity dependence of SMO/PKA-C interactions under physiological conditions. This is because our HEK293 studies measure interactions between overexpressed SMO and PKA-C in the plasma membrane, and therefore do not take into account the fact that SMO levels dramatically increase in the cilium during Hh pathway activation. As a result, SMO/PKA-C interactions are expected to acquire additional SMO activity dependence in the context of the cilium.

How might SMO/PKA-C interactions block GLI phosphorylation in the cilium? This process may entail transfer of PKA-C out of ciliary protein complexes that facilitate GLI phosphorylation and inhibition and into SMO-containing complexes that do not. Precedent for this idea comes from Cos2 in *Drosophila*, which binds PKA-C and Ci to promote phosphorylation of Ci in the pathway “off” state; activated SMO then recruits PKA-C, thereby rearranging the complex to relieve Ci phosphorylation [54,55,119]. Consistent with this hypothesis, Kif7, the vertebrate Cos2 ortholog, interacts with GLI [120–123]. Interestingly, both GLI and KIF7 traffic to the tip of the cilium upon Hh pathway activation [29,30,123,124]. Kif7 might bind PKA-C and GLI near the base of the cilium to favor phosphorylation and inactivation of GLI in the pathway “off” state. In the pathway “on” state, active SMO accumulates in cilia, which could recruit PKA-C out of these Kif7-containing complexes. The subsequent loss of GLI phosphorylation could trigger Kif7–GLI complexes to engage ciliary trafficking machinery and ultimately accumulate at the cilium tip. While the above model is appealing from an evolutionary perspective, it remains speculative at present, in part because Kif7/PKA-C interactions have not yet been reported.

### SMO control of GLI may require several mechanisms acting in concert

The mechanism we identified likely acts together with other processes to enable SMO activation of GLI. For example, SMO or other GPCRs may still utilize G proteins to set levels of cAMP, and thus levels of free PKA-C, within a critical range that allows SMO/PKA-C interactions to affect GLI activity (**Fig 7H**). Within this range, PKA-C could access GLI when SMO is inactive but undergo efficient membrane sequestration when SMO is active. This concept has precedent in a prior study which concluded that SMO utilizes 2 signals to activate GLI: a G_i/o_-dependent signal that involves the 7TM domain and a second signal which originates in the SMO cytoplasmic tail [32] (the domain shown here to bind PKA-C.) It is also consistent with observations that manipulation of cAMP signals, via expression of dominant negative (non-cAMP-binding) PKA-R constructs or treatment with forskolin [16, 86,125,126], strongly increases or decreases GLI activity, respectively. Along similar lines, knockout of GPR161 elevates Hh pathway activity in some settings [75,92,101,102]. On the other hand, expression of a constitutively active (non-PKA-R-binding) PKA-C mutant, such as H87Q W196R [125], expands the free PKA-C pool to a point where it can no longer be effectively sequestered by SMO. Thus, a number of processes may cooperate with the SMO mechanism described here to create a robust PKA-C activity switch. In this regard, while SMO or GRK2/3 modulators exert modest effects on SMO/PKA-C interactions and colocalization in some of our experiments, they may dramatically affect PKA-C substrate phosphorylation under physiological conditions where other regulatory influences are present.

### GPCR signaling without second messengers

The mechanism we describe here for the Hh pathway may apply more generally to communication between GPCRs and PKA. These receptors and effectors participate in numerous signaling cascades that mediate an extraordinarily diverse range of biological processes [45,46,127,128]. Yet, it remains unclear how communication between just 2 types of signaling molecules can produce such a vast array of cellular and physiological outputs. Prior studies have focused largely on indirect modes of GPCR–PKA communication involving G proteins, cAMP, and AKAP adaptors [46,128]. In contrast, our study describes an alternative mechanism, based on direct PKA-C interactions with an active GPCR. This mechanism may act in concert with classical second messenger signals to bias phosphorylation of PKA substrates toward or away from specific subcellular locations. Such receptor-mediated PKA sequestration may constitute a broader theme among GPCRs in the cilium, as was recently shown for GPR161, which encodes an AKAP domain in its intracellular carboxyl terminus that binds and recruits PKA-R subunits to the ciliary membrane [129]. The additional level of spatial regulation gained from these strategies may allow GPCR-containing pathways throughout the cell to encode new types of downstream responses, thereby permitting control of an expanded array of biological outputs.

## Methods

### Supplemental information on experimental model system

#### Use of CREB reporter assay to study SMO regulation of PKA-C

Hh signal transduction is often studied using GLI transcriptional readouts [86]. These readouts present 2 major obstacles for determining whether SMO inhibits PKA to activate GLI. First, GLI transcription is strongly affected by manipulation of either SMO or PKA [4,12,86], complicating efforts to determine whether SMO and PKA reside in the same linear pathway or constitute 2 separate influences on GLI. Second, during Hh signal transduction, SMO and GLI are subject to elaborate ciliary trafficking mechanisms [28,114] that are incompletely understood and difficult to disentangle from the events occurring immediately downstream of SMO activation. To strip away these potentially confounding factors, we developed a heterologous HEK293 model for SMO regulation of PKA activity. This approach permits simple, direct measurements of SMO effects on PKA, independent of ciliary trafficking or other intermediate steps [42]. We used CREB transcription to monitor PKA phosphorylation in HEK293 cells. CREB, like GLI, is a soluble transcription factor regulated by PKA phosphorylation (although PKA phosphorylation activates CREB but inhibits GLI.) However, CREB is not known to be subject to the other major mechanisms that regulate GLI activity [12, 37]. Therefore, any effects of SMO on CREB transcription would provide evidence that SMO can control PKA. In addition, unlike GLI, activation of CREB transcription factors is not reported to require the primary cilium. As a result, we can directly study SMO effects on PKA function in the absence of ciliogenesis or ciliary protein trafficking processes.

#### Assumptions regarding stoichiometry of SMO/PKA-C complexes in cilia

Prior studies support the idea that a small but critical pool of free PKA-C exists in the cilium in the pathway “off” state and that levels of SMO in the cilium exceed those of free PKA-C in the pathway “on” state.

First, in the absence of Hh, PKA-C phosphorylates and inactivates GLI, likely at the ciliary base or cilioplasm. It follows that some amount of free PKA-C must exist in the cilium in the pathway “off” state; this is consistent with the constitutive Hh pathway activation induced by blocking endogenous ciliary PKA-C (via targeting PKA inhibitor peptides to the cilium (see [51], and refer to “The role of the cilium in SMO regulation of PKA activity” in our “Discussion” section above)). Such a pool of free PKA-C may arise, for example, from cAMP microdomains or nanodomains, trafficking of free C subunits into GLI-containing protein complexes, or both. Regardless of the underlying mechanism, prior literature strongly supports a role for free PKA-C in cilia phosphorylating and inactivating GLI. Second, a recent study of the ciliary proteome [130] readily detected SMO, but failed to detect PKA-C. Given the evidence for free PKA-C in cilia (see above), a parsimonious interpretation is that (1) levels of free PKA-C in cilia, while sufficient to phosphorylate and inactivate GLI, must be extremely low in absolute terms; and (2) levels of SMO likely exceed those of PKA-C, particularly in the pathway “on” state in which steady-state SMO levels dramatically rise in cilia. This scenario fits with the current view of the *Drosophila* Hh pathway [54,55], in which a subpopulation of catalytically active PKA-C resides in a complex with Ci, but is efficiently sequestered away from Ci once Smo accumulates at the plasma membrane in the pathway “on” state.

The question of SMO:PKA-C stoichiometry in cilia is by no means resolved and requires further investigation once more sophisticated tools for measuring the relevant populations of these proteins become available. Nevertheless, our assumptions that free PKA-C exists in cilia and that SMO levels can exceed those of PKA-C specifically in the cilium in the pathway “on” state are both supported by existing work in the field.

#### Cartoons

The cartoons utilized in this manuscript were created with BioRender.

#### Antibodies

Our studies employed the following primary antibodies: Rabbit monoclonal anti-Engrailed (Monoclonal Antibody Facility, Institute of Neuroscience, University of Oregon), mouse polyclonal anti-Prox1 (AngioBio, 11-002P), mouse anti-FLAG M1 (prepared in-house, 1:5,000), rabbit anti-FLAG M2 (Sigma, F7425, 1:1,000), rabbit anti-GFP (which also detects YFP) (Thermo Fisher Scientific A11122, 1:5,000), mouse anti-PKA-C (BD Biosciences, 610980, 1:5,000), mouse anti-PKA-R (BD Biosciences, 610609, 1:500), mouse anti Arl13b (Antibodies, 75–287, 1:1,000), rabbit anti-RFP (Thermo Fisher Scientific, R10367, 1:1,000), rabbit anti-CREB (Cell Signaling Technology, 9197S, 1:1,000), and rabbit anti phospho-CREB (Cell Signaling Technology, 9198S, 1:1,000). For chemiluminescent western blots, HRP-conjugated anti-mouse and anti-rabbit secondary antibodies were obtained from Promega and used at 1:20,000. For infrared western blots, IR680- and IR800-conjguated secondary antibodies (LiCor) were used at 1:20,000. For immunofluorescence, AlexaFluor-conjugated secondary antibodies were obtained from Thermo Fisher Scientific and used at 1:1,000. M1 FLAG affinity resin and M1 FLAG-Alexa 647 conjugates were prepared in house.

#### Bacterial and yeast strains

TOP10 cells were obtained from Thermo Fisher Scientific (C404010). Yeast strain BJ5465 for Nb selection was previously described [131].

#### Cell lines

HEK293-Freestyle (R79007) and HEK293FT (R70007) cell lines were obtained from Thermo Fisher Scientific. HEK293S GnTI- cells were a gift from K.C. Garcia. HEK293 Gα-null cells were a gift from A. Inoue and were previously described [43]. HEK293A parental and GRK2,3,5,6 knockout cells (ΔGRK cells) were generated via CRISPR/Cas9 and will be described in a separate publication. 4C20 *Smo*^−/−^ MEFs were previously described [39]. HEK293-ShhN stable cells were previously described [132]. IMCD3 Flp-in parental cells were a gift from P. Jackson.

### Cell culture

*Smo*^−/−^ MEFs and adherent HEK293FT cells were grown in a 37°C, 5% CO_2_ incubator and maintained in DMEM with 10% FBS and 1% penicillin/streptomycin/glutamine. ΔGRK cells and their parental 293A counterparts were grown under the same conditions. HEK293 Gα-null cells were grown in the same medium, but on plates coated with 0.3 mg/ml collagen. HEK293-Freestyle cells were grown in suspension culture in an 8% CO_2_ incubator equipped with a shaking platform and maintained in 293-Freestyle medium with 1% FBS. Sf9 cells were maintained in suspension culture at 27°C using Sf900-III with 10% FBS and 10 μg/ml gentamicin. IMCD3 cells were maintained in DMEM/F12 with 10% fetal bovine serum and 100X pen-strep; stable clones were made via cotransfection of pEF5-FRT constructs with Flp recombinase, followed by selection in 5 μg/ml blasticidin as previously described [51]. For the cilia imaging studies in S11A Fig, *Smo*^−/−^ MEFs stably expressing the indicated constructs (in MSCV-puro) were selected in puromycin and single-cell cloned.

#### Chemicals, cell culture reagents, and other supplies

KAADcyc was obtained from Toronto Research Chemicals (K171000). Carbachol was obtained from Cayman Chemical (14486). SAG21k was a gift from P. Beachy. IBMX (3-isobutyl-1-methylxanthine) was obtained from Sigma-Aldrich (I5879). Forskolin was obtained from Sigma-Aldrich (F3917). Coelenterazine h was obtained from NanoLight Technology (301–500). Cmpd101 was obtained from Hello Bio (HB2840). DMEM (11965118), PBS (10010049), Trypsin (25300–120), pen-strep-glutamine (10378016), and HBSS (24020117) were obtained from Thermo Fisher Scientific. Protease inhibitor tablets (A32955) and protease/phosphatase inhibitor cocktail (78441) were obtained from Thermo Fisher Scientific. FBS was obtained from Omega Scientific (FB-02). Poly-d-lysine (P0899) and collagen (C3867) were obtained from Sigma-Aldrich. FLAG peptide was custom synthesized by GenScript. Alexa 647 NHS ester (A20006) was obtained from Thermo Fisher Scientific. CNBr sepharose was obtained from GE Healthcare (17-0981-01). Pro-Q Diamond kit was obtained from Thermo Fisher Scientific (MPP33300). Moreover, 35-mm dishes for live cell imaging of HEK293 cells were obtained from Cellvis (D35-14-1.5-N). Also, 8-well chamberslides for live cell imaging of IMCD3 cells were obtained from ibidi (80826). TransIT 293 (MIR 2705) and TransIT 2020 (MIR 5405) were obtained from Mirus Bio. Lipofectamine 2000 (11668019) was obtained from Thermo Fisher Scientific. Furimazine was obtained from AOBIOUS (AOB36539). Nano-Glo HiBiT Lytic Detection System (N3030) and HiBiT Blotting System (N2410) were obtained from Promega.

### Molecular biology

For GLI-luciferase assays and zebrafish rescue experiments, SMO was cloned into pEF5-FRT vector with N-terminal tdTomato tag (**S1C and 7D and 7F Figs**) or the pGEN vector with a carboxyl-terminal his and myc tags (**Fig 7A**); the SMOΔ570–581 construct (**Fig 7A**) was previously described [30]. For CREB assays, BRET assays, and experiments involving SMO purification, SMO was cloned into pVLAD6. All mouse SMO constructs in pVLAD6 are truncated at residues 1 to 64 and contain a heterologous HA signal sequence and a TEV protease cleavage site, as previously described [90]. In addition, the BRET constructs also include a flexible linker followed by nano-luciferase (nanoluc) tag at the carboxyl terminus and an N-terminal SBP purification tag between the FLAG tag and the TEV site. The PTCH1-B truncation mutant [42] was fused to nanoluc via a similar strategy and cloned into pVLAD6. All other SMO truncations are described in **S1B Fig** and tagged according to the text. SMO-Nb fusion constructs contained a flexible linker, followed by either NbSmo2 or Nbβ2AR80; for the constructs in **Fig 7E**, an APEX2 tag was also included. In **Fig 7D**, as a control for the effects of Nb fusions, a fusion to Gα_o_, which activates GLI transcription as efficiently as non-fused SMO [42], was used (“None”). The SMO566-Gα_o_ construct was previously described [42,90]. For soluble SMO C-tail constructs, amino acids 555 to 674 (**S1B Fig**) were fused at their N-terminus to protein C and their carboxyl terminus to a nanoluc, with flexible linkers between each element, and cloned into pVLAD6. Nbβ2AR80, NbSmo2, NbSmo8, PKA-Cα, PKA-RIα, or SUFU were each fused to a flexible linker and GFP or YFP and cloned into pVLAD6. YFP-tagged βarrestin1 and 2 in pcDNA3.1zeo were obtained from Addgene (catalog 36916 and 36917). Full-length wild-type PTCH1 in pRK5 was previously described [133]. Human DRD2 and bovine GRK2 were obtained from Addgene (catalog # 66269 and 14691, respectively), amplified by PCR, fused to nanoluc or GFP (respectively), and cloned into pVLAD6. Constructs for PKA-RIα and PKA-C (in pCMV-SPORT6) were acquired from Transomic (product # BC005697 and BC054834, both are *Mus musculus*). Human SUFU was amplified from a pRK5 expression construct (gift from P. Beachy). The m2AchR plasmid was obtained from the University of Missouri cDNA Resource Center. LgBiT was amplified from existing constructs and cloned into pEF5-FRT. pEF5-FRT constructs to prepare stable IMCD3 Flp-in cell lines contained the following elements: (1) mouse SMO (residues 33–794) with an N-terminal HA signal sequence, FLAG epitope, and 3C protease site; (2) an internal ribosome entry site (IRES); and (3) either PKA-Cα, NbSmo2, or Nbβ2AR80, fused to the N-terminus of mNeonGreen. All constructs were built using Gibson Assembly and verified by Sanger sequencing. For experiments involving protein purification, BacMam viruses were prepared as previously described [90]. RNA was transcribed in vitro using mMESSAGE Machine to generate capped RNA for zebrafish injections.

### Modification of endogenous *Smo* locus via CRISPR/Cas9

The endogenous *Smo* locus in IMCD3 Flp-in parental cells was appended with a HiBiT sequence using the Alt-R system (IDT). Briefly, Cas9 RNP complexes were assembled in vitro using purified Cas9, a crRNA targeting the 3′ end of the *Smo* coding sequence (“Mm.HC9.BZDB9357.AA”), a custom-designed ssODN corresponding to HiBiT preceded by a 6 amino acid linker (“GGTGGCaGCGGAGGGaGTGTGAGCGGCTGGCGGCTGTTCAAGAAGATTAGCTAA”), Alt-R HDR enhancer, and tracrRNA (all from IDT, assembled according to manufacturer’s instructions). Cas9 RNP was electroporated into IMCD3 Flp-in parental cells (Neon electroporation system, Thermo Fisher Scientific). Cells were then single-cell sorted via FACS, allowed to recover, and individual clones tested for HiBiT insertion using a Nano-Glo HiBiT Lytic Detection System. Positive cells were expanded, and HiBiT insertion at the *Smo* locus confirmed via PCR analysis of genomic DNA along with HiBiT blotting. Genomic DNA was extracted using a Blood and Cell Culture DNA mini kit (Qiagen) and PCR products amplified with primers TTCCCGCACTAACCTAATGG (fwd) and GAGGCCTACCAATCGCTGTA (rev).

### Generation of GRK-deficient cells via CRISPR/Cas9

Mutations in the GRK genes were introduced by a CRISPR/Cas9 system as described previously [43] with minor modifications. Briefly, an sgRNA-encoding sequence targeting GRK2 (5′-CGAGGTCTATGGGTGCCGGA-3′), GRK3 (5′-TTATTGGACGAGGAGGATTC-3′), GRK5 (5′-AATGTATGCCTGCAAGCGCTTGG-3′), or GRK6 (5′-GTGCTACTCAAGGCCCGGGAAGG-3′) was inserted into the BbsI site of the pSpCas9 (BB)-2A-GFP (PX458) vector (a gift from Feng Zhang; 42230; Addgene). HEK293A cells (Thermo Fisher Scientific) were transfected with the sgRNA-inserted PX458 plasmid vectors, and GFP-positive cells were isolated by a FACS. The sgRNA/Cas9/GFP-expressing cells were cultured in a 96-well plate, and clonal cells were isolated by a limiting dilution method. The clones were screened for their genotype by a restriction enzyme digestion method. The GRK-targeted loci were PCR-amplified by using following pairs of primers: 5′-GTAAATATGTGGCAAGGATGGC-3′ and 5′-TCCCCGAGGTATCCCACC-3′ (GRK2); 5′-TTGTGTTTGGATTTCCCAGTTGAC-3′ and 5′-GCCTACAGCTTATTTCTTTTGGAGG-3′ (GRK3); 5′-TCTGACCCCATCCATTCTCTAC-3′ and 5′-GATGCTCACTCACCACAAACTG-3′ (GRK5); 5′-GAGAACATCGTAGCGAACACG-3′ and 5′-AGGTGCGGAGGAGGAAGAC-3′ (GRK6). The resulting PCR fragment was digested with following restriction enzymes: Hap II (GRK2), Hinf I (GRK3), Hha I (GRK5), and Sma I (GRK6), which were located within or adjacent to the Cas9-mediated double-strand break site. Candidate clones with resistance to restriction enzyme were subjected to Sanger sequencing and correctly mutated clones were used in the study. The detailed characterization of the GRK-deficient cells will be described elsewhere.

### GLI-luciferase assays

GLI-luciferase assays were performed as previously described [42,133]. Briefly, cells were seeded into 24-well plates and transfected (TransIT 2020) at 250 ng DNA per well as follows: (1) 30:1 mixture of 8xGli-Firefly and SV40-*Renilla* plasmids (50%); (2) SMO expression constructs (2% to 10%); and (3) GFP to bring the total to 100%. Transfected cells were cultured to confluency, shifted to low-serum medium (0.5% FBS), and stimulated for 48 to 60 hours with 1:20 dilutions of control or ShhN-containing conditioned medium, collected from stably transfected HEK293-ShhN cells as previously described [42,133]. Luminescence was determined using a Dual Luciferase Assay Kit (Promega) on a Berthold Centro XS3 luminometer with automated injection. The ratio of Firefly to *Renilla* luciferase (“GLI-Luc Activity”) is reported as relative luciferase units (RLUs).

### CREB-based assays for PKA signaling

HEK293FT wild-type or G⍺-null cells were seeded into a 24-well plate and transiently transfected (TransIT-293 or Lipofectamine 2000) with a 20% (w/w) 30:1 ratio of pGL4.29[*luc2p*/CRE/Hygro] and constitutively expressing SV40-*Renilla* plasmids. Cells were cotransfected with 0.625% (w/w) PKA-C (pCMVSPORT6) and 24% (w/w) SMO (or SMO674) or M2AchR (pCDNA3.1+). Transfection mixtures were supplemented with a GFP plasmid to normalize the amount of DNA delivered to each well (250 ng/well). For experiments in parental HEK293 versus ΔGRK cells, transfesction mixtures contained 1% PKA-C, 24% SMO, 20% GRK2, and GFP to bring the total amount of DNA to 250 ng/well. Two days post-transfection, cells were incubated with the indicated drug treatments, diluted in media, for 4 hours at 37°C. For experiments involving ShhN treatment, concentrated control- or ShhN-conditioned medium was diluted 1:4 prior to addition to cells. Media was removed, and cells were washed once with PBS and analyzed using a Dual Luciferase Assay Kit. After lysing cells in 100 μl of passive lysis buffer (PLB) per well and incubating for 15 minutes at room temperature on a platform rocker, the lysate was diluted 1:10 in PLB, and 10 μL was transferred to an opaque 96-well assay plate. Luminescence was determined using a Dual Luciferase Assay Kit (Promega) on a Berthold Centro XS3 luminometer with automated injection. The normalized ratio of Firefly to *Renilla* (CREB Reporter Activity (% Max.)) is reported. For all CREB assays, data represent mean ± SEM from triplicate wells, and data are representative of at least 2 independent experiments. For analysis of CREB protein phosphorylation by western blot, transfection mixtures contained 2.4% PKA-C, 24% SMO, and GFP to bring the total amount of DNA to 250 ng.

#### Confocal microscopy in HEK293 cells

Imaging of transfected HEK293FT cells was performed as previously described [90]. Briefly, cells were transiently transfected with the indicated FLAG-tagged SMO constructs and GFP or YFP-tagged Nb or PKA-C constructs, typically at a 10:1 ratio of SMO to Nb/PKA-C, in 35-mm poly-D-lysine-coated glass-bottom dishes, with 3 μg total DNA per transfection. In experiments involving drug treatments, cells were stimulated with various pharmacological agents overnight, as indicated in each figure. Live cells were subsequently stained for 5 min with an Alexa647-conjugated M1 anti-FLAG antibody (1:2,000) followed by washing in HBSS, mounting, and visualization. Images were acquired on a Leica SP8 laser scanning confocal microscope. Line scan analysis was performed using ImageJ software. Within each experiment, all images were acquired with identical gain and exposure settings and processed identically in ImageJ. For quantitative determination of colocalization, membrane fluorescence for raw images was measured using ImageJ software. Measurements for red and green channels were taken by drawing 4 small lines (width of 4 pixels) along the membrane of the cells at different locations around the entire cell. The mean fluorescence intensities (from the 4 line measurements) for each cell were averaged. Additionally, in **Fig 2**, the mean cytoplasmic signal was subtracted from this value to obtain the mean fluorescence intensity specifically at the membrane. Then, the mean green fluorescent intensity for each cell was divided by the mean red fluorescent intensity and graphed.

### Immunoblotting

For western blotting, cells were lysed for 1 hour at 4°C in RIPA buffer (150 mM NaCl, 1% NP-40, 0.5% Na deoxycholate, 0.1% SDS, 50 mM Tris, pH 8.0). Following clarification (20,000 x g, 4°C, 30 minutes), supernatants were separated by SDS-PAGE (Criterion Stain Free gels), transferred to PVDF membranes, blocked in 5% milk, and probed with the relevant primary and secondary antibodies, followed by chemiluminescent detection (ChemiDoc, Bio-Rad). For HiBiT Western, lysate in RIPA buffer were separated by SDS-PAGE, transferred to nitrocellulose, and probed for HiBiT according to the manufacturer’s instructions. Quantification of western blot band intensity was performed in ImageLab software (Bio-Rad). In the anti-PKA-C blot in **Fig 3C**, the band intensity for the (transfected) PKA-C-YFP was divided by the band intensity for endogenous PKA-C, and the ratio was reported as “% over endogenous.” Because BRET signals only arise from transfected cells, whereas the PKA-C-YFP Western signal reports the expression levels for all cells in the population, the PKA-C-YFP band intensity underestimates the total amount of PKA-C-YFP in transfected cells. To correct for this, band intensity was divided by IMCD3 transfection efficiency (38%, measured in separate experiments via FACS) before calculating the “% over endogenous” ratio.

#### Confocal microscopy in IMCD3 cells and *Smo*^−/−^ MEFs

Live IMCD3 stable Flp-in cell lines (SMO + PKA-C, SMO + NbSmo2, SMO + Nbβ2AR80) were imaged together on the same day to minimize any possible day-to-day variations in the experiments that could confound comparisons. The indicated IMCD3 stable Flp-in cells were grown to confluency on 8-well μslide chamber slides (Ibidi), then shifted to low-serum medium (DMEM/F12 + 0.5% FBS + pen-strep) + 1 μM SAG21k to induce ciliogenesis and stimulate SMO translocation to cilia. On the day of imaging, cells were stained for 5 to 10 minutes in Hanks’ balanced saline solution (HBSS) containing M1 FLAG-647 conjugate (1:1,000) and Hoechst counterstain (5 μg/ml), followed by 3 brief washes in HBSS. Cells were overlaid with HBSS containing 1 μM SAG21k and imaged immediately on a Leica SP8 laser scanning confocal microscope, using a 40× water immersion lens. All images were acquired as Z-stacks in sequential frame mode to minimize any potential crosstalk between channels using the identical exposures, gain settings, and zoom factors. Three-dimensional reconstructions of Z-stacks were performed in Fiji using the 3D Viewer plugin. Quantification of SMO (red) and PKA-C/NbSmo2/Nbβ2AR80 staining was performed using CiliaQ [134] and reported as a ratio of the green fluorescence in each cilium, normalized to the red fluorescence. For fixed-cell imaging of SMO wild-type and Ala mutants (**S11B Fig**), *Smo*^−/−^ MEFs were transfected on coverslips with the indicated plasmids (N-terminally tdTomato-tagged SMO657 or SMO657-Ala), grown to confluency, shifted to DMEM + 0.5% FBS + SAG21k overnight, then fixed, permeabilized, and stained as previously described, using an anti-Arl13b antibody to mark cilia. Z-stacks were acquired on a Leica SP8 in sequential frame mode using the identical exposures, gain settings, and zoom factors for each sample. The total SMO fluorescence in cilia was quantified manually, as described previously [31]. For fixed-cell imaging of SMO-Nb fusions stably expressed in *Smo*^−/−^ MEFs (**S11A Fig**), cells were fixed, permeabilized, and stained with anti-RFP and anti-Arl13b antibodies, then imaged on a Nikon Ti inverted epifluorescence microscope using a 60× oil immersion lens.

#### BRET assays

BRET assays were performed in cultured HEK293FT cells transiently transfected with 1.2 μg of DNA comprised of nanoluc-tagged SMO donors (or other plasmids as noted in the text) and YFP-tagged acceptors. We typically transfected a 1:3 (w/w) ratio of donor to acceptor, but we decreased the amount of acceptor DNA (0.3 μg SMO and 0.03 μg to 0.1 μg of PKA-C) in experiments where avoiding saturation of BRET donor was critical (**Figs 5A and 5D, 6D and 7B**). Saturation studies with PKA-C-YFP and PKA-R-YFP BRET acceptors employed 0.03 to 0.9 μg of each plasmid. A non-expressing vector (pV1392) was used to equalize the amount of DNA in each transfection mixture. We used N-terminal YFP fusions of PKA-C for a number of initial experiments (**Fig 4B and 4C**), but ultimately switched to a carboxyl-terminally tagged PKA-C which exhibited stronger BRET responses with SMO and minimized any potential disruptions of an N-terminal tag on posttranslational modifications occurring on the N-tail of PKA-C. BRET assays in IMCD3 cells were conducted similarly, except that Lipofectamine 2000 or TransIT 2020 were used for transfection, and for BRET studies involving HiBiT-tagged endogenous SMO, 0.9 μg of LgBiT/pEF5-FRT (which exhibits no luminescence unless HiBiT is present [135]) was included in all transfections. Transfected cells were cultured for an additional 1 to 2 days, trypsinized, and aliquoted into white, opaque, poly-d-lysine treated 96-well plate at 1.5 × 10^5^ cells/ml and allowed to adhere overnight. For IMCD3 BRET studies, cells were incubated during this period with SAG21k, to induce maximally efficient formation of SMO/PKA-C complexes. On the day of each BRET measurement, we replaced culture medium with HBSS containing the indicated pharmacological agents or vehicle controls (if applicable). Following replacement of culture medium and drug treatment (1 hour for SMO modulators and 4 hours for GRK2/3 inhibitor), we measured the raw YFP fluorescence (485 nm excitation, 535 nm emission, and +/− 20 nm bandwidth) for each condition (Tecan Spark plate reader). Nanoluc substrate (5 μM Coelenterazine h for HEK293 cells and 10 μM furimazine for IMCD3 cells) was subsequently added, and the cells were incubated for 15 to 30 minutes at room temperature before measurement of nanoluc (360 to 425 nm, 1-second integration time) and YFP (535 to 590 nm, 1-second integration time) emission signals. Data are reported as BRET units, which represents the YFP/nanoluc ratio. Background signals were defined using cells expressing nanoluc donor alone and were subtracted from all measurements.

#### Nb selections, FACS, and FSEC assays

NbSmo2 was isolated via MACS and FACS sorting from yeast display libraries using fluorescently labeled SMO-agonist and SMO-inverse agonist complexes, as previously described [90]. FACS analysis, Nb expression in *Escherichia coli*, fluorescent labeling of purified Nb, and fluorescence detection size exclusion chromatography (FSEC) were all performed as previously described [90].

#### Purification of SMO from mammalian cells

We followed similar procedures for SMO purification in all experiments. We provide a basic protocol in (1) and describe modifications to this protocol in (2) and (3):

1. **Determination of SMO/Nb complex formation:** Isolation of FLAG-tagged SMO from HEK293-Freestyle cells was essentially performed as previously described [90], with minor modifications. Briefly, high-titer BacMam viruses encoding FLAG-tagged SMO566-Gα_o_ constructs were infected into 3 ml of HEK293-Freestyle cells (at 1.5 to 3 × 10^6^ cells/ml), alone, or with viruses encoding GFP-tagged NbSmo2, NbSmo8, or Nbβ2AR80, all at 15 μl virus/ml HEK293 cells, along with 10 mM sodium butyrate to increase expression efficiency. After 24 hours, cells were washed once in HBSS, treated with the indicated drugs (or DMSO vehicle) for 70 minutes, and snap-frozen in liquid nitrogen. Cell pellets were solubilized in 0.3 ml buffer A (20 mM HEPES pH 7.5, 150 mM NaCl, 0.1 mM TCEP, 0.5% LMNG/0.05% CHS), 1 mM CaCl_2_•6H_2_O, and protease inhibitors (Thermo Fisher Scientific, Cat#A32955) for 1 hour at 4°C with rotation. Lysates were clarified by centrifugation at 20,000 x g, 30 minutes at 4°C. Supernatants were incubated with 10 μl of pre-equilibrated M1 FLAG affinity resin for 1 hour at 4°C with rotation. Resin was washed 3 times in buffer B (20 mM HEPES pH 7.5, 150 mM NaCl, 0.1 mM TCEP, 0.05% LMNG/0.005% CHS, 1 mM CaCl_2_•6H_2_O) and eluted by incubation for 5 minutes at room temperature in 30 μl of the same buffer + 5 mM EDTA and 0.2 mg/ml FLAG peptide. Removal of the final wash buffer and the elution step were performed with a Hamilton syringe (22s gauge, blunt tip). Nb copurification in each elution was determined via GFP quantification in a Tecan Spark plate reader and normalized to the amount of Nb-GFP in the corresponding total cell lysate. Samples were also analyzed by SDS-PAGE followed by total protein staining (StainFree, Bio-Rad) and in-gel GFP fluorescence on a Bio-Rad ChemiDoc XRS+ imaging station, to estimate purity and confirm that Nb-GFP fusions were not proteolytically degraded.
2. **Analysis of SMO phosphorylation:** We followed a similar procedure as for (1), with the following modifications: FLAG-tagged SMO674, SMO674Ala, or SMO566 (20 μl/ml) were coinfected with GRK2-GFP (10 μl/ml), and drugs were added for 4 hours prior to washing in PBS and harvesting. Cells were solubilized in 0.6 ml buffer C (50 mM HEPES pH 7.5, 200 mM NaCl, 1% DDM/0.1% CHS, 0.1 mM TCEP, 1 mM CaCl_2_•6H_2_O + protease/phosphatase inhibitors). M1 FLAG affinity resin was washed 3 times in buffer D (50 mM HEPES pH 7.5, 200 mM NaCl, 0.1% DDM/0.01% CHS, 0.1 mM TCEP, 1 mM CaCl_2_•6H_2_O) and eluted in 100 μl of the same buffer + 5 mM EDTA and 0.2 mg/ml FLAG peptide. A total of 10 μl of each sample was mixed with 2x SDS-PAGE sample buffer (Bio-Rad) and run on duplicate SDS-PAGE gels (Stain Free TGX 7.5%). One of these gels was used for total protein quantification (Bio-Rad Stain Free), and the other was stained with ProQ Diamond phosphoprotein stain following the manufacturer’s instructions. The remaining 90 μl of the sample was snap-frozen and stored at −80°C for MS.
3. **Determination of SMO/PKA-C complex formation:** We followed a similar procedure as for (1), with the following modifications: FLAG-tagged SMO674, SMO566, SMO657-NbSmo2, or SMO657-Nbβ2AR80 were coinfected with YFP-tagged PKA-C or Nbβ2AR80 BacMam viruses (20 μl/ml for each). Cells were harvested 48 hours postinfection. In experiments involving DSP crosslinker, cells were washed twice in PBS, resuspended in 5 ml PBS, and DSP crosslinker (freshly prepared in anhydrous DMSO) was diluted from a 20 mM stock to achieve the indicated final concentration. Cells were incubated at room temperature for 30 minutes with rotation, and Tris pH 7.5 was added to a final concentration of 20 mM to quench any remaining unreacted crosslinker. Cells were washed once with PBS, snap-frozen in liquid nitrogen, and stored at −80°C. Cell pellets were solubilized in low-salt solubilization buffer (20 mM HEPES pH 7.5, 150 mM NaCl, 0.1 mM TCEP, 0.5% GDN, 1 mM CaCl_2_•6H_2_O), washed in low-salt wash buffer (20 mM HEPES pH 7.5, 150 mM NaCl, 0.1 mM TCEP, 0.05% GDN, 1 mM CaCl_2_•6H_2_O), and eluted in 40 μl of the same buffer with 5 mM EDTA and 0.2 mg/ml FLAG peptide. Quantification of YFP in FLAG eluates was performed on a Tecan Spark fluorescence plate reader. SDS-PAGE was also performed using 4% to 20% Stain Free TGX gels, with 2.5 μl of cell lysate or 7.5 μl of FLAG eluate loaded per lane. Identity of YFP-tagged PKA-C in StainFree imaging was confirmed by overlap with an aligned YFP image of the corresponding gel. SMO expression was assessed in input fractions (whole cell lysates) via transfer to PVDF, followed by blocking and incubation with M1 FLAG primary antibody and anti-mouse secondary antibody as described above.

#### Protein digestion for mass spectrometry

The purified protein extracts from different conditions were denatured and reduced in 1.7 M urea, 50 mM Tris-HCl pH 8.0, 1 mM DTT at 37°C for 30 minutes, alkylated in the dark with 3 mM iodoacetamide at room temperature for 45 minutes, and excess iodoacetamide was quenched with 3 mM DTT for 10 minutes at room temperature. For digestion, proteins were incubated with (1) 0.5 μg trypsin at 37°C overnight; (2) 1 μg chymotrypsin at 37°C overnight; or (3) consecutively with 1 μg chymotrypsin followed by 0.5 μg trypsin both at 37°C overnight. To stop the digestion, samples were acidified with 0.5% trifluoroacetic acid (TFA). Digested samples were desalted for MS analysis using a BioPureSPE Mini 96-Well Plate (20 mg PROTO 300 C18; The Nest Group) according to standard protocols.

#### Global mass spectrometry data acquisition and analysis

Samples were resuspended in 4% formic acid/2% acetonitrile solution and analyzed on an Q-Exactive Plus MS system (Thermo Fisher Scientific) equipped with an Easy nLC 1200 ultra-high pressure liquid chromatography system (Thermo Fisher Scientific) interfaced via a Nanospray Flex nanoelectrospray source. Samples were loaded onto a 75 μm ID C18 reverse phase column packed with 25 cm ReprosilPur 1.9 μm, 120Å particles (Dr. Maisch). Mobile phase A consisted of 0.1% FA, and mobile phase B consisted of 0.1% FA/80% ACN. Peptides were separated by an organic gradient ranging from 4.5% to 32% acetonitrile over 53 minutes, then held at 90% B for 9 minutes at a flow rate of 300 nl/min delivered by an Easy1200 nLC system (Thermo Fisher Scientific). All MS spectra were collected with orbitrap detection, while the 20 most abundant ions were fragmented by HCD and detected in the orbitrap. All MS1 spectra were collected with orbitrap detection at a 70,000 resolution and a scan range from 300 to 1,500 m/z, while the 20 most abundant ions were fragmented by HCD and detected at a resolution of 17,500 in the orbitrap; data were acquired using the Thermo software Xcalibur (4.2.47) and Tune (2.11 QF1 Build 3006). For all acquisitions, QCloud was used to control instrument longitudinal performance during the project [136]. All proteomic data were searched against the human UniProt database (UniProt reviewed sequences downloaded 07/2018) augmented with the sequence of the affinity tagged SMO. Peptide and protein identification searches, as well as label-free quantitation, were performed using the MaxQuant data analysis algorithm (version 1.6.3.3) [137]. Variable modifications were allowed for methionine oxidation, phosphorylation on serine, threonine, and tyrosine, and protein N-terminus acetylation. A fixed modification was indicated for cysteine carbamidomethylation. Full trypsin and/or chymotrypsin specificity was required. The first search was performed with a mass accuracy of +/− 20 parts per million (ppm), and the main search was performed with a mass accuracy of +/− 4.5 ppm. A maximum of 5 modifications were allowed per peptide. A maximum of 2 missed cleavages were allowed. The maximum charge allowed was 7+. Individual peptide mass tolerances were allowed. For MS/MS matching, a mass tolerance of +/− 20 ppm was allowed, and the top 12 peaks per 100 Da were analyzed. MS/MS matching was allowed for higher charge states, water, and ammonia loss events. The data were filtered to obtain a peptide, protein, and site-level false discovery rate of 0.01. The minimum peptide length was 8 amino acids. All peptide and protein identifications were filtered to a 1% false discovery rate. Match between run was activated, and identifications matched in a window of 0.7 minutes with an alignment time window of 20 minutes. Statistical analysis of unmodified SMO and detected phosphosites was performed separately for the different digestion conditions using the statistical framework MSstats [138]. Intensities are estimated using the sample quantification function in MSstats which provides model-based estimation of phosphosite and protein abundance combining individual peptide intensities. Quantification was graphed using Prism 8 (GraphPad). The raw data have been deposited to the ProteomeXchange Consortium via the PRIDE partner repository [139] (https://www.ebi.ac.uk/pride/archive/) with the dataset identifier PXD019346. SMO snake plot (**S10 Fig**) was generated using Protter [140].

#### Targeted mass spectrometry data acquisition and analysis

Digested samples were analyzed on an Orbitrap Exploris 480 MS system (Thermo Fisher Scientific) equipped with an Easy nLC 1200 ultra-high pressure liquid chromatography system (Thermo Fisher Scientific) interfaced via a Nanospray Flex nanoelectrospray source. For all analyses, samples were loaded onto a 75 μm ID C18 reverse phase column packed with 25 cm ReprosilPur 1.9 μm, 120Å particles (Dr. Maisch). Mobile phase A consisted of 0.1% FA, and mobile phase B consisted of 0.1% FA/80% ACN. Peptides were separated by an organic gradient from 2% to 28% mobile phase B over 32 minutes followed by an increase to 44% B over 19 minutes, then held at 90% B for 9 minutes at a flow rate of 300 nL/minute. Analytical columns were equilibrated with 6 μL of mobile phase A. To build a spectral library, the 4 biological replicates for each condition were pooled and acquired in a data-dependent manner. Data dependent analysis (DDA) was performed by acquiring a full MS1 scan over a m/z range of 350 to 1,250 in the Orbitrap at 120,000 resolving power (@200 m/z) with a normalized AGC target of 100%, an RF lens setting of 40%, and a maximum ion injection time set to “Auto.” Dynamic exclusion set to 30 seconds, with a 10 ppm exclusion width setting. Peptides with charge states 2 to 6 were selected for MS/MS interrogation using higher energy collisional dissociation (HCD), with a set cycle time of 1 second. MS/MS scans were analyzed in the Orbitrap using isolation width of 1.3 m/z, normalized HCD collision energy of 30%, normalized AGC of 200% at a resolving power of 15,000, and with a maximum ion injection time set to “Auto.” For all acquisitions, QCloud was used to control instrument longitudinal performance during the project [136]. All proteomic data were searched against the human UniProt database (UniProt reviewed sequences downloaded 07/2018) augmented with the sequence of the affinity tagged SMO. Peptide and protein identification searches, as well as label-free quantitation were performed using the MaxQuant data analysis algorithm (version 1.6.12.0) [137] using above described parameters. The database search results were used to generate a spectral library in Skyline (version 20.2.0.343) [141] and to extract optimal coordinates for targeted proteomics assays (so called parallel reaction monitoring (PRM) assays). PRM measurements were performed on all 4 biological replicates per condition separately using the above described gradient for spectral library generation but operating the Orbitrap Exploris 480 in PRM mode. Targeted MS2 spectra were acquired using the following parameters: 60 k resolution, scan range set to “Auto,” HCD with 30% NCE, RF lens setting of 50%, an AGC target set to “Standard,” the maximum injection time set to “Dynamic,” desired minimum points across the peak set to “9”, and an isolation window of 1.2 m/z. Selected SMO peptides were targeted in 3-minute wide transition windows. The resulting data were analyzed with Skyline (version 20.2.0.343) for identification and quantification of peptides [141]. MSstats was used for statistical analysis [138]. Statistical analysis of unmodified SMO and detected phosphosites was performed separately for the different digestion conditions using the statistical framework MSstats [138]. Intensities are estimated using the sample quantification function in MSstats which provides model-based estimation of phosphosite and protein abundance combining individual peptide intensities. Quantification was graphed using Prism 8 (GraphPad). Raw data and PRM transition files can be accessed, queried, and downloaded via Panorama (https://panoramaweb.org/) [142]). For access please use the following link: https://panoramaweb.org/Panorama%20Public/2021/UCSF%20Krogan%20Lab%20-%20Smo_phospho/project-begin.view?

#### Sequence alignment

Alignment of SMO sequences from *M*. *musculus* (mouse, NP_795970.3), *Homo sapiens* (human, NP_005622.1), *Gallus gallus* (chicken, P_015143674.1), *Xenopus laevis* (frog, NP_001128704.1), *Danio rerio* (zebrafish, NP_571102.2), *Ciona intestinalis* (vase tunicate, NP_001071819.1), and *Drosophila melanogaster* (fruit fly, NP_523443.1) was performed using Clustal Omega [143].

### Zebrafish maintenance, RNA microinjection, and muscle histology

Zebrafish *D*. *rerio* were maintained in accordance with approved institutional protocols at the University of Utah. Specifically, all experimental protocols were approved by the University of Utah Institutional Animal Care and Use Committee and were in accordance with the guidelines from the National Institutes of Health (Approval number: 19–11006). Adult zebrafish were maintained under standard conditions [144] and kept on a light–dark cycle of 14 hours in light and 10 hours in dark at 27°C. Zebrafish carrying the smo^hi1640^ [145] null mutations were obtained from Sarah Lusk and Kristen Kwan, University of Utah. Embryos from natural spawnings were generated and collected as described [144]. Live embryos were maintained at 28°C. Developmental staging was based on [146]. Prior to mating, adult zebrafish were genotyped by biopsy of the 2 mm most distal portion of the tail fin. Fin DNA was extracted in 100 μL of 50 mM NaOH, boiled at 95°C for 20 minutes and neutralized with 10 μL of Tris-HCl (pH 7.5). Fish were genotyped with the following primers: smo-140c (gaaggcttcctcttgagtttctgag), smo-5UTR (aactcaacgcgcatcgcgac), smo-wnt1 (cagttctcacgtctgctacttgca), smo-wnt2 (acttccggcgtgttggagaattc), and smo-Nltr3 (ctgttccatctgttcctgac). Smo-140c and smo-Nltr3 amplify a 200 base pair product from the smohi1640 mutant insertion. Smo-wnt1 and smo-wnt2 amplify a 400 base pair control band from a non-smo locus. Smo-Nltr3 and smo-5UTR amplify a PCR product from the smo wild-type locus.

For RNA injection, 0.1 ng (SMO, SMO-NbSmo2, or SMO-Nb*β*2AR80) or 0.2 ng (SMO657 or SMO657Ala) of RNA was injected into the cytoplasm of 1-cell stage embryos.

For immunohistochemistry, embryos were fixed at 26 hours postfertilization (hpf) with fresh 4% paraformaldehyde in PBS at room temperature for 2 hours. Fixed embryos were dehydrated in methanol and stored at −20°C until processing for immunohistochemistry according to standard procedures [144]. In brief, embryos were rehydrated into PTw (PBS with 0.1% Tween-20), incubated 7 minutes in acetone at −20°C, washed in water, then PTw, and then incubated in blocking agent (10% heat-inactivated sheep serum, 2 mg/mL BSA, 1% DMSO, 0.1% Triton X-100 in PBS) for at least 1 hour at room temperature. Embryos were incubated in primary antibodies diluted in blocking agent overnight at 4°C. Primary antibodies were removed, and embryos were washed extensively with PBDT (2 mg/mL BSA, 1% DMSO, 0.1% Triton X-100 in PBS). Embryos were next incubated with appropriate secondary antibodies in the dark overnight at 4°C followed by extensive washes in PBDT. Primary antibodies used were 4D9 at 1:5 (anti-En; Monoclonal Antibody Facility, Institute of Neuroscience, University of Oregon) and Prox1 at 1:1,000 (Prox1; AngioBio). Goat anti-rabbit IgG-594 (Jackson Laboratory) was used at 1:500 as a secondary antibody to detect Prox-1 staining. Goat anti-mouse IgG-HRP (Jackson Laboratory) was used at 1:250, followed by tyramide amplification (Thermo Fisher Scientific TSA-488 amplification kit) to detect 4D9 staining. Embryos were taken stepwise through a glycerol series into 75% glycerol. Heads and yolks were removed, and trunks were mounted prior to image acquisition. For embryos, removed heads were used for genotyping (as with adult fins), except embryonic head DNA was extracted in 30 μL 50 mM NaOH and neutralized in 3 uL Tris-HCl (pH 7.5). Confocal imaging was performed with a Nikon A1 inverted laser scanning confocal microscope. Image processing and quantification was completed using Fiji. Confocal images represent max projections of Z-stacks taken 5 μm apart for a total of approximately 20 μm (lateral views). Images used are of somites 11 through 15. Brightness and contrast were adjusted linearly where appropriate. In all images, anterior is to the left. Sample sizes (number of injected embryos from smo^−/+^ incross) are as indicated.

## Supporting information

S1 Fig SMO expression constructs and controls for CREB reporter–based PKA activity assay. Related to **Fig 1**. (**A**) Jalview conservation score (0–10) and DISOPRED disorder score (0–1, with values >0.5 indicative of disorder) are plotted on the y-axes, while SMO amino acid numbering is plotted on the x-axis. Locations of pCT (mustard) and dCT (lavender) are indicated below the graphs. (**B**) Table of SMO constructs used in this study. Note that CRD (red) and 7TM (blue) domains are not drawn to scale. We used SMO657, truncated immediately after the pCT, for many of our cell-based experiments, based on its ability to recapitulate >70% of the activity of wild-type SMO in GLI reporter assays (see (C)). However, secondary structure predictions revealed a conserved region between amino acids 657–674, predicted to be ordered. Because these parameters might help to increase the biochemical stability of SMO, we extended the construct boundary (SMO674) in any experiments requiring purification of SMO protein. (**C**) *Smo*^−/−^ MEFs were transfected with GLI-luciferase reporter plasmid, along with a GFP negative control (“Neg.”), full-length SMO, or truncation mutants lacking the dCT (SMO657) or pCT (SMOΔ561–657). Following transfection, cells were stimulated with conditioned medium containing the N-terminal signaling domain of Sonic hedgehog (ShhN, green) or control, non-ShhN-containing conditioned medium (Vehicle, black). (**D**) FLAG-tagged full-length SMO or SMO674 were expressed in HEK293S GnTI- cells, stimulated with the SMO agonist SAG21k or the SMO inverse agonist KAADcyc for 4 hours (to assess possible effects of these compounds on SMO expression levels), then solubilized in detergent and purified over M1 FLAG resin. Elution was analyzed by SDS-PAGE and total protein staining (StainFree imaging). Note that SMO tends to migrate slightly faster than expected in these experiments, likely due to a reduction in glycosylation in GnTI- cells compared to their wild-type counterparts; (**E**) HEK293 cells were transfected with the indicated plasmids, treated with vehicle or ShhN for 4 hours, then subject to CREB reporter assays. Note that ShhN does not fully reverse the effect of PTCH1, likely because (1) PTCH1 is being expressed at high levels via transient transfection from a strong constitutive promoter; and (2) the ShhN in this experiment lacks a cholesterol modification [132]; nevertheless, the effect of ShhN is highly specific, as it only occurs in cells expressing PTCH1. (**F**) Wild-type (left) or Gα-null (right) HEK293 cells were subject to CREB reporter assays as described in **Fig 1B** and **1D**. While SMO blocked effects of PKA-C less efficiently than in **Fig 1B** and **1D**, KAADcyc more fully reversed the effect of SMO expression. (**G**) Wild-type (left) or Gα-null (right) HEK293 cells were transfected with GFP (negative control) or M2AchR expression plasmids and stimulated with forskolin (black, 500 nM for wild-type cells, 80 μM for Gα-null cells) in the presence or absence of carbachol (gray, 3 μM). Note that substantially less forskolin is needed to induce cAMP signals in wild-type HEK293 cells compared to Gα-null cells due to the presence in the former of Gα_s_, which binds to and sensitizes AC to forskolin treatment (personal communication, A. Inoue). In addition, basal reporter activity in Gα-null cells is higher following M2AchR expression, but the interpretation of this effect is uncertain because it is not altered by treatment with carbachol. Data are normalized to 100%, which reflects reporter activation from PKA-C-transfected cells treated with vehicle (*n* = 3 biological replicates per condition, error bars represent SEM). The underlying data for this figure can be found under **S1 Data** and uncropped protein gels are included in **S8 Data**. See **S1 Table** for statistical analysis. 7TM, seven-transmembrane; AC, adenylyl cyclase; CREB, cyclic AMP response element binding protein; dCT, distal segment of the cytoplasmic tail; GLI, glioma-associated; KAADcyc, KAAD-cyclopamine; M2AchR, M2 acetylcholine receptor; MEF, mouse embryonic fibroblast; pCT, proximal segment of the cytoplasmic tail; PKA, protein kinase A; PKA-C, PKA catalytic subunits; PTCH1, Patched1; RLU, relative luciferase unit; ShhN, N-terminal signaling domain of Sonic hedgehog; SMO, Smoothened.
(PDF)

S2 Fig Controls for confocal imaging of HEK293 cells and outline of Nb2 selections. Related to **Fig 2**. (**A**) Representative image of PKA-C localization in HEK293 cells not expressing SMO. (**B**) Binding of NbSmo2, displayed on the surface of yeast [90], to purified, detergent-solubilized SMO-agonist (SAG21k) complexes or SMO-inverse agonist (KAADcyc) complexes in solution, was assessed by flow cytometry. Note that this experiment used SMO566, which lacks the entire cytoplasmic tail. (**C**) FLAG-tagged SMO566-Gα_o_ was expressed in HEK293 cells alone or with GFP-tagged NbSmo2, NbSmo8, or Nbβ2AR80. Following treatment with SMO agonist (SAG21k, 1 μM), inverse agonist (KAADcyc, 1 μM), or MBCD (8 mM, which extracts SMO sterol agonists from membranes [42]), SMO-Nb complexes were isolated from detergent-solubilized cells via FLAG affinity chromatography and Nb levels measured via GFP fluorescence quantification. Ratios of GFP fluorescence in FLAG eluates, normalized to GFP fluorescence in each lysate before affinity chromatography, are reported. (**D**) NbSmo8-GFP colocalization with SMO566-NbSmo2 fusion at the cell membrane. The presence of NbSmo2 is predicted to prevent binding of NbSmo8 to SMO if the Nbs bind to overlapping epitopes. SMO566-Gα_o_ serves as a positive control. Line scan analysis is shown to the right of each merged image, with a dotted line indicating the location of the scan. (**E**) In vitro binding of Alexa647-labeled NbSmo8 to SMO566 in the presence of non-fluorescent NbSmo2 competitor, as assessed by FSEC. Non-fluorescent NbSmo8 or NbMOR39 (which binds a non-SMO GPCR [147]) serve as positive and negative controls for NbSmo8 competition binding, respectively. FSEC, fluorescence detection size exclusion chromatography; GPCR, G protein–coupled receptor; KAADcyc, KAAD-cyclopamine; MBCD, methyl-β-cyclodextrin; Nb, nanobody; PKA-C, PKA catalytic subunits; SMO, Smoothened.
(PDF)

S3 Fig Additional controls for microscopy experiments. Related to **Fig 2.** (**A**) Line scans for colocalization images in **Fig 2A** and **2B**. Colors are the same as described in the main figure panels. Dotted line indicates location of the scan. (**B**) Surface expression of SMO674 and SMO566 in HEK293 cells was assessed via FACS staining of nonpermeabilized cells with an FLAG-Alexa 647 conjugate. HEK293 cells not expressing SMO serve as a negative control (“CTRL”). (**C**) Raw data (3D reconstruction) of stable IMCD3 cells coexpressing FLAG-tagged SMO and mNeonGreen-tagged NbSmo2 or Nbβ2AR80. See **Fig 2E** for quantification. IMCD3, inner medullary collecting duct; Nb, nanobody; SMO, Smoothened.
(PDF)

S4 Fig Controls for SMO/PKA-C BRET studies. Related to **Fig 3**. (**A**) Nanoluc-tagged SMO674 and SMO566 (see **Fig 2**) were subject to BRET analysis with YFP-tagged βarrestin1 (black), PKA-C (blue), or NbSmo2 (gray), as described in **Fig 3A**. (**B**) Nanoluc fusions of successive SMO CT truncations (SMO, SMO657, SMO614, SMO574, and SMO566) were utilized to determine the region of the pCT required for efficient BRET with PKA-C. Cartoon above the graph indicates the position of each CT truncation. (**C**) Saturation analysis of BRET between SMO and PKA-C. Fixed amounts of SMO BRET donor or 2 negative control BRET donors (PTCH1 or the DRD2), were cotransfected with increasing amounts of PKA-C BRET acceptor. The x-axis reflects levels of PKA-C, assessed via external excitation of YFP prior to nanoluc substrate addition (YFP_ext._) normalized to levels of SMO (nanoluc), and the y-axis reflects the BRET ratio. See “Methods” for more information. (**D**) SMO657 BRET with NbSmo2, NbSmo8, Nbβ2AR80, βarrestin1, or βarrestin2. To determine if BRET depends on SMO activity, cells were treated for 1 hour with SAG21K (1μM) or KAADcyc (1μM). (**E**) BRET using SMO657 or SMO566 as donors and Nbβ2AR80, NbSmo2, or SUFU as acceptors, performed as described in (D). **(F)** Expression levels of BRET acceptor for the experiment shown in **Fig 3B**, measured as background-subtracted YFP fluorescence (via external excitation) prior to addition of nanoluc substrate. The underlying data for this figure can be found under **S3 Data**. See **S1 Table** for statistical analysis. BRET, bioluminescence resonance energy transfer; DRD2, D2 dopamine receptor; KAADcyc, KAAD-cyclopamine; Nb, nanobody; pCT, proximal segment of the cytoplasmic tail; PKA-C, PKA catalytic subunits; PTCH1, Patched1; SMO, Smoothened; SUFU, suppressor of Fused.
(PDF)

S5 Fig Generation of cell lines containing BRET donor at endogenous *Smo* locus and studies of interactions between soluble SMO pCT and PKA-C. Related to **Fig 3**. (**A**) BRET in IMCD3 cells transiently transfected with the indicated nanoluc-tagged donors and PKA-C-YFP. (**B**) Schematic of strategy to modify endogenous *Smo* locus for BRET experiments using a HiBiT/LgBiT split nanoluc strategy combined with CRISPR/Cas9 [135,148]. In brief, the 11 amino acid HiBiT peptide was appended to the carboxyl terminus of SMO via CRISPR/Cas9; LgBiT, introduced on a plasmid via transient transfection, binds HiBiT with high affinity, thereby reconstituting a nanoluc BRET donor at the carboxyl terminus of endogenous SMO. As neither HiBiT nor LgBiT are luminescent on their own, this strategy results in selective attachment of a functional nanoluc molecule to the carboxyl terminus of endogenous SMO protein, and the CRISPR/Cas9 step is efficient because insertion of only a short sequence is required. (**C**) Left: PCR from genomic DNA of IMCD3 parental cells or the genome-edited SMO-HiBiT clone, consistent with heterozygous modification of the *Smo* locus. Molecular masses are in base pairs. Right: Expression of SMO-HiBiT protein (predicted molecular mass = 89.0 kDa), assessed using a Nano-Glo HiBiT Blotting System, vs. total protein in cell lysates assessed using Stain Free imaging. Parental IMCD3 cells serve as a negative control. Molecular masses are in kDa. (**D**) A Protein C- and nanoluc-tagged SMO pCT construct lacking the extracellular and 7TM domains (SMO555-674) was expressed in HEK293 cells, which were analyzed by immunofluorescence microscopy. A FLAG-tagged β2AR construct marks the plasma membrane. (**E**) BRET between βarrestin1 (black) or PKA-C (blue), and the Protein C- and nanoluc-tagged SMO pCT construct used in (D) (SMO555-674, see cartoon above). The same construct lacking SMO sequences (“Neg.”) serves as a negative control. The underlying data for this figure can be found under **S3 Data**. The uncropped DNA gel, western, and protein gel are included in **S8 Data**. See **S1 Table** for statistical analysis. 7TM, seven-transmembrane; BRET, bioluminescence resonance energy transfer; IMCD3, inner medullary collecting duct; pCT, proximal segment of the cytoplasmic tail; PKA-C, PKA catalytic subunits; SMO, Smoothened.
(PDF)

S6 Fig Controls for SMO/PKA-C crosslinking studies. Related to **Fig 3**. YFP quantification of (**A**) FLAG elution fractions or (**B**) input fractions from SMO/PKA-C copurification performed in the absence (black) or presence (purple) of 0.5 mM DSP crosslinker, as presented in **Fig 3E**. (**C**) Protein gels of input fractions for the experiment shown in **Fig 3E**. Top panel: anti-FLAG western blot to detect SMO (note that SMO674 and SMO566 run at distinct positions on the gel, as expected); Middle panel: total protein (detected via in-gel Stain Free imaging); Bottom panel: YFP (in-gel YFP fluorescence). The decrease in soluble protein yields in total cell lysates that occurs at high DSP concentrations may be due to crosslinker-induced protein aggregation and loss of solubility. Molecular masses are in kDa. The uncropped protein gels and western are included in **S8 Data**. DSP, dithiobis(succinimidyl propionate); PKA-C, PKA catalytic subunits; SMO, Smoothened.
(PDF)

S7 Fig Controls for PKA-R BRET experiments and PKA-C mutagenesis experiments. Related to **Fig 4**. (**A**) Saturation BRET analysis using a fixed amount of SMO657-nanoluc donor and varying amounts of YFP-tagged PKA-C or PKA-R acceptors, performed and analyzed as in **S4B Fig**. (**B**) Western blot analysis of untagged and YFP-tagged PKA-R constructs from the experimental setup in **Fig 4C**, using an anti-PKA-R antibody. (**C**) Expression levels for the YFP-tagged PKA N-tail mutants in **Fig 4D**, assessed via background-subtracted external excitation of the YFP fluorophore prior to addition of nanoluc substrate. The underlying data for this figure can be found under **S4 Data**. The uncropped western is included in **S8 Data**. See **S1 Table** for statistical analysis. BRET, bioluminescence resonance energy transfer; PKA-C, PKA catalytic subunits; PKA-R, PKA regulatory subunits; SMO, Smoothened.
(PDF)

S8 Fig Controls for assays to look at SMO activity- and GRK2/3-dependent interactions. Related to **Fig 5**. (**A**) HEK293 cells transfected with FLAG-tagged SMO674 (magenta) and YFP-tagged NbSmo2 or GFP-tagged Nbβ2AR80 (green) were treated with KAADcyc or SAG21k and imaged as described in **Fig 5B**. (**B**) Line scan analysis of images from cells expressing SMO674 (**Fig 5B**) or SMO566. Cells were treated as described in **Fig 5B**. (**C**) Quantification of colocalization between SMO and NbSmo2 or Nbβ2AR80 for the experiment in (A) (see “Methods”). (**D**) Western analysis of SMO (anti-FLAG) and PKA-C (anti-YFP) expression levels in lysates from HEK293 cells treated with the indicated drugs as described in **Fig 5D**. (**E**) Concentration–response analysis of Cmpd101 effects on SMO regulation of PKA-C in the CREB reporter assay, revealing an IC_50_ of 1.87 μM (95% confidence interval = 0.96–3.57 μM.) (**F**) CREB reporter assay performed in wild-type HEK293 cells (“Parental”) vs. cells modified via CRISPR/Cas9 to lack endogenous GRK2, 3, 5, and 6 (“ΔGRK” cells, see “Methods”). Cells were transfected with PKA-C, either alone (black) or with SMO674 (white), GRK2 (mustard), or both (lavender). The underlying data for this figure can be found under **S5 Data**. The uncropped westerns are included in **S8 Data**. See **S1 Table** for statistical analysis. Cmpd101, Compound 101; CREB, cyclic AMP response element binding protein; GRK, GPCR kinase; KAADcyc, KAAD-cyclopamine; Nb, nanobody; PKA-C, PKA catalytic subunits; SMO, Smoothened.
(PDF)

S9 Fig Full sequence alignments and quantification info for MS. Related to **Fig 6**. (**A**) Full alignment of SMO from **Fig 6B**, noting conservation of GRK2/3 among vertebrates, insects and basal metazoans. Interestingly, some GRK2/3 phosphorylation sites in mouse SMO are charged (D or E) residues in dSmo (i.e., S560), and vice versa (i.e., D601), consistent with the importance of negative charges at those positions. dSmo phosphorylation sites are from [80], in which sites verified as GRK-dependent are indicated in dark green, while sites that might be GRK-dependent but were not covered during their MS are indicated in light green. (**B**) Quantification of individual SMO and/or GRK2/3 activity-dependent phosphorylation sites and phosphorylation clusters from the targeted MS analysis in **Fig 6B and 6C**. Note that the S560 graph in this figure is identical to the cluster “a” graph in **Fig 6C** because cluster “a” comprises only a single phosphorylated residue. Unambiguous phosphorylation site localization on peptides with multiple candidate phosphorylation sites in close proximity can be challenging by MS [149] (see “Methods”). For these reasons, our quantification, and subsequent mutational analysis, focuses on clusters of residues rather than individual sites. In each graph, “/” means “or,” indicating that at least one of the sites listed here is phosphorylated, while “_” indicates that both of the sites are phosphorylated. *n* = 4 biological replicates per condition. The underlying data for this figure can be found under **S6 Data**. GRK, GPCR kinase; MS, mass spectrometry; SMO, Smoothened.
(PDF)

S10 Fig Controls for SMO sequence coverage in MS experiments. Related to **Fig 6**. Snake plot of the SMO674 construct used in this study. Residues covered by the global MS measurements are colored aqua, while residues that were not detected by MS are colored white. TM helices are numbered in navy. Regions potentially accessible to intracellular kinases (ICLs 1, 2, and 3 and the pCT domain) are labeled. Color coding of phosphorylation sites is identical to **Figs 7** and **S9**. Residue numbering is with respect to untagged, full-length wild-type SMO. Signal sequence and N-terminal tags are omitted for clarity. ICL, intracellular loop; MS, mass spectrometry; pCT, proximal segment of the cytoplasmic tail; SMO, Smoothened.
(PDF)

S11 Fig Ciliary localization studies. Related to **Fig 7**. Smo^−/−^ fibroblasts expressing tdTomato-tagged (**A**) SMO-Nb fusions, or (**B**) the SMO Ala mutant were visualized via microscopy, following staining with antibodies to detect cilia (Arl13b), indicated in green. SMO is indicated in magenta. Representative images are shown, and the intensities of the ciliary SMO signals for wild-type SMO vs. the Ala mutant were quantified in (**C**) as described in “Methods” (*n* = 30 cilia per condition). DAPI counterstain (blue) marks the nucleus. Scale bar = 2.5 μm. The underlying data for this figure can be found under **S7 Data**. Nb, nanobody; SMO, Smoothened.
(PDF)

S1 Table Tests of statistical significance. Related to Figs 1–7 and S1–S11.
(PDF)

S2 Table Initial identification and quantification of SMO phosphorylation sites via untargeted mass spectrometry. Related to Figs 6 and S9. MS, mass spectrometry; SMO, Smoothened.
(PDF)

S1 Data Data underlying Figs 1B and 1D and S1C and S1E–S1G. (XLSX)

S2 Data Data underlying Fig 2C and 2E. (XLSX)

S3 Data Data underlying Figs 3A–3D and S4A–S4F and S5A and S5E. (XLSX)

S4 Data Data underlying Figs 4B–4F and S7A and S7C. (XLSX)

S5 Data Data underlying Figs 5A and 5C–5E and S8C, S8E and S8F. (XLSX)

S6 Data Data underlying Figs 6C and 6D and S9B. (XLSX)

S7 Data Data underlying Figs 7A, 7B, 7D and 7F and S11C. (XLSX)

S8 Data Raw blots and gels underlying Figs 1C, 3C and 3E, 6A and 7C and S1D, S5C, S6C, S7B and S8D. (PDF)

## Abbreviations

7TM: seven-transmembrane
AC: adenylyl cyclase
AKAP: A-kinase anchoring protein
BRET: bioluminescence resonance energy transfer
cAMP: cyclic AMP
Cmpd101: Compound 101
Cos2: Costal2
CRE: cyclic AMP response element
CREB: cyclic AMP response element binding protein
CT: cytoplasmic tail
dCT: distal segment of the cytoplasmic tail
DDA: data dependent analysis
DRD2: D2 dopamine receptor
DSP: dithiobis(succinimidyl propionate)
EVC2: Ellis van Creveld protein 2
FSEC: fluorescence detection size exclusion chromatography
GLI: glioma-associated
GPCR: G protein–coupled receptor
GRK: GPCR kinase
HBSS: Hanks’ balanced saline solution
HCD: higher energy collisional dissociation
Hh: Hedgehog
hpf: hours postfertilization
ICL3: third intracellular loop
IMCD3: inner medullary collecting duct
IRES: internal ribosome entry site
KAADcyc: KAAD-cyclopamine
M2AchR: M2 acetylcholine receptor
MS: mass spectrometry
nanoluc: nano-luciferase
Nb: nanobody
N-tail: N-terminal tail
pCT: proximal segment of the cytoplasmic tail
PKA: protein kinase A
PKA-C: PKA catalytic subunits
PKA-R: PKA regulatory subunits
PLB: passive lysis buffer
ppm: parts per million
PRM: parallel reaction monitoring
PTCH1: Patched1
RLU: relative luciferase unit
RLU: relative luciferase unit
ShhN: N-terminal signaling domain of Sonic hedgehog
SUFU: suppressor of Fused
TFA: trifluoroacetic acid
TM5-6: transmembrane helices 5 and 6
