# Supplementary figures and images for: Smoothened transduces Hedgehog signals via activity-dependent sequestration of PKA catalytic subunits
Source: PLoS Biol. 2021 Apr 22;19(4):e3001191. doi: 10.1371/journal.pbio.3001191 (PMC8096101; doi:10.1371/journal.pbio.3001191)

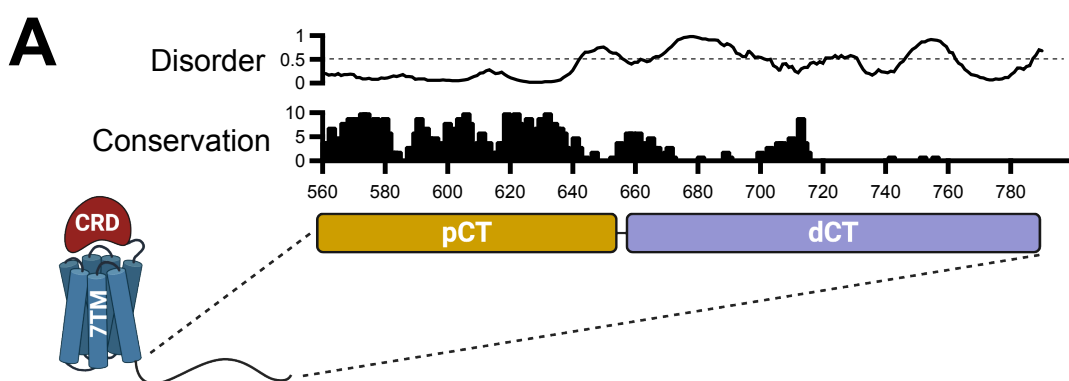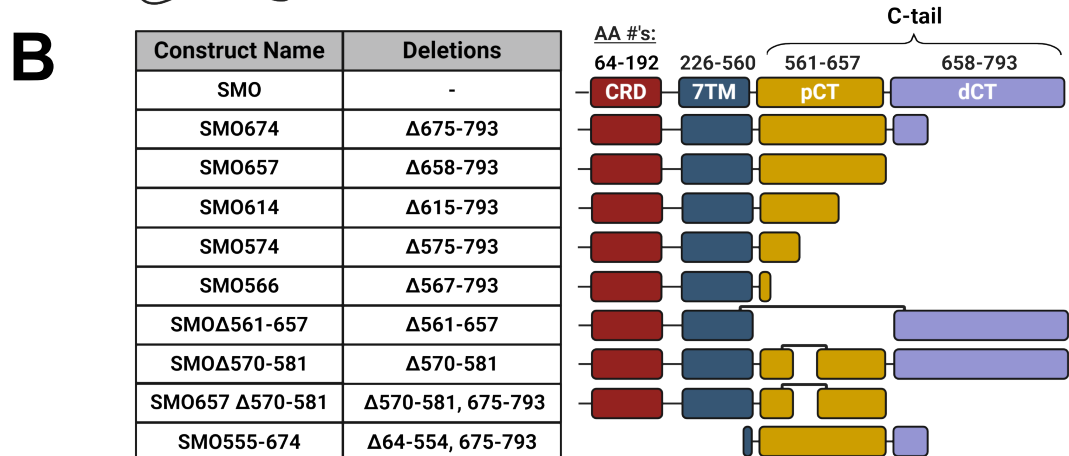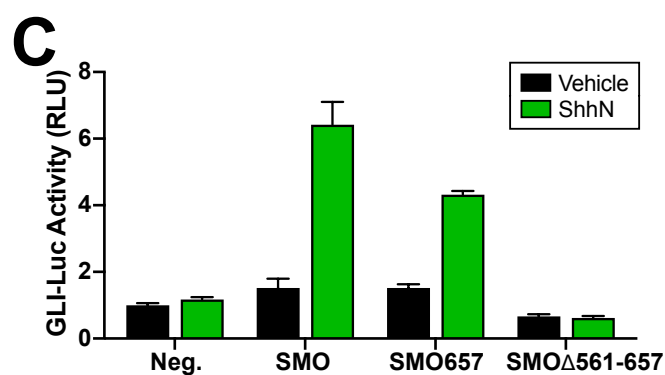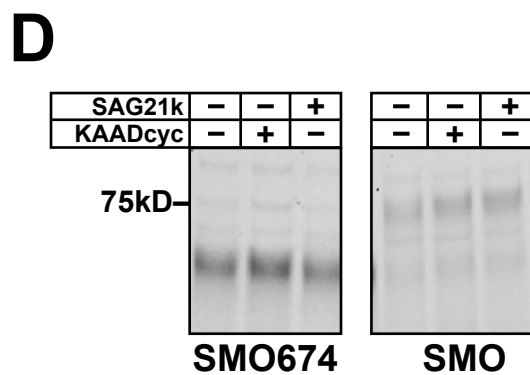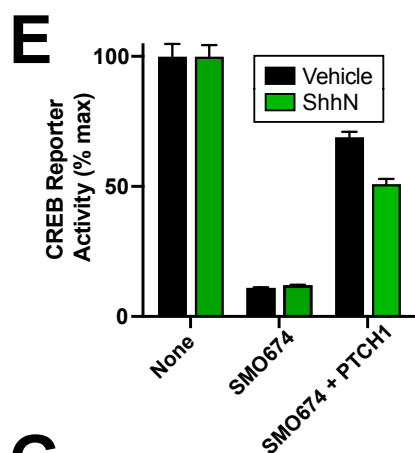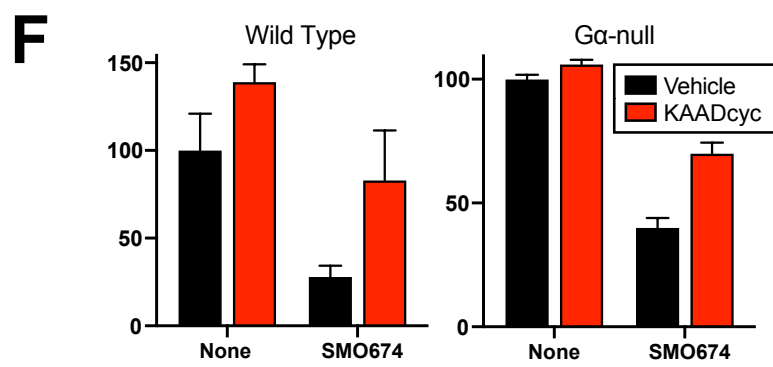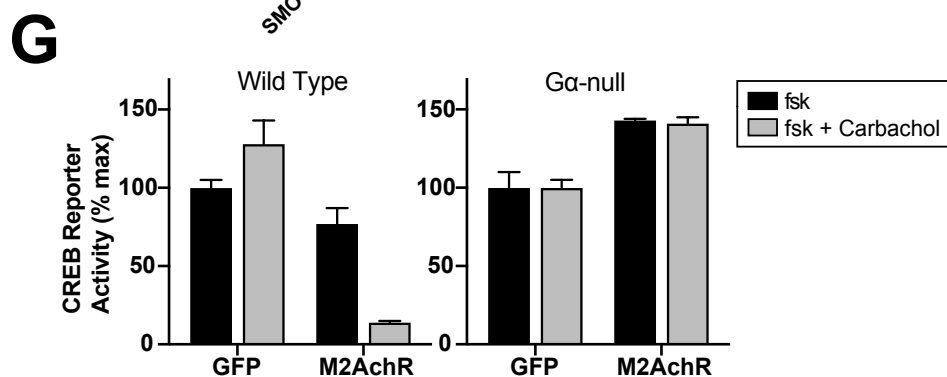

Supplement: S1 Fig — Related to Fig 1. (A) Jalview conservation score (0–10) and DISOPRED disorder score (0–1, with values >0.5 indicative of disorder) are plotted on the y-axes, while SMO amino acid numbering is plotted on the x-axis. Locations of pCT (mustard) and dCT (lavender) are indicated below the graphs. (B) Table of SMO constructs used in this study. Note that CRD (red) and 7TM (blue) domains are not drawn to scale. We used SMO657, truncated immediately after the pCT, for many of our cell-based experiments, based on its ability to recapitulate >70% of the activity of wild-type SMO in GLI reporter assays (see (C)). However, secondary structure predictions revealed a conserved region between amino acids 657–674, predicted to be ordered. Because these parameters might help to increase the biochemical stability of SMO, we extended the construct boundary (SMO674) in any experiments requiring purification of SMO protein. (C) Smo−/− MEFs were transfected with GLI-luciferase reporter plasmid, along with a GFP negative control (“Neg.”), full-length SMO, or truncation mutants lacking the dCT (SMO657) or pCT (SMOΔ561–657). Following transfection, cells were stimulated with conditioned medium containing the N-terminal signaling domain of Sonic hedgehog (ShhN, green) or control, non-ShhN-containing conditioned medium (Vehicle, black). (D) FLAG-tagged full-length SMO or SMO674 were expressed in HEK293S GnTI- cells, stimulated with the SMO agonist SAG21k or the SMO inverse agonist KAADcyc for 4 hours (to assess possible effects of these compounds on SMO expression levels), then solubilized in detergent and purified over M1 FLAG resin. Elution was analyzed by SDS-PAGE and total protein staining (StainFree imaging). Note that SMO tends to migrate slightly faster than expected in these experiments, likely due to a reduction in glycosylation in GnTI- cells compared to their wild-type counterparts; (E) HEK293 cells were transfected with the indicated plasmids, treated with vehicle or ShhN for 4 ho [file pbio.3001191.s001.pdf]

**A**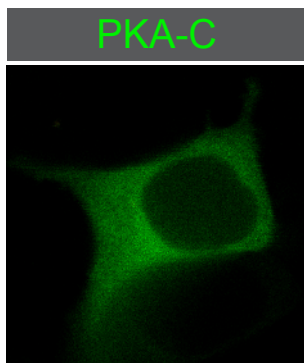**B**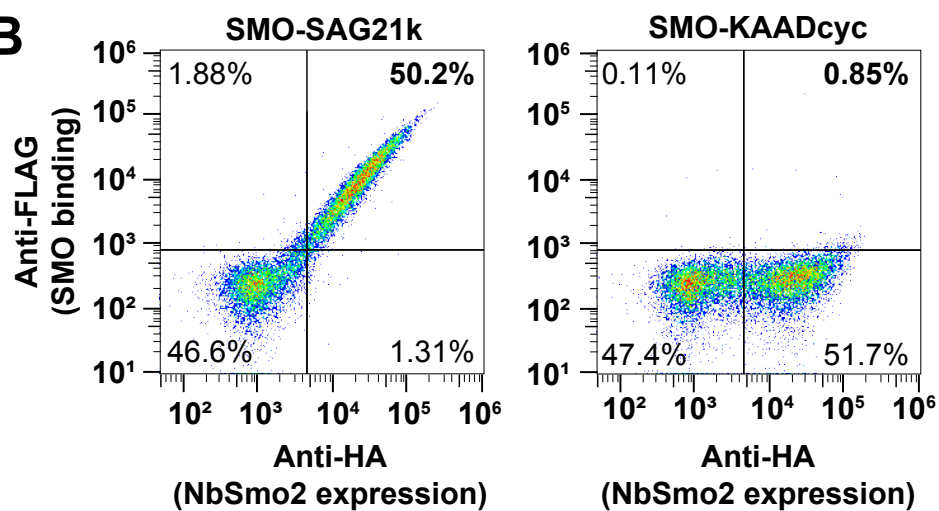**C**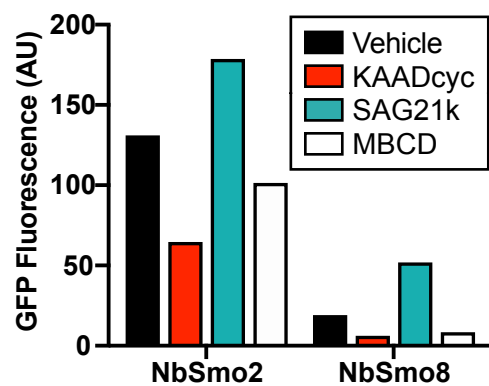**D**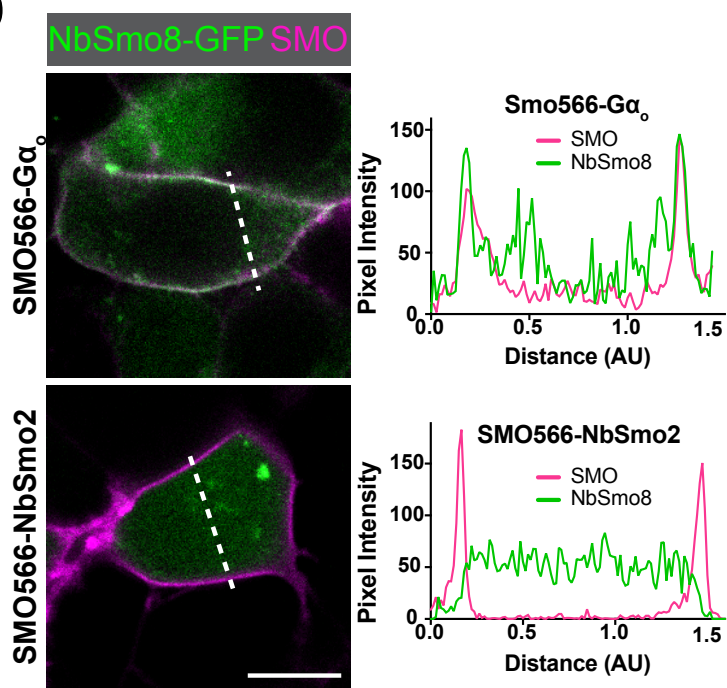**E**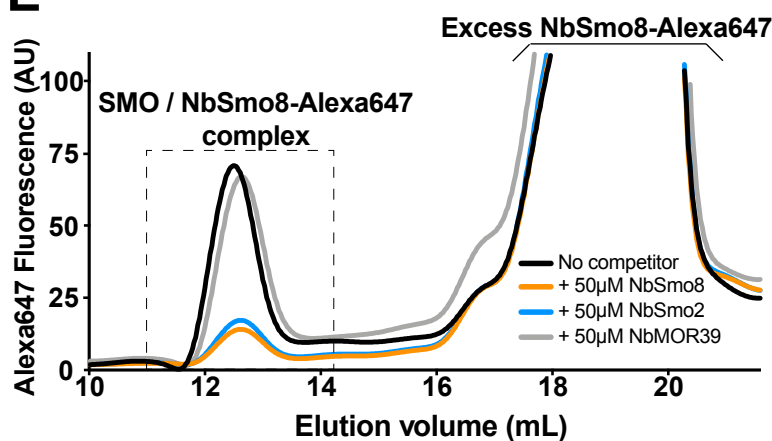

Supplement: S2 Fig — Related to Fig 2. (A) Representative image of PKA-C localization in HEK293 cells not expressing SMO. (B) Binding of NbSmo2, displayed on the surface of yeast [90], to purified, detergent-solubilized SMO-agonist (SAG21k) complexes or SMO-inverse agonist (KAADcyc) complexes in solution, was assessed by flow cytometry. Note that this experiment used SMO566, which lacks the entire cytoplasmic tail. (C) FLAG-tagged SMO566-Gαo was expressed in HEK293 cells alone or with GFP-tagged NbSmo2, NbSmo8, or Nbβ2AR80. Following treatment with SMO agonist (SAG21k, 1 μM), inverse agonist (KAADcyc, 1 μM), or MBCD (8 mM, which extracts SMO sterol agonists from membranes [42]), SMO-Nb complexes were isolated from detergent-solubilized cells via FLAG affinity chromatography and Nb levels measured via GFP fluorescence quantification. Ratios of GFP fluorescence in FLAG eluates, normalized to GFP fluorescence in each lysate before affinity chromatography, are reported. (D) NbSmo8-GFP colocalization with SMO566-NbSmo2 fusion at the cell membrane. The presence of NbSmo2 is predicted to prevent binding of NbSmo8 to SMO if the Nbs bind to overlapping epitopes. SMO566-Gαo serves as a positive control. Line scan analysis is shown to the right of each merged image, with a dotted line indicating the location of the scan. (E) In vitro binding of Alexa647-labeled NbSmo8 to SMO566 in the presence of non-fluorescent NbSmo2 competitor, as assessed by FSEC. Non-fluorescent NbSmo8 or NbMOR39 (which binds a non-SMO GPCR [147]) serve as positive and negative controls for NbSmo8 competition binding, respectively. FSEC, fluorescence detection size exclusion chromatography; GPCR, G protein–coupled receptor; KAADcyc, KAAD-cyclopamine; MBCD, methyl-β-cyclodextrin; Nb, nanobody; PKA-C, PKA catalytic subunits; SMO, Smoothened. (PDF) [file pbio.3001191.s002.pdf]

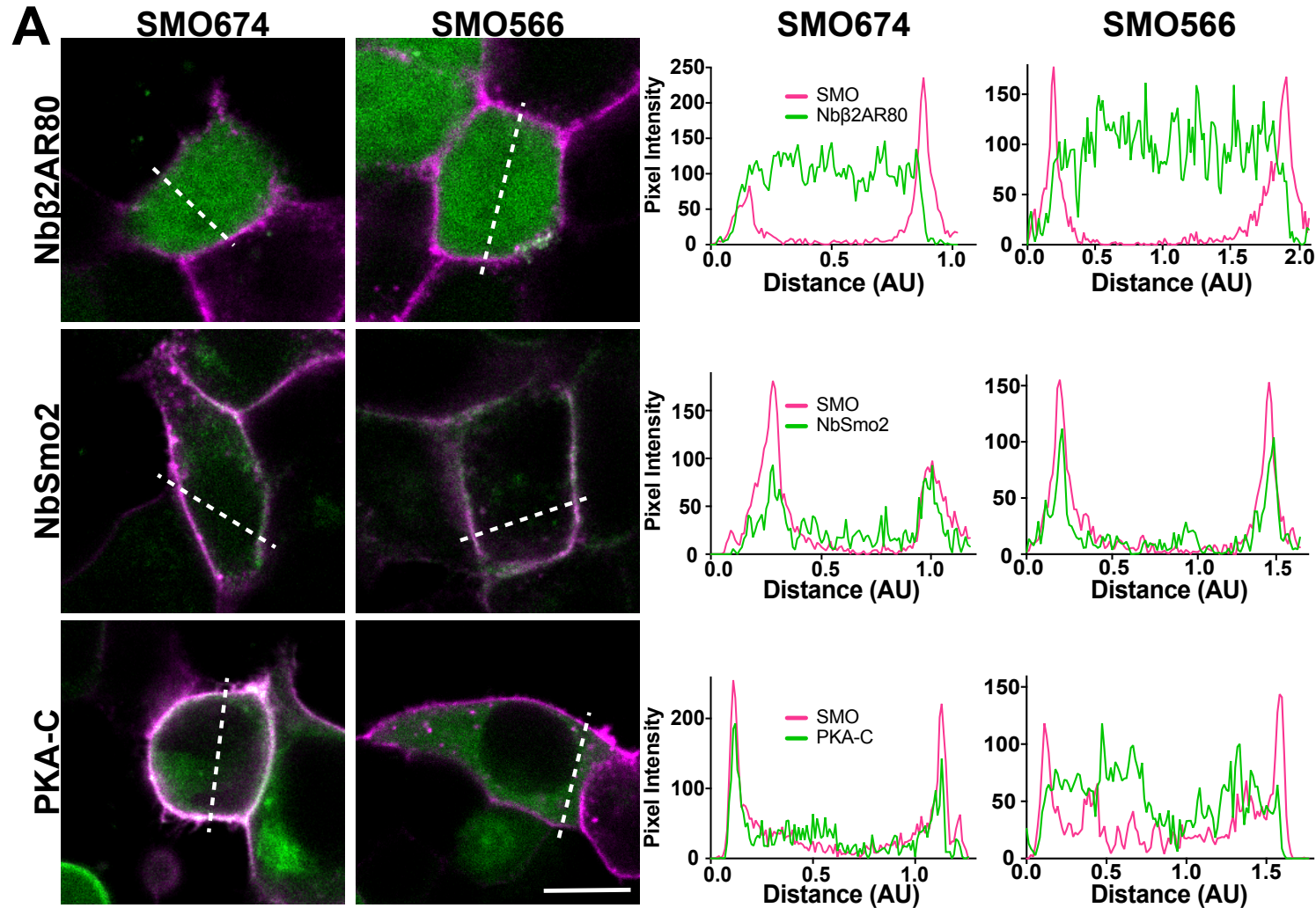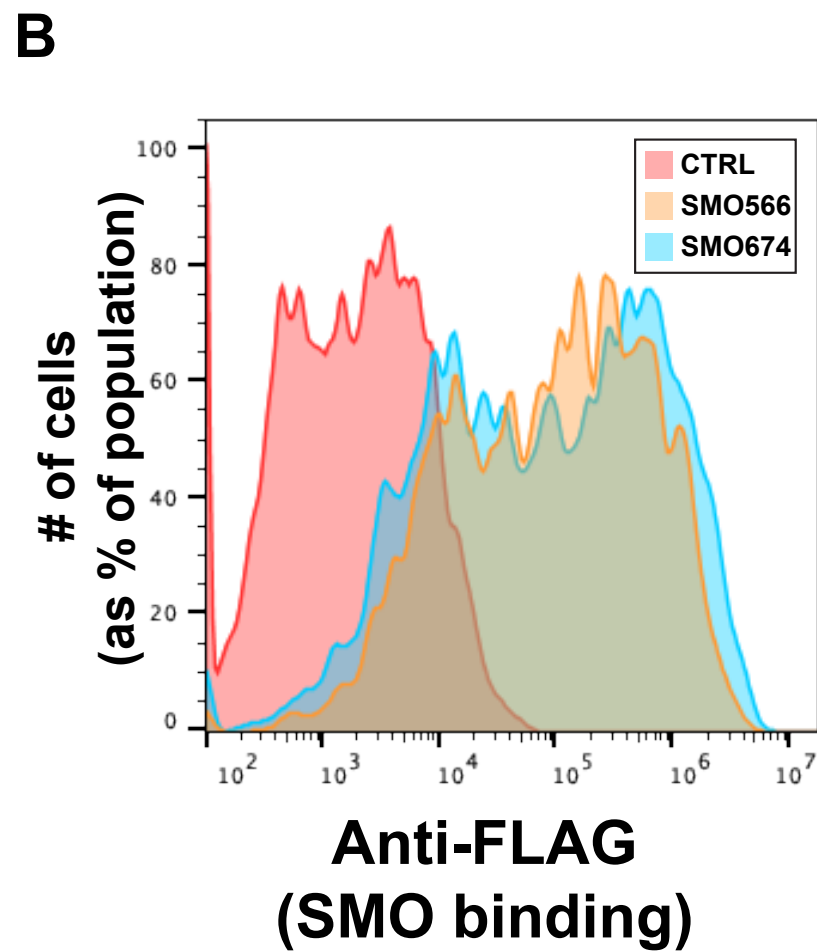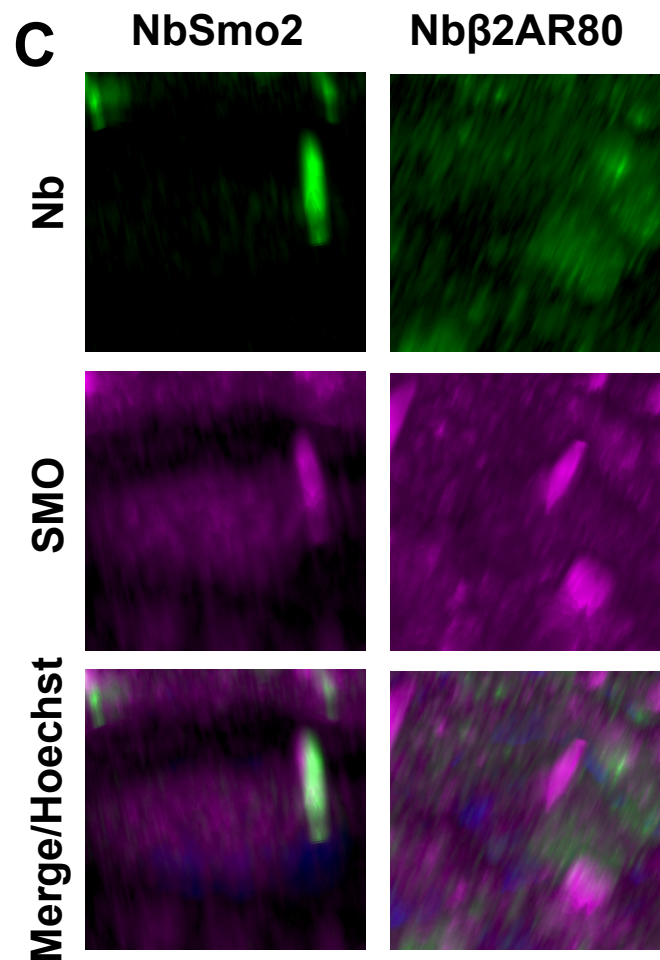

Supplement: S3 Fig — Related to Fig 2. (A) Line scans for colocalization images in Fig 2A and 2B. Colors are the same as described in the main figure panels. Dotted line indicates location of the scan. (B) Surface expression of SMO674 and SMO566 in HEK293 cells was assessed via FACS staining of nonpermeabilized cells with an FLAG-Alexa 647 conjugate. HEK293 cells not expressing SMO serve as a negative control (“CTRL”). (C) Raw data (3D reconstruction) of stable IMCD3 cells coexpressing FLAG-tagged SMO and mNeonGreen-tagged NbSmo2 or Nbβ2AR80. See Fig 2E for quantification. IMCD3, inner medullary collecting duct; Nb, nanobody; SMO, Smoothened. (PDF) [file pbio.3001191.s003.pdf]

**A**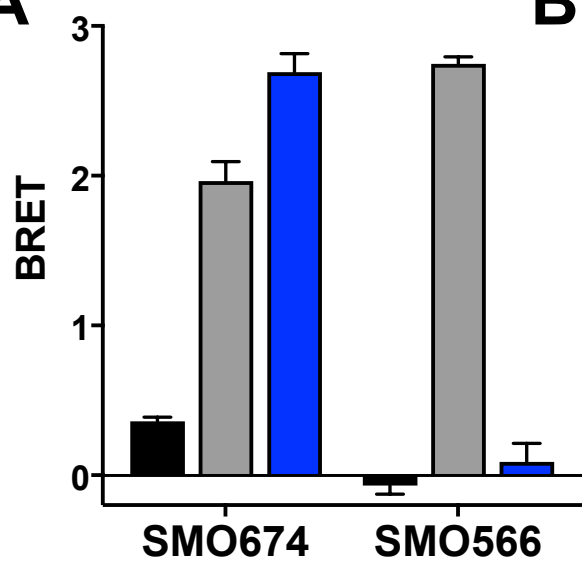**B**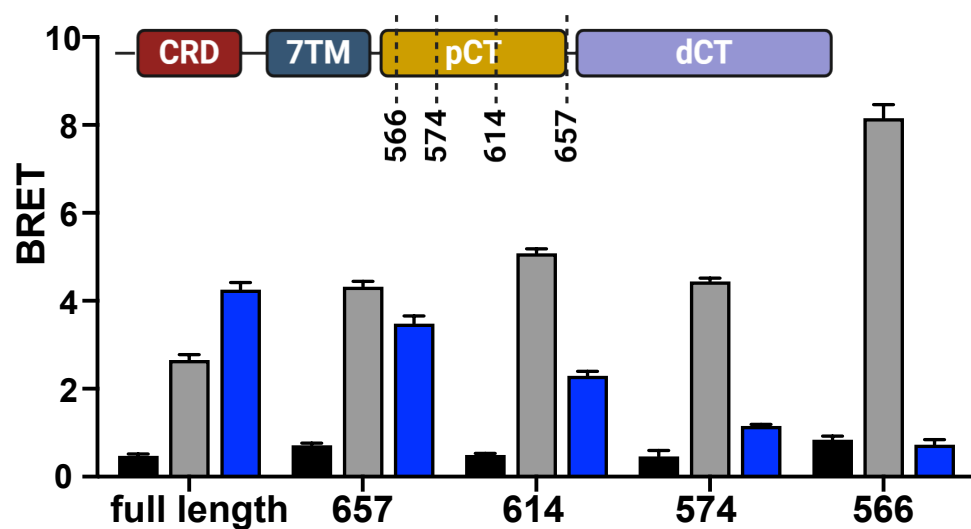**C**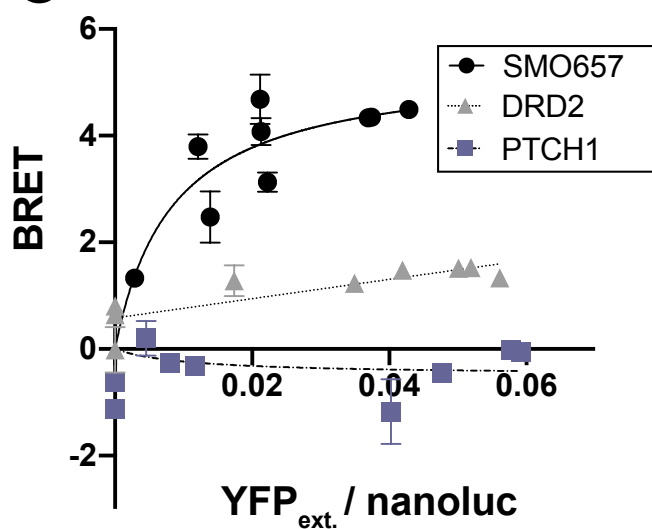**D**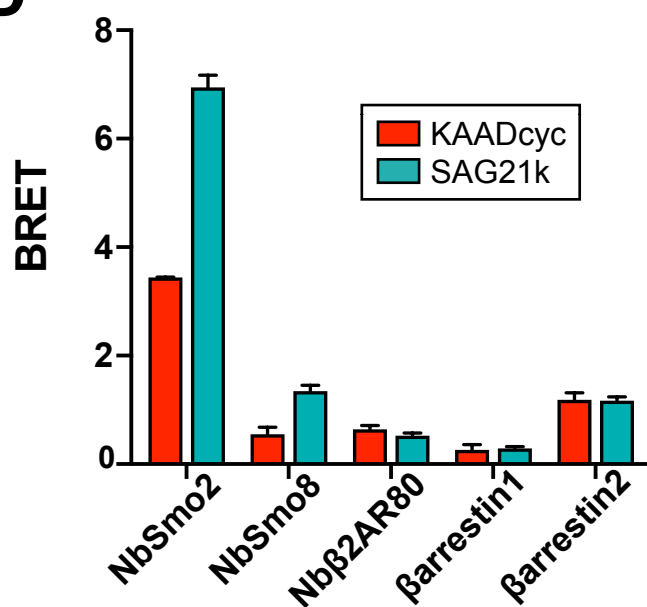**E**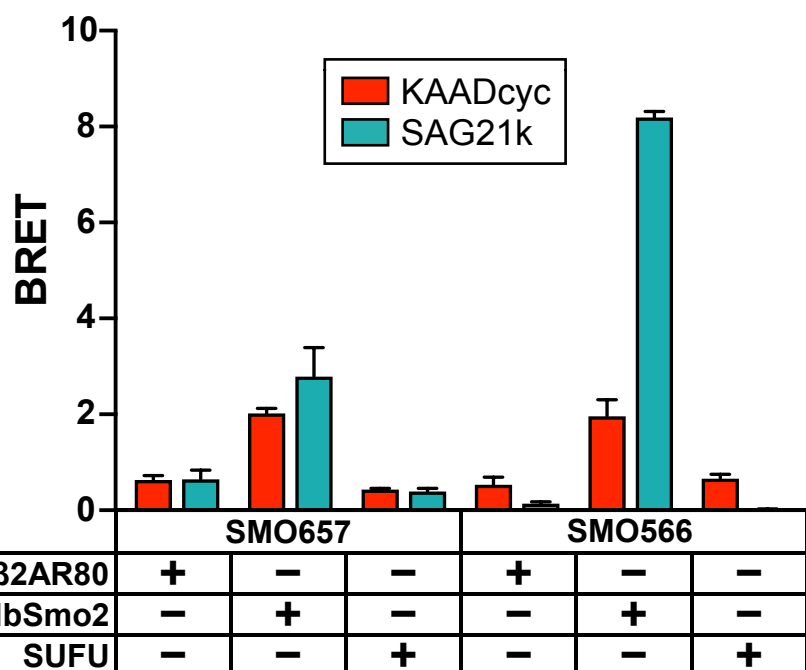**F**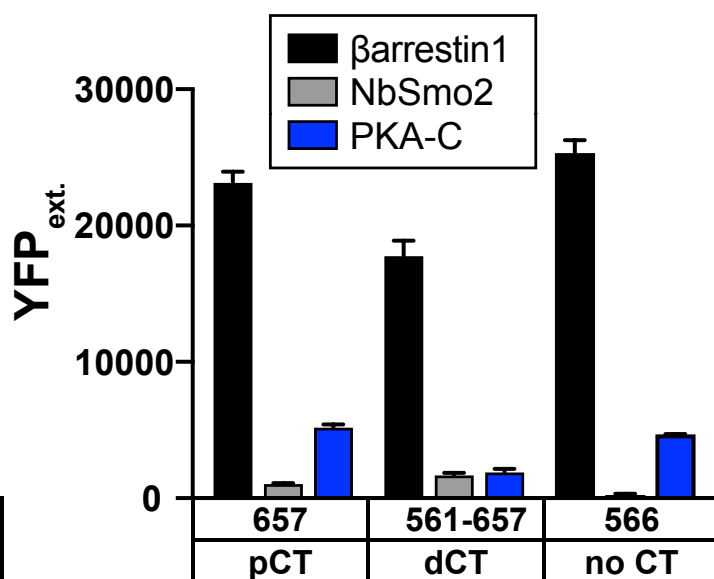

Supplement: S4 Fig — Related to Fig 3. (A) Nanoluc-tagged SMO674 and SMO566 (see Fig 2) were subject to BRET analysis with YFP-tagged βarrestin1 (black), PKA-C (blue), or NbSmo2 (gray), as described in Fig 3A. (B) Nanoluc fusions of successive SMO CT truncations (SMO, SMO657, SMO614, SMO574, and SMO566) were utilized to determine the region of the pCT required for efficient BRET with PKA-C. Cartoon above the graph indicates the position of each CT truncation. (C) Saturation analysis of BRET between SMO and PKA-C. Fixed amounts of SMO BRET donor or 2 negative control BRET donors (PTCH1 or the DRD2), were cotransfected with increasing amounts of PKA-C BRET acceptor. The x-axis reflects levels of PKA-C, assessed via external excitation of YFP prior to nanoluc substrate addition (YFPext.) normalized to levels of SMO (nanoluc), and the y-axis reflects the BRET ratio. See “Methods” for more information. (D) SMO657 BRET with NbSmo2, NbSmo8, Nbβ2AR80, βarrestin1, or βarrestin2. To determine if BRET depends on SMO activity, cells were treated for 1 hour with SAG21K (1μM) or KAADcyc (1μM). (E) BRET using SMO657 or SMO566 as donors and Nbβ2AR80, NbSmo2, or SUFU as acceptors, performed as described in (D). (F) Expression levels of BRET acceptor for the experiment shown in Fig 3B, measured as background-subtracted YFP fluorescence (via external excitation) prior to addition of nanoluc substrate. The underlying data for this figure can be found under S3 Data. See S1 Table for statistical analysis. BRET, bioluminescence resonance energy transfer; DRD2, D2 dopamine receptor; KAADcyc, KAAD-cyclopamine; Nb, nanobody; pCT, proximal segment of the cytoplasmic tail; PKA-C, PKA catalytic subunits; PTCH1, Patched1; SMO, Smoothened; SUFU, suppressor of Fused. (PDF) [file pbio.3001191.s004.pdf]

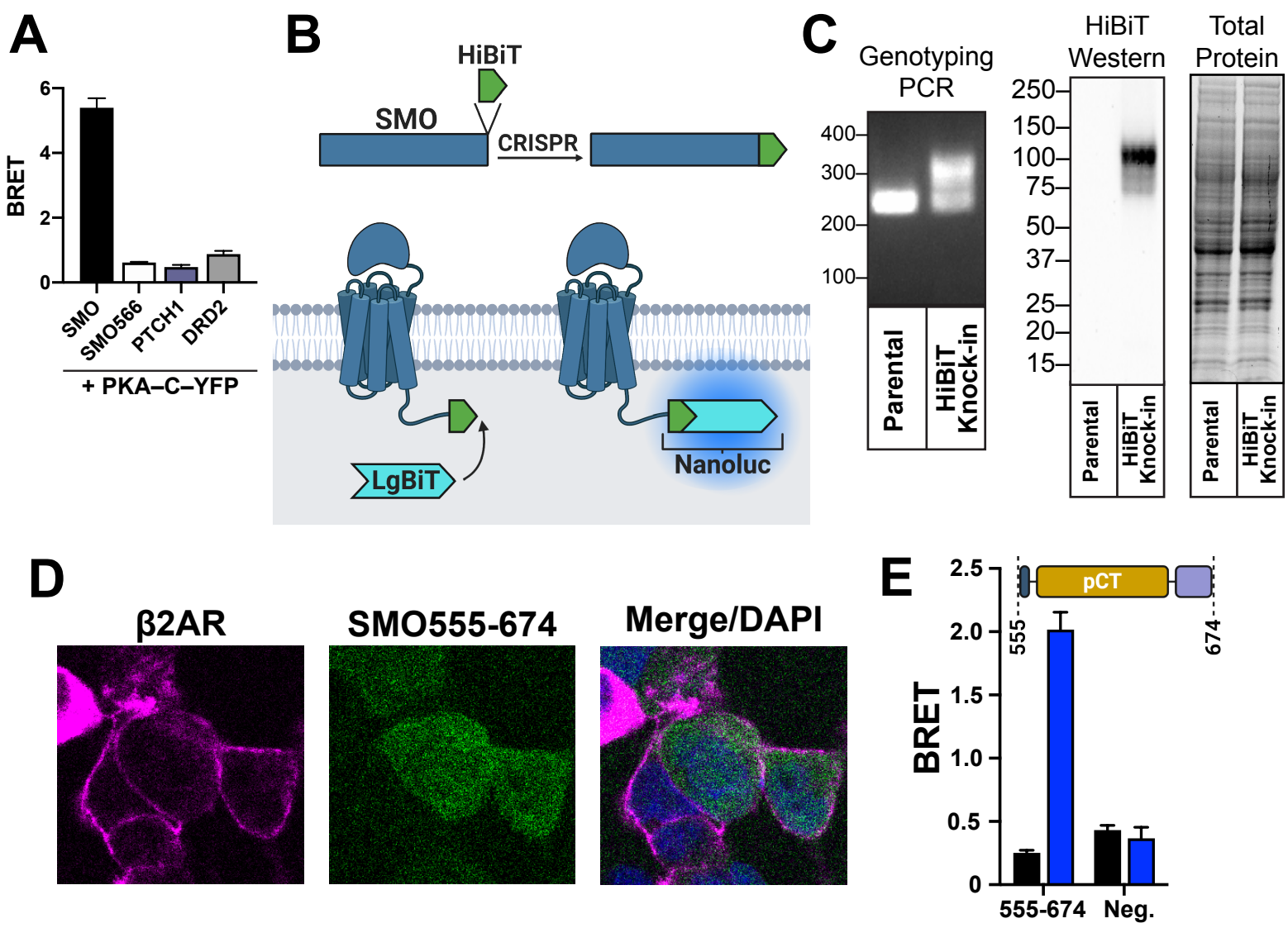

Supplement: S5 Fig — Related to Fig 3. (A) BRET in IMCD3 cells transiently transfected with the indicated nanoluc-tagged donors and PKA-C-YFP. (B) Schematic of strategy to modify endogenous Smo locus for BRET experiments using a HiBiT/LgBiT split nanoluc strategy combined with CRISPR/Cas9 [135,148]. In brief, the 11 amino acid HiBiT peptide was appended to the carboxyl terminus of SMO via CRISPR/Cas9; LgBiT, introduced on a plasmid via transient transfection, binds HiBiT with high affinity, thereby reconstituting a nanoluc BRET donor at the carboxyl terminus of endogenous SMO. As neither HiBiT nor LgBiT are luminescent on their own, this strategy results in selective attachment of a functional nanoluc molecule to the carboxyl terminus of endogenous SMO protein, and the CRISPR/Cas9 step is efficient because insertion of only a short sequence is required. (C) Left: PCR from genomic DNA of IMCD3 parental cells or the genome-edited SMO-HiBiT clone, consistent with heterozygous modification of the Smo locus. Molecular masses are in base pairs. Right: Expression of SMO-HiBiT protein (predicted molecular mass = 89.0 kDa), assessed using a Nano-Glo HiBiT Blotting System, vs. total protein in cell lysates assessed using Stain Free imaging. Parental IMCD3 cells serve as a negative control. Molecular masses are in kDa. (D) A Protein C- and nanoluc-tagged SMO pCT construct lacking the extracellular and 7TM domains (SMO555-674) was expressed in HEK293 cells, which were analyzed by immunofluorescence microscopy. A FLAG-tagged β2AR construct marks the plasma membrane. (E) BRET between βarrestin1 (black) or PKA-C (blue), and the Protein C- and nanoluc-tagged SMO pCT construct used in (D) (SMO555-674, see cartoon above). The same construct lacking SMO sequences (“Neg.”) serves as a negative control. The underlying data for this figure can be found under S3 Data. The uncropped DNA gel, western, and protein gel are included in S8 Data. See S1 Table for statistical analysis. 7TM, seven-transmembrane; BRET, [file pbio.3001191.s005.pdf]

**A**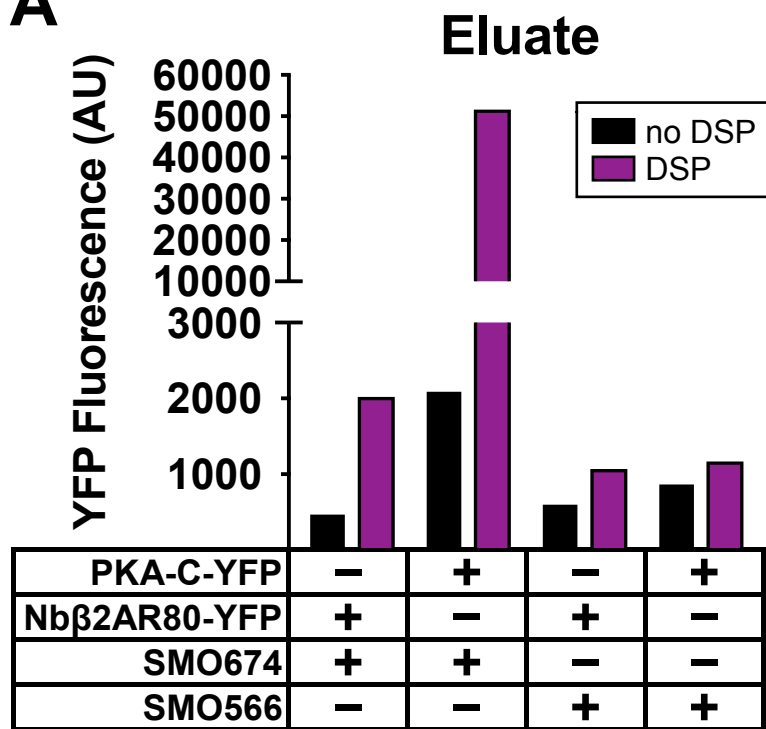**B**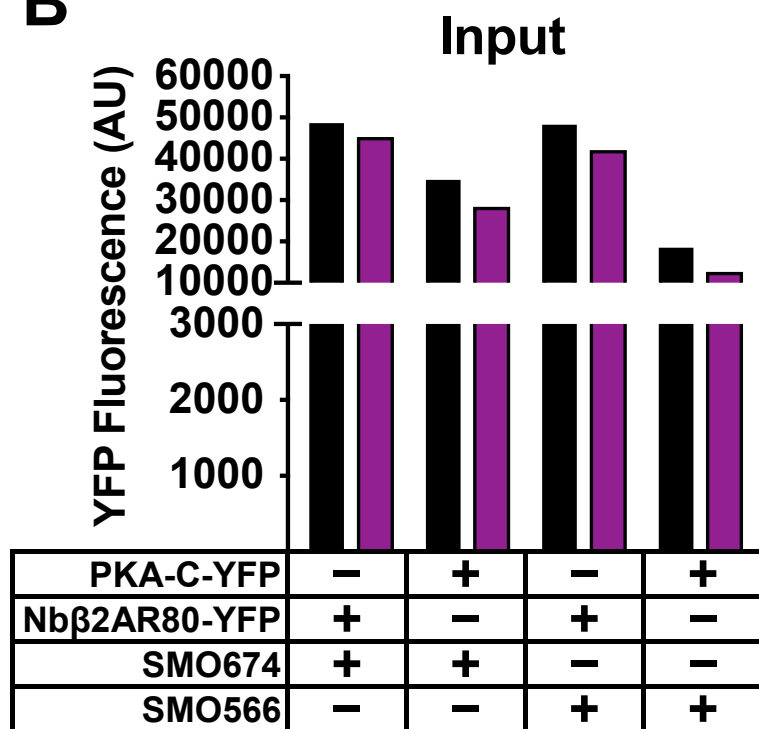**C****Input**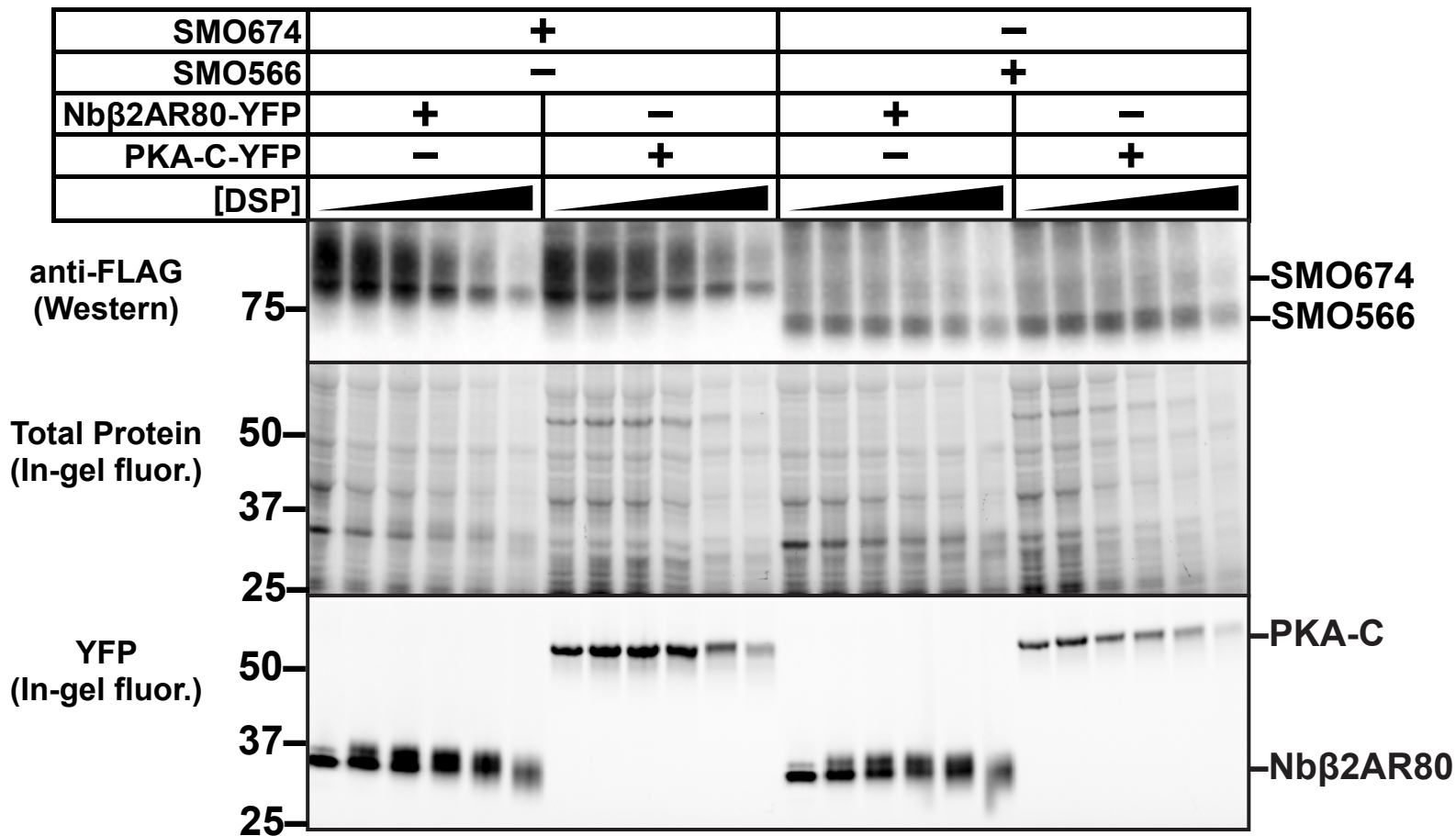

Supplement: S6 Fig — Related to Fig 3. YFP quantification of (A) FLAG elution fractions or (B) input fractions from SMO/PKA-C copurification performed in the absence (black) or presence (purple) of 0.5 mM DSP crosslinker, as presented in Fig 3E. (C) Protein gels of input fractions for the experiment shown in Fig 3E. Top panel: anti-FLAG western blot to detect SMO (note that SMO674 and SMO566 run at distinct positions on the gel, as expected); Middle panel: total protein (detected via in-gel Stain Free imaging); Bottom panel: YFP (in-gel YFP fluorescence). The decrease in soluble protein yields in total cell lysates that occurs at high DSP concentrations may be due to crosslinker-induced protein aggregation and loss of solubility. Molecular masses are in kDa. The uncropped protein gels and western are included in S8 Data. DSP, dithiobis(succinimidyl propionate); PKA-C, PKA catalytic subunits; SMO, Smoothened. (PDF) [file pbio.3001191.s006.pdf]

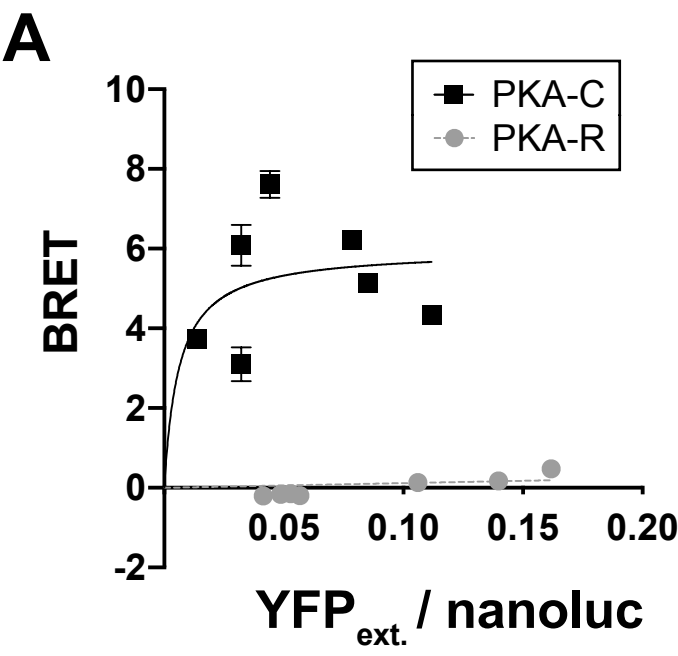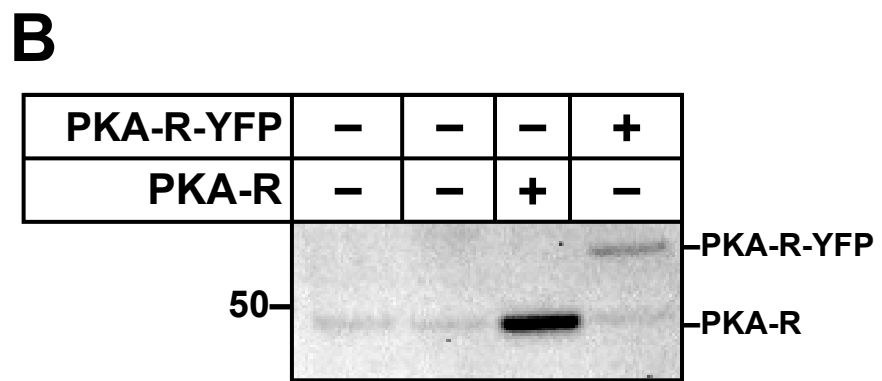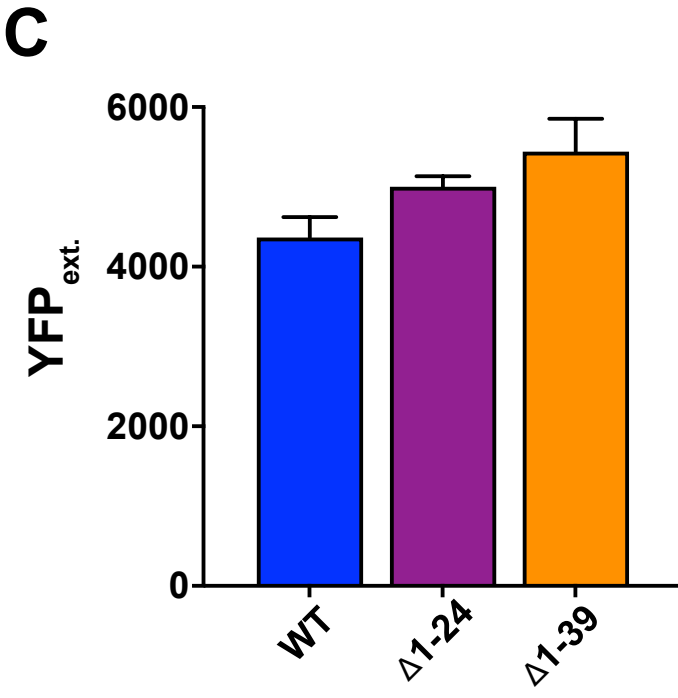

Supplement: S7 Fig — Related to Fig 4. (A) Saturation BRET analysis using a fixed amount of SMO657-nanoluc donor and varying amounts of YFP-tagged PKA-C or PKA-R acceptors, performed and analyzed as in S4B Fig. (B) Western blot analysis of untagged and YFP-tagged PKA-R constructs from the experimental setup in Fig 4C, using an anti-PKA-R antibody. (C) Expression levels for the YFP-tagged PKA N-tail mutants in Fig 4D, assessed via background-subtracted external excitation of the YFP fluorophore prior to addition of nanoluc substrate. The underlying data for this figure can be found under S4 Data. The uncropped western is included in S8 Data. See S1 Table for statistical analysis. BRET, bioluminescence resonance energy transfer; PKA-C, PKA catalytic subunits; PKA-R, PKA regulatory subunits; SMO, Smoothened. (PDF) [file pbio.3001191.s007.pdf]

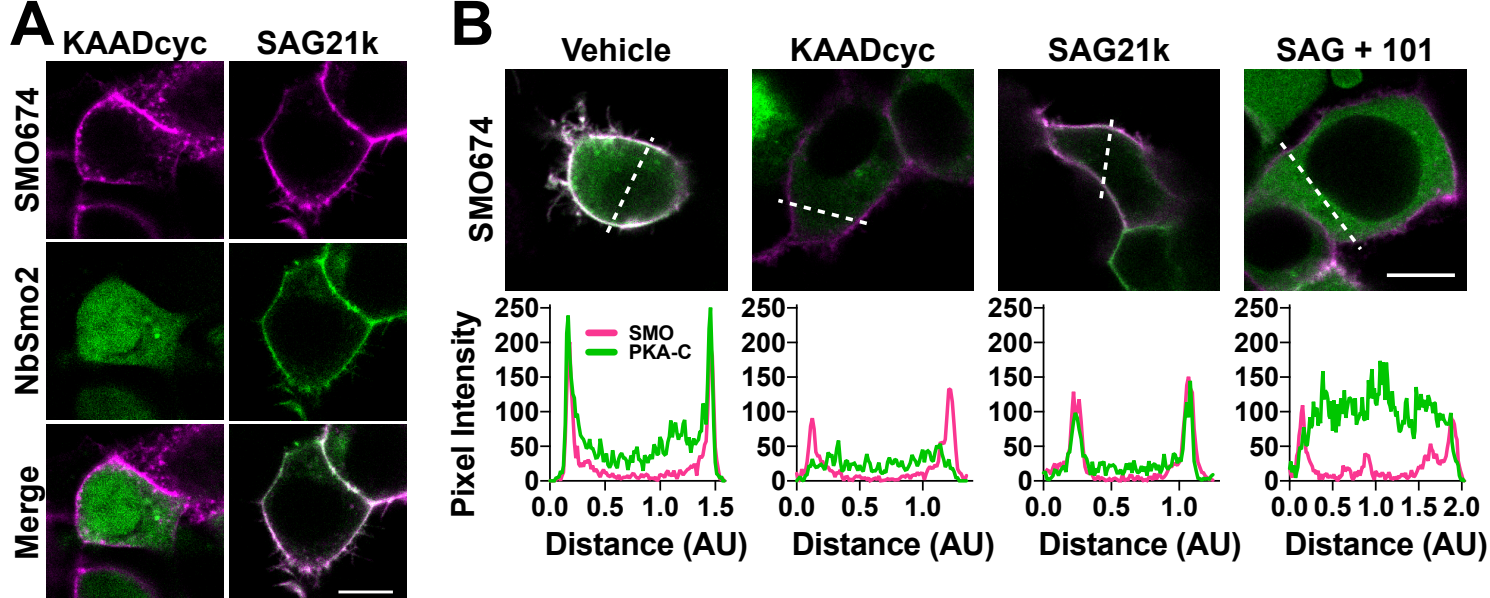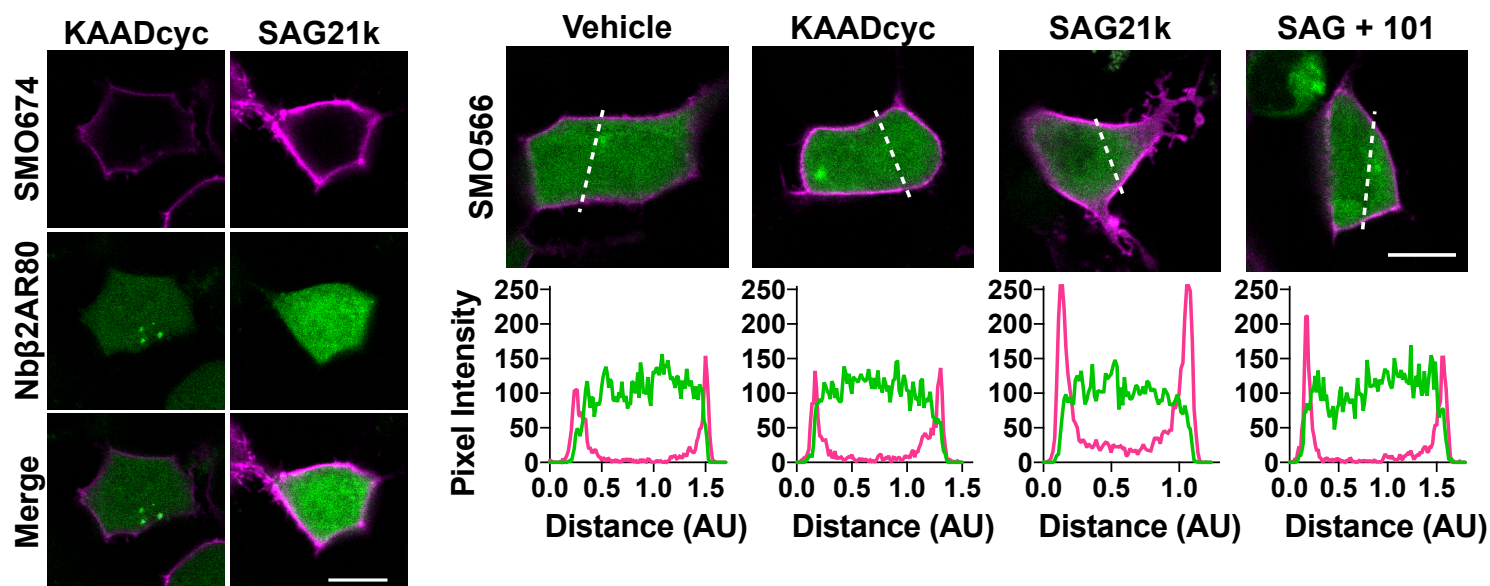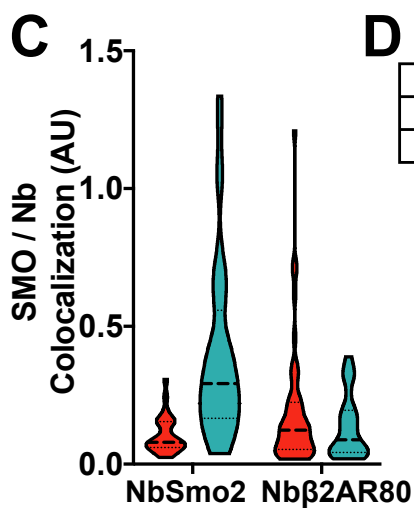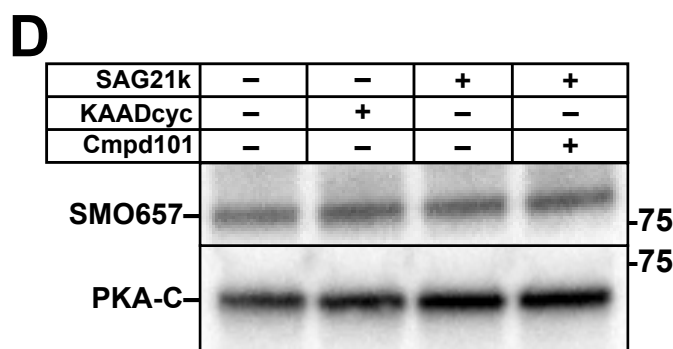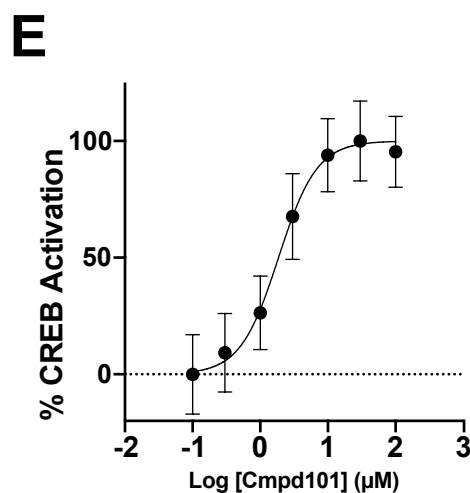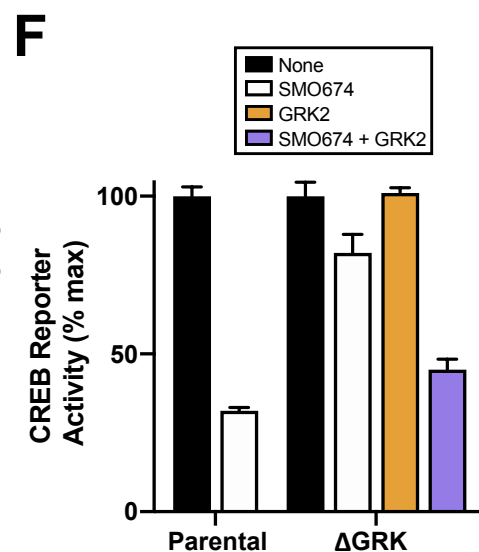

Supplement: S8 Fig — Related to Fig 5. (A) HEK293 cells transfected with FLAG-tagged SMO674 (magenta) and YFP-tagged NbSmo2 or GFP-tagged Nbβ2AR80 (green) were treated with KAADcyc or SAG21k and imaged as described in Fig 5B. (B) Line scan analysis of images from cells expressing SMO674 (Fig 5B) or SMO566. Cells were treated as described in Fig 5B. (C) Quantification of colocalization between SMO and NbSmo2 or Nbβ2AR80 for the experiment in (A) (see “Methods”). (D) Western analysis of SMO (anti-FLAG) and PKA-C (anti-YFP) expression levels in lysates from HEK293 cells treated with the indicated drugs as described in Fig 5D. (E) Concentration–response analysis of Cmpd101 effects on SMO regulation of PKA-C in the CREB reporter assay, revealing an IC50 of 1.87 μM (95% confidence interval = 0.96–3.57 μM.) (F) CREB reporter assay performed in wild-type HEK293 cells (“Parental”) vs. cells modified via CRISPR/Cas9 to lack endogenous GRK2, 3, 5, and 6 (“ΔGRK” cells, see “Methods”). Cells were transfected with PKA-C, either alone (black) or with SMO674 (white), GRK2 (mustard), or both (lavender). The underlying data for this figure can be found under S5 Data. The uncropped westerns are included in S8 Data. See S1 Table for statistical analysis. Cmpd101, Compound 101; CREB, cyclic AMP response element binding protein; GRK, GPCR kinase; KAADcyc, KAAD-cyclopamine; Nb, nanobody; PKA-C, PKA catalytic subunits; SMO, Smoothened. (PDF) [file pbio.3001191.s008.pdf]

# A

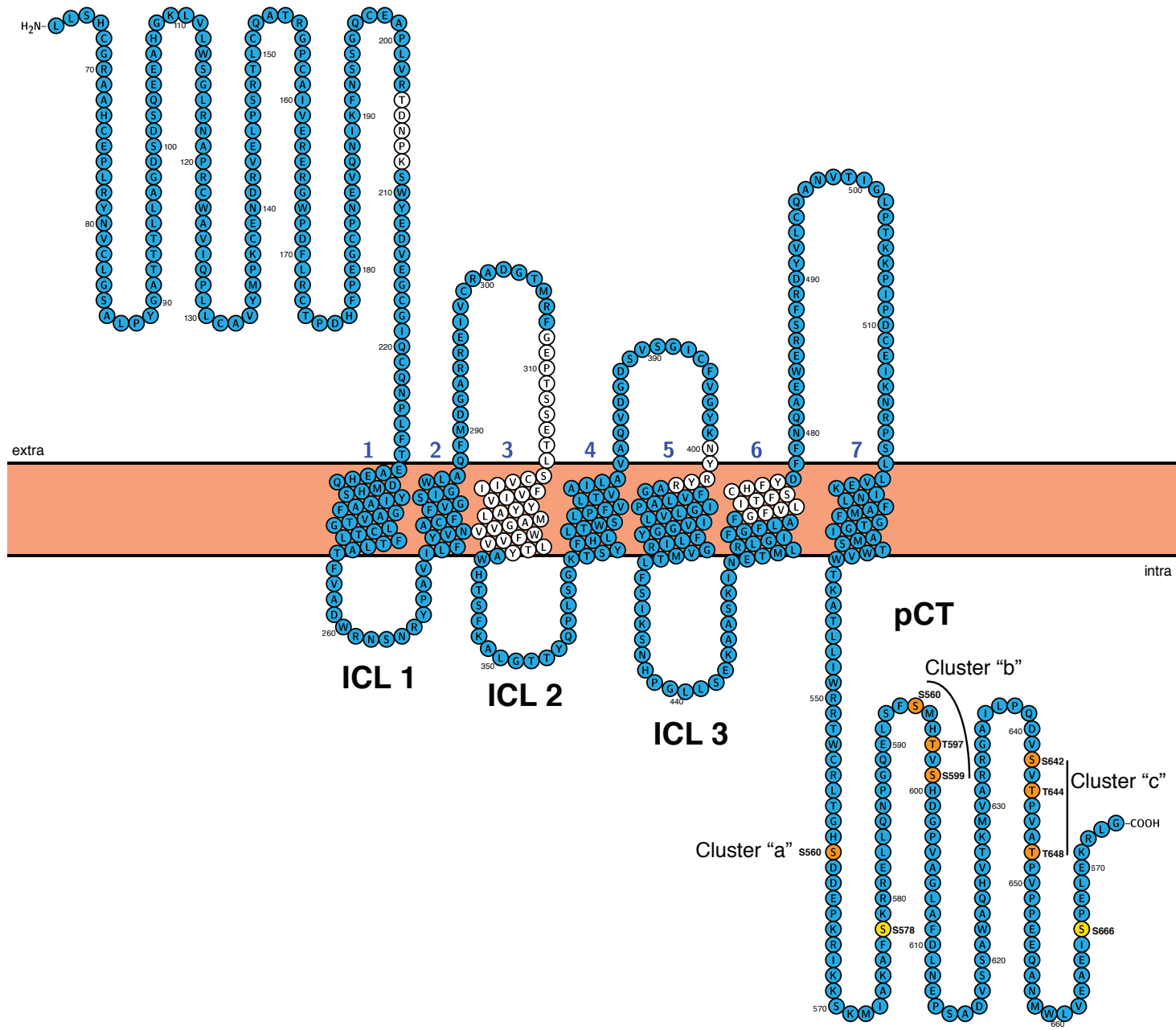

Supplement: S10 Fig — Related to Fig 6. Snake plot of the SMO674 construct used in this study. Residues covered by the global MS measurements are colored aqua, while residues that were not detected by MS are colored white. TM helices are numbered in navy. Regions potentially accessible to intracellular kinases (ICLs 1, 2, and 3 and the pCT domain) are labeled. Color coding of phosphorylation sites is identical to Figs 7 and S9. Residue numbering is with respect to untagged, full-length wild-type SMO. Signal sequence and N-terminal tags are omitted for clarity. ICL, intracellular loop; MS, mass spectrometry; pCT, proximal segment of the cytoplasmic tail; SMO, Smoothened. (PDF) [file pbio.3001191.s010.pdf]

**A**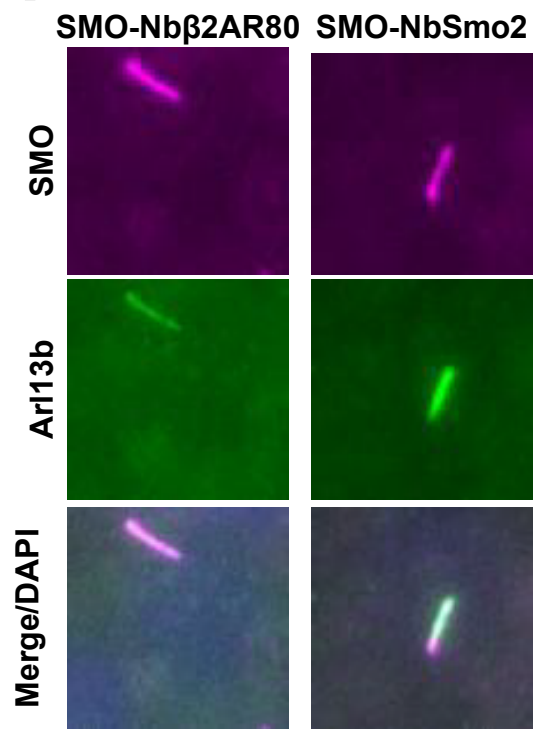**B**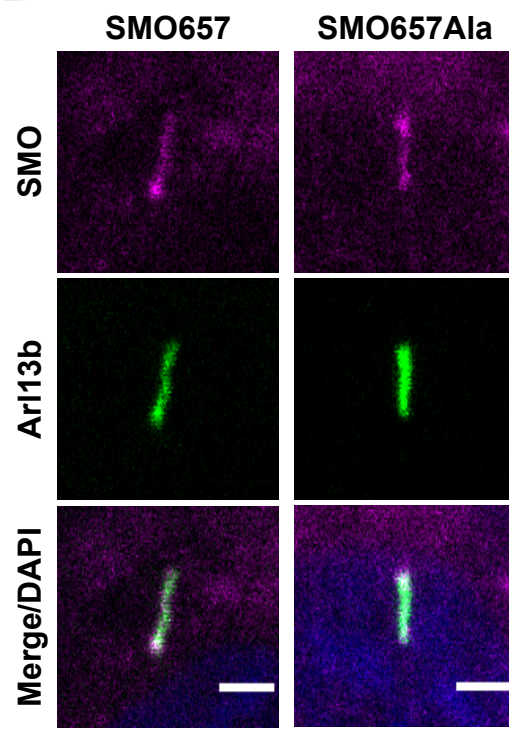**C**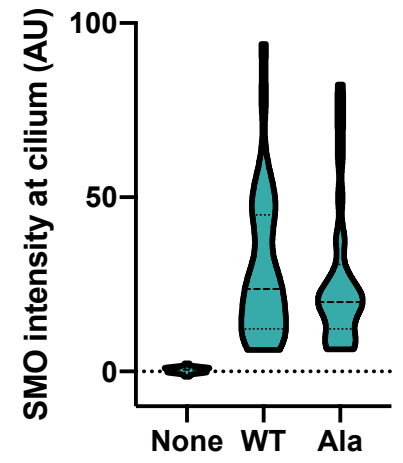

Supplement: S11 Fig — Related to Fig 7. Smo−/− fibroblasts expressing tdTomato-tagged (A) SMO-Nb fusions, or (B) the SMO Ala mutant were visualized via microscopy, following staining with antibodies to detect cilia (Arl13b), indicated in green. SMO is indicated in magenta. Representative images are shown, and the intensities of the ciliary SMO signals for wild-type SMO vs. the Ala mutant were quantified in (C) as described in “Methods” (n = 30 cilia per condition). DAPI counterstain (blue) marks the nucleus. Scale bar = 2.5 μm. The underlying data for this figure can be found under S7 Data. Nb, nanobody; SMO, Smoothened. (PDF) [file pbio.3001191.s011.pdf]
