# Supplementary material for: Smoothened transduces Hedgehog signals via activity-dependent sequestration of PKA catalytic subunits
Source: PLoS Biol. 2021 Apr 22;19(4):e3001191. doi: 10.1371/journal.pbio.3001191 (PMC8096101; doi:10.1371/journal.pbio.3001191)
Supplement: S9 Fig — Related to Fig 6. (A) Full alignment of SMO from Fig 6B, noting conservation of GRK2/3 among vertebrates, insects and basal metazoans. Interestingly, some GRK2/3 phosphorylation sites in mouse SMO are charged (D or E) residues in dSmo (i.e., S560), and vice versa (i.e., D601), consistent with the importance of negative charges at those positions. dSmo phosphorylation sites are from [80], in which sites verified as GRK-dependent are indicated in dark green, while sites that might be GRK-dependent but were not covered during their MS are indicated in light green. (B) Quantification of individual SMO and/or GRK2/3 activity-dependent phosphorylation sites and phosphorylation clusters from the targeted MS analysis in Fig 6B and 6C. Note that the S560 graph in this figure is identical to the cluster “a” graph in Fig 6C because cluster “a” comprises only a single phosphorylated residue. Unambiguous phosphorylation site localization on peptides with multiple candidate phosphorylation sites in close proximity can be challenging by MS [149] (see “Methods”). For these reasons, our quantification, and subsequent mutational analysis, focuses on clusters of residues rather than individual sites. In each graph, “/” means “or,” indicating that at least one of the sites listed here is phosphorylated, while “_” indicates that both of the sites are phosphorylated. n = 4 biological replicates per condition. The underlying data for this figure can be found under S6 Data. GRK, GPCR kinase; MS, mass spectrometry; SMO, Smoothened. (PDF) [file pbio.3001191.s009.pdf]

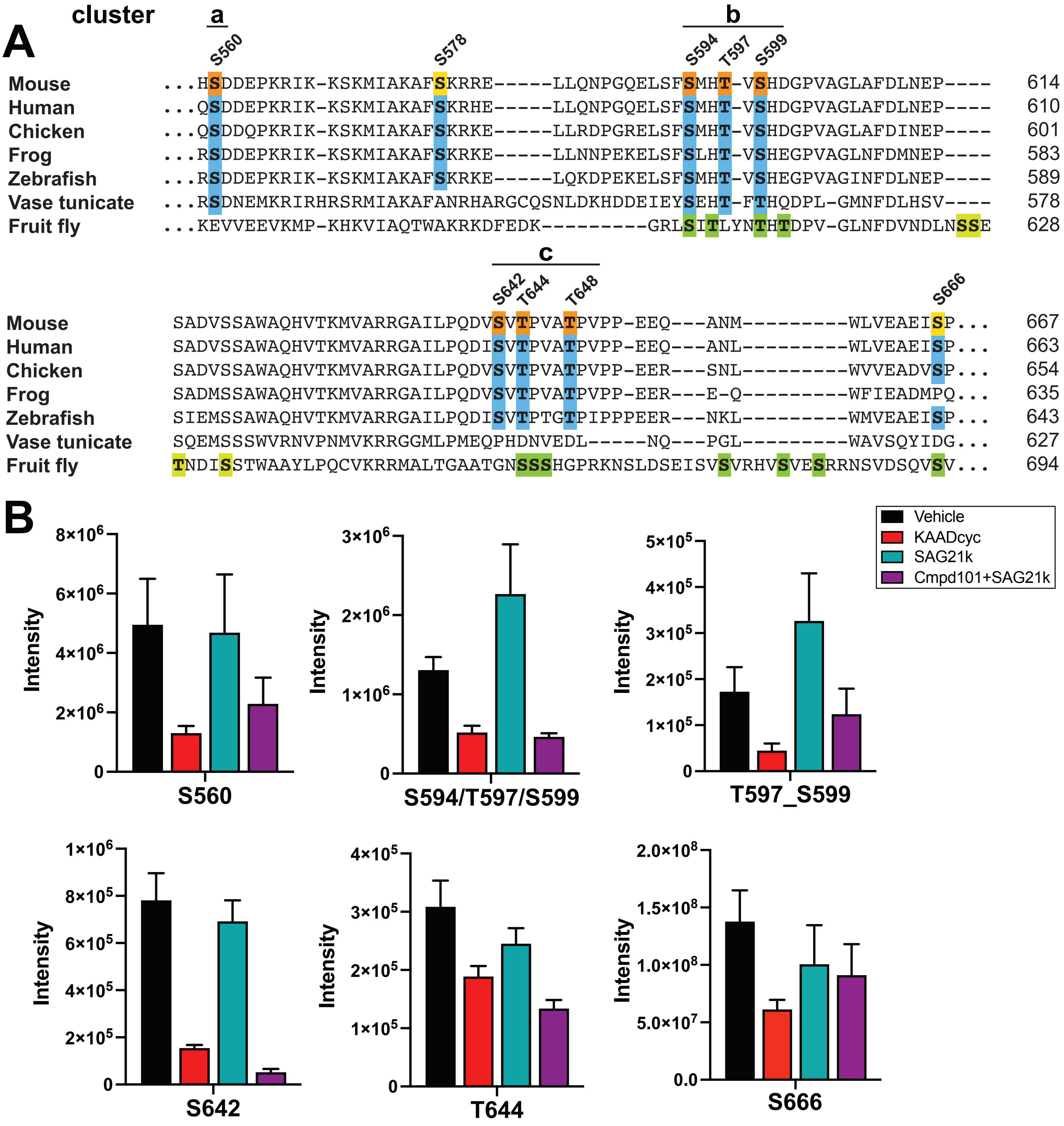

A
cluster a b c
S560 S578 S594 T597 S599
Mouse ...HSDDEPKRIK-KSKMIAKAFSKRRE-----LLQNPGQELSFSMHT-VSHDGPVAGLAFDLNEP---- 614
Human ...QSDDEPKRIK-KSKMIAKAFSKRHE-----LLQNPGQELSFSMHT-VSHDGPVAGLAFDLNEP---- 610
Chicken ...QSDDQPKRIK-KSKMIAKAFSKRKE-----LLRDPGRELSFSMHT-VSHDGPVAGLAFDINEP---- 601
Frog ...RSDDEPKRIK-KSKMIAKAFSKRKE-----LLNNPEKELSFSLHT-VSHEGPVAGLNFDMNEP---- 583
Zebrafish ...RSDDEPKRIK-KSKMIAKAFSKRKE-----LQKDPEKELSFSMHT-VSHEGPVAGINFDLNEP---- 589
Vase tunicate ...RSDNEMKRIRHRSRMIAKAFANRHARGCQSNLDKHDDEIEYSEHT-FTHQDPL-GMNFDLHSV---- 578
Fruit fly ...KEVVEEVKMP-KHKVIAQTWAKRKDFEDK---------GRLSITLYNTHTDPV-GLNFDVNDLNSSE 628
S642 T644 T648 S666
Mouse SADVSSAWAQHVTKMVARRGAILPQDVSVTPVATPVPP-EEQ---ANM---------WLVEAEISP... 667
Human SADVSSAWAQHVTKMVARRGAILPQDISVTPVATPVPP-EEQ---ANL---------WLVEAEISP... 663
Chicken SADVSSAWAQHVTKMVARRGAILPQDVSVTPVATPVPP-EER---SNL---------WVVEADVSP... 654
Frog SADMSSAWAQHVTKMVARRGAILPQDVSVTPVATPVPP-EER---E-Q---------WFIEADMPQ... 635
Zebrafish SIEMSSAWAQHVTKMVARRGAILPQDISVTPTGTPIPPPEER---NKL---------WMVEAEISP... 643
Vase tunicate SQEMSSSWVRNVPNMVKRRGGMLPMEQPHDNVEDL-----NQ---PGL---------WAVSQYIDG... 627
Fruit fly TNDISSTWAAYLPQCVKRRMALTGAATGNSSSHGPRKNSLDSEISVSVRHVSVESRRNSVDSQVSV... 694
B
Vehicle
KAADcyc
SAG21k
Cmpd101+SAG21k
Intensity
S560
S594/T597/S599
T597_S599
S642
T644
S666
