# Supplementary material for: Smoothened transduces Hedgehog signals via activity-dependent sequestration of PKA catalytic subunits
Source: PLoS Biol. 2021 Apr 22;19(4):e3001191. doi: 10.1371/journal.pbio.3001191 (PMC8096101; doi:10.1371/journal.pbio.3001191)
Supplement: S1 Table — Related to Figs 1–7 and S1–S11. (PDF) [file pbio.3001191.s012.pdf]

Supplemental Table 1: Tests of statistical significance for all figures

Figure.	Condition 1	Condition 2	Signifi-cant?	p-value (Student's t-test)
1B	PKA-C: Vehicle	PKA-C + SMO674: Vehicle	yes	0.005967
1B	PKA-C + SMO674: Vehicle	PKA-C + SMO674: KAADcyc	yes	0.010188
1B	PKA-C + M2AchR: Vehicle	PKA-C + M2AchR: Carbachol	no	0.353043
1D	PKA-C: Vehicle	PKA-C + SMO674: Vehicle	yes	0.000443
1D	PKA-C + SMO674: Vehicle	PKA-C + SMO674: KAADcyc	yes	0.035508
2C	SMO674 + PKA-C	SMO674 + Nbβ2AR80	yes	<0.0001
2C	SMO674 + NbSmo2	SMO674 + Nbβ2AR80	yes	<0.0001
2C	SMO674 + PKA-C	SMO566 + PKA-C	yes	0.0004
2E	SMO + PKA-C	SMO + Nbβ2AR80	yes	<0.0001
2E	SMO + NbSmo2	SMO + Nbβ2AR80	yes	<0.0001
3A	SMO + PKA-C	SMO + βarrestin1	yes	<0.000001
3B	657: PKA-C	Δ561-657: PKA-C	yes	0.00007
3B	566: βarrestin1	566: PKA-C	no	0.072522
3C	SMO + PKA-C	SMO + PKA-R	yes	0.018434
3D	Parental: PKA-C	Parental: PKA-R	no	0.92467
3D	HiBiT Knock-in: PKA-C-YFP	HiBiT Knock-in: PKA-R-YFP	yes	0.001717
3D	Parental: PKA-C	HiBiT Knock-in: PKA-C	yes	0.012103
4B	PKA-C	PKA-R	yes	0.00012
4C	PKA-C-YFP: Vehicle	PKA-C(no tag) + PKA-R-YFP: Vehicle	yes	0.000085
4C	PKA-C-YFP + PKA-R (no tag): Vehicle	PKA-C-YFP + PKA-R (no tag): fsk/IBMX	yes	0.010792
4E	WT PKA-C	H87Q / W196R	no	0.682648
4E	WT PKA-C	L206R	yes	0.027339
4E	WT PKA-C	K73H	yes	0.007473
4F	WT PKA-C	Δ1-24	yes	0.000368
4F	WT PKA-C	Δ1-39	yes	0.000095
5A	NbSmo2: SAG21k	NbSmo2: KAADcyc	yes	0.000002
5A	PKA-C: SAG21k	PKA-C: KAADcyc	yes	0.000072

5C	SAG21k	KAADcyc	yes	<0.0001
5C	SAG21k	SAG21k + Cmpd101	yes	0.0372
5C	Vehicle	KAADcyc	yes	<0.0001
5D	NbSmo2: SAG21k	NbSmo2: SAG21k + Cmpd101	no	0.896283
5D	PKA-C: SAG21k	PKA-C: SAG21k + Cmpd101	yes	0.000213
5E	PKA-C + SMO674: Vehicle	PKA-C + SMO674: Cmpd101	yes	0.012301
6C	Cluster a, Vehicle	Cluster a, KAADcyc	yes	2.23E-04
6C	Cluster a, SAG21k	Cluster a, Cmpd101/SAG21k	yes	1.42E-02
6C	Cluster b, Vehicle	Cluster b, KAADcyc	yes	4.68E-05
6C	Cluster b, SAG21k	Cluster b, Cmpd101/SAG21k	yes	8.93E-07
6C	Cluster c, Vehicle	Cluster c, KAADcyc	yes	4.68E-05
6C	Cluster c, SAG21k	Cluster c, Cmpd101/SAG21k	yes	1.91E-06
6C	Total SMO, Vehicle	Total SMO, KAADcyc	no	0.636116
6C	Total SMO, SAG21k	Total SMO, Cmpd101/SAG21k	no	0.272159
6D	SMO657: PKA-C	SMO657Ala: PKA-C	yes	0.00249
7A	SMOWT: ShhN	SMOΔ570-581: ShhN	yes	0.000252
7B	SMO657: PKA-C	SMO657(Δ570-581): PKA-C	yes	<0.000001
7D	SMO-Nbβ2AR80: Vehicle	SMO-Nbβ2AR80: ShhN	yes	0.000216
7D	SMO-NbSmo2: Vehicle	SMO-NbSmo2: ShhN	no	0.453546
7F	SMO657WT: ShhN	SMO657Ala: ShhN	yes	0.000198
S1C	SMO657WT: ShhN	SMOΔ561-657: ShhN	yes	.000007
S1E	SMO: Vehicle	SMO + PTCH1: Vehicle	yes	0.000009
S1E	SMO + PTCH1: Vehicle	SMO + PTCH1: ShhN	yes	0.002962
S1F	Wild Type: PKA-C: Vehicle	Wild Type: PKA-C + SMO674: Vehicle	yes	0.026726
S1F	Wild Type: PKA-C + SMO674: Vehicle	Wild Type: PKA-C + SMO674: KAADcyc	no	0.080703
S1F	Gα-null: PKA-C: Vehicle	Gα-null: PKA-C + SMO674: Vehicle	yes	0.000236
S1F	Gα-null: PKA-C + SMO674: Vehicle	Gα-null: PKA-C + SMO674: KAADcyc	yes	0.007104
S1G	Wild Type: M2AchR: fsk	wild-type cells: M2AchR: fsk + Carbachol	yes	0.002942
S1G	Gα-null: M2AchR: fsk	Gα-null: M2AchR: fsk + Carbachol	no	0.75318

S4A	SMO674: PKA-C	SMO566: PKA-C	yes	0.000116
S4B	full-length: βarrestin1	full-length: PKA-C	yes	<0.000001
S4B	657: βarrestin1	657: PKA-C	yes	0.000005
S4B	614: βarrestin1	614: PKA-C	yes	0.000002
S4B	574: βarrestin1	574: PKA-C	yes	0.002667
S4B	566: βarrestin1	566: PKA-C	no	0.445983
S4C	SMO657: Highest YFPext./nanoluc	PTCH1: Highest YFPext./nanoluc	yes	<0.000001
S4C	SMO657: Highest YFPext./nanoluc	DRD2: Highest YFPext./nanoluc	yes	0.000009
S4F	SMO657: βarrestin1	SMO657: NbSmo2	yes	<0.000001
S4F	SMO657: βarrestin1	SMO657: PKA-C	yes	0.000004
S5A	SMO	SMO566	yes	0.001191
S5A	SMO	PTCH1	yes	0.000705
S5A	SMO	DRD2	yes	0.000487
S5E	555-674: βarrestin1	555-674: PKA-C	yes	0.000015
S7A	PKA-C: Highest YFPext./nanoluc	PKA-R: Highest YFPext./nanoluc	yes	0.000622
S8C	NbSmo2: KAADcyc	NbSmo2: SAG21k	yes	<0.0001
S8C	Nbβ2AR80: KAADcyc	Nbβ2AR80: SAG21k	no	0.1977
S8C	NbSmo2: KAADcyc	Nbβ2AR80: KAADcyc	no	0.0747
S8F	Parental: SMO	∆GRK:SMO	yes	0.001777
S8F	∆GRK: SMO	∆GRK: SMO + GRK2	yes	0.005525
S9B	S560, Vehicle	S560, KAADcyc	yes	2.93E-04
S9B	S560, SAG21k	S560, Cmpd101/SAG21k	yes	1.50E-02
S9B	S594 / T597 / S599, Vehicle	S594 / T597 / S599, KAADcyc	yes	6.71E-05
S9B	S594 / T597 / S599, SAG21k	S594 / T597 / S599, Cmpd101/SAG21k	yes	3.16E-07
S9B	S642, Vehicle	S642, KAADcyc	yes	1.12E-07
S9B	S642, SAG21k	S642, Cmpd101/SAG21k	yes	4.53E-10
S9B	S666, Vehicle	S666, KAADcyc	yes	7.08E-04
S9B	S666, SAG21k	S666, Cmpd101/SAG21k	no	5.89E-01
S9B	T597, Vehicle	T597, KAADcyc	yes	8.35E-04
S9B	T597_S599, SAG21k	T597_S599, Cmpd101/SAG21k	yes	7.95E-03

S9B	T644, Vehicle	T644, KAADcyc	yes	1.21E-04
S9B	T644, SAG21k	T644, Cmpd101/SAG21k	yes	1.41E-05
S9B	Total SMO (sites), Vehicle	Total SMO (sites), KAADcyc	no	6.36E-01
S9B	Total SMO (sites), SAG21k	Total SMO (sites), Cmpd101/SAG21k	no	3.18E-01
S11C	SMO657-WT	SMO657-Ala	no	0.5279
