# Supplementary material for: Smoothened transduces Hedgehog signals via activity-dependent sequestration of PKA catalytic subunits
Source: PLoS Biol. 2021 Apr 22;19(4):e3001191. doi: 10.1371/journal.pbio.3001191 (PMC8096101; doi:10.1371/journal.pbio.3001191)
Supplement: S2 Table — Related to Figs 6 and S9. MS, mass spectrometry; SMO, Smoothened. (PDF) [file pbio.3001191.s013.pdf]

Supplemental Table 2. Initial identification and quantification of SMO phosphorylation sites via untargeted mass spectrometry. "Tryp/Chy" denotes the double digestion of the protein with trypsin and chymotrypsin, "Chy" indicates digestion with chymotrypsin alone.

Phosphosite	Cluster	Digestion	Comparison	log2FC	Std Err
S560	a	Tryp/Chy	Vehicle-KAADcyc	3.949	0.934
S560	a	Tryp/Chy	SAG21k-101/SAG21k	3.322	0.934
S594	b	Tryp/Chy	Vehicle-KAADcyc	0.565	1.730
S594	b	Tryp/Chy	SAG21k-101/SAG21k	1.260	1.413
T597	b	Tryp/Chy	Vehicle-KAADcyc	2.145	1.955
T597	b	Tryp/Chy	SAG21k-101/SAG21k	1.157	1.597
S599	b	Tryp/Chy	Vehicle-KAADcyc	1.931	1.519
S599	b	Tryp/Chy	SAG21k-101/SAG21k	0.587	1.386
S642	c	Tryp/Chy	Vehicle-KAADcyc	1.695	1.834
S642	c	Tryp/Chy	SAG21k-101/SAG21k	Inf	NA
T644	c	Tryp/Chy	Vehicle-KAADcyc	0.602	1.110
T644	c	Tryp/Chy	SAG21k-101/SAG21k	0.268	1.282
T648	c	Tryp/Chy	Vehicle-KAADcyc	Inf	NA
T648	c	Tryp/Chy	SAG21k-101/SAG21k	Inf	NA
S666		Tryp/Chy	Vehicle-KAADcyc	-0.178	1.504
S666		Tryp/Chy	SAG21k-101/SAG21k	-0.820	1.504
total SMO		Tryp/Chy	Vehicle-KAADcyc	0.130	0.259
total SMO		Tryp/Chy	SAG21k-101/SAG21k	-0.146	0.259
S578		Chy	Vehicle-KAADcyc	-0.368	0.996
S578		Chy	SAG21k-101/SAG21k	0.336	0.891
S594	b	Chy	Vehicle-KAADcyc	0.920	0.587
S594	b	Chy	SAG21k-101/SAG21k	1.727	0.587
T597	b	Chy	Vehicle-KAADcyc	negInf	NA
T597	b	Chy	SAG21k-101/SAG21k	Inf	NA
S599	b	Chy	Vehicle-KAADcyc	3.326	1.523
S599	b	Chy	SAG21k-101/SAG21k	0.869	1.391
S666		Chy	Vehicle-KAADcyc	-0.299	1.270
S666		Chy	SAG21k-101/SAG21k	0.386	1.270
total SMO		Chy	Vehicle-KAADcyc	0.165	0.348
total SMO		Chy	SAG21k-101/SAG21k	0.251	0.348
