# Supplementary material for: Smoothened transduces Hedgehog signals via activity-dependent sequestration of PKA catalytic subunits
Source: PLoS Biol. 2021 Apr 22;19(4):e3001191. doi: 10.1371/journal.pbio.3001191 (PMC8096101; doi:10.1371/journal.pbio.3001191)

# Fig. 1C

Transfected DNA
1=GFP
2=PKA-C
3=PKA-C + SMO

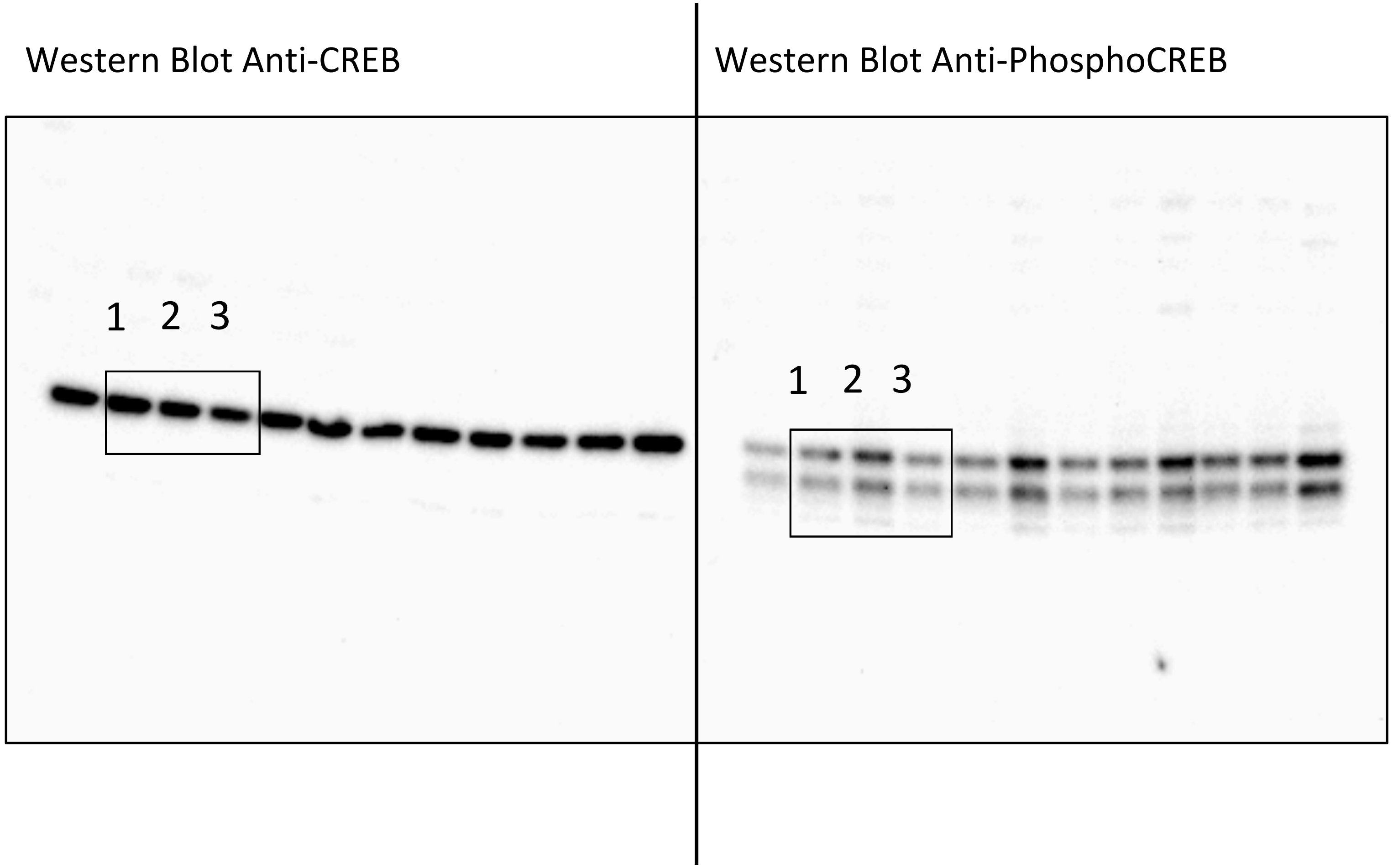

**Fig. S1D**

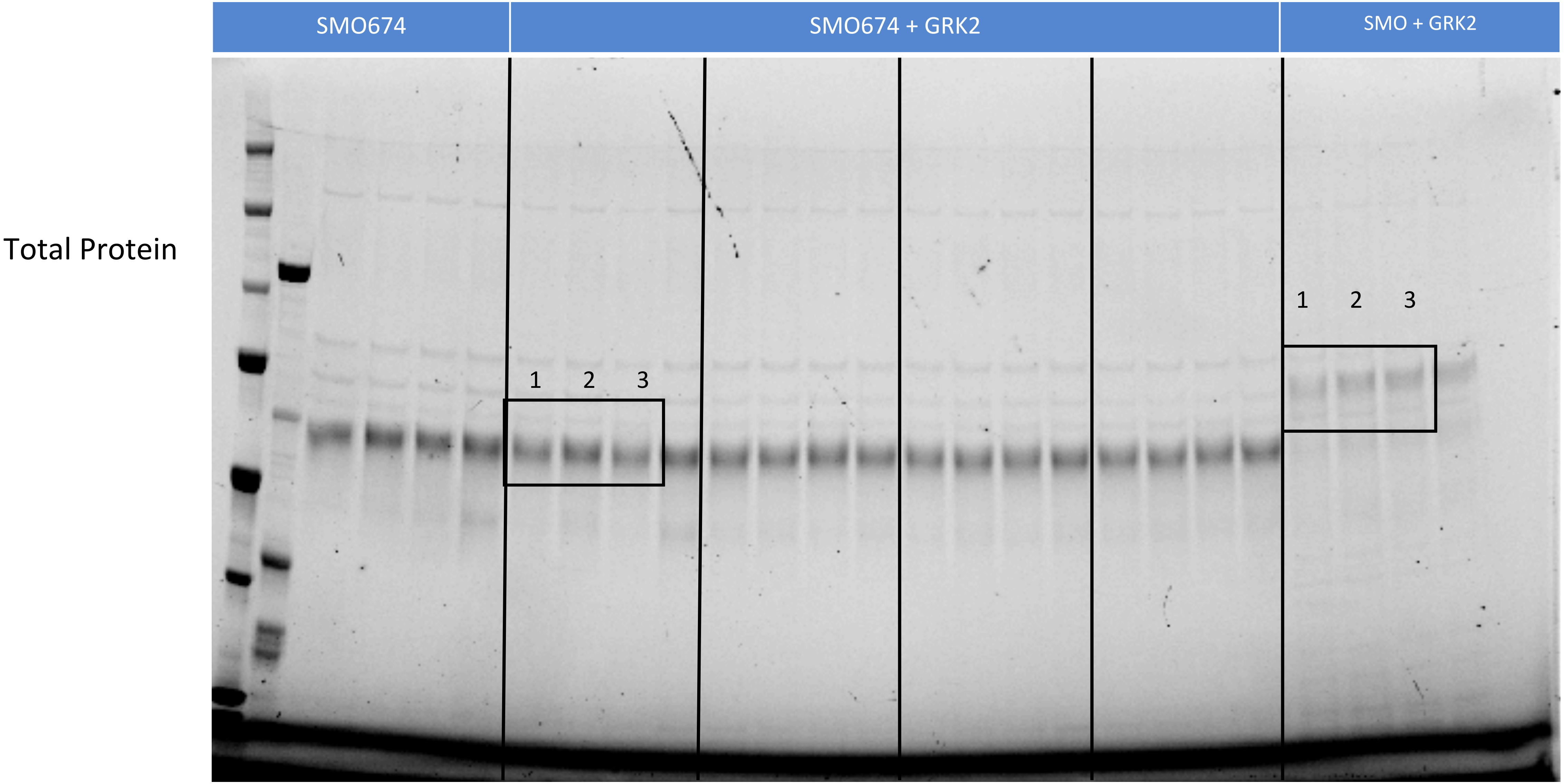


1= vehicle; 2=KAAD; 3=SAG21K

# Fig. 3C

# LiCor (WB) in IMCD3

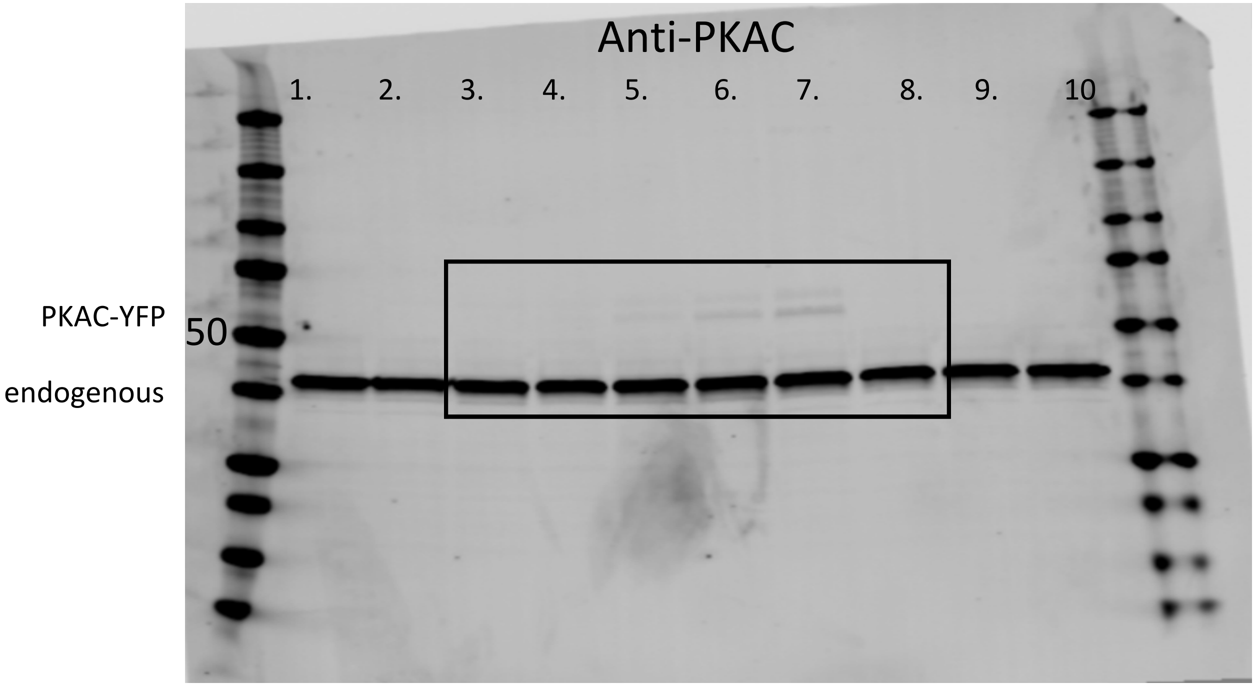

# Fig. 3C

# LiCor (WB) in IMCD3

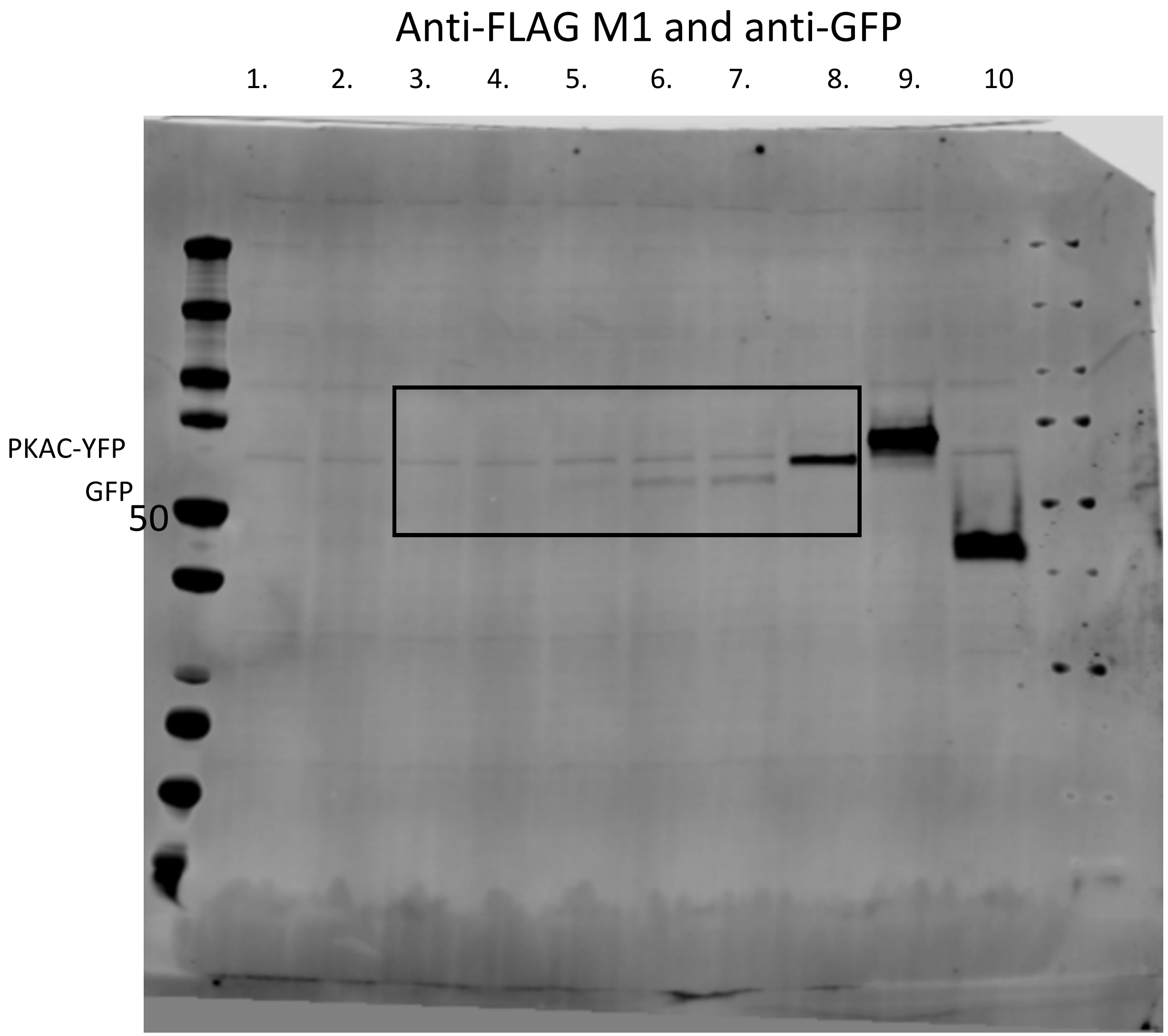

**Fig. 3E**

1= 0mM DSP
2=0.125mM DSP
3=0.25mM DSP
4=0.5mM DSP
5=1mM DSP
6=2mM DSP

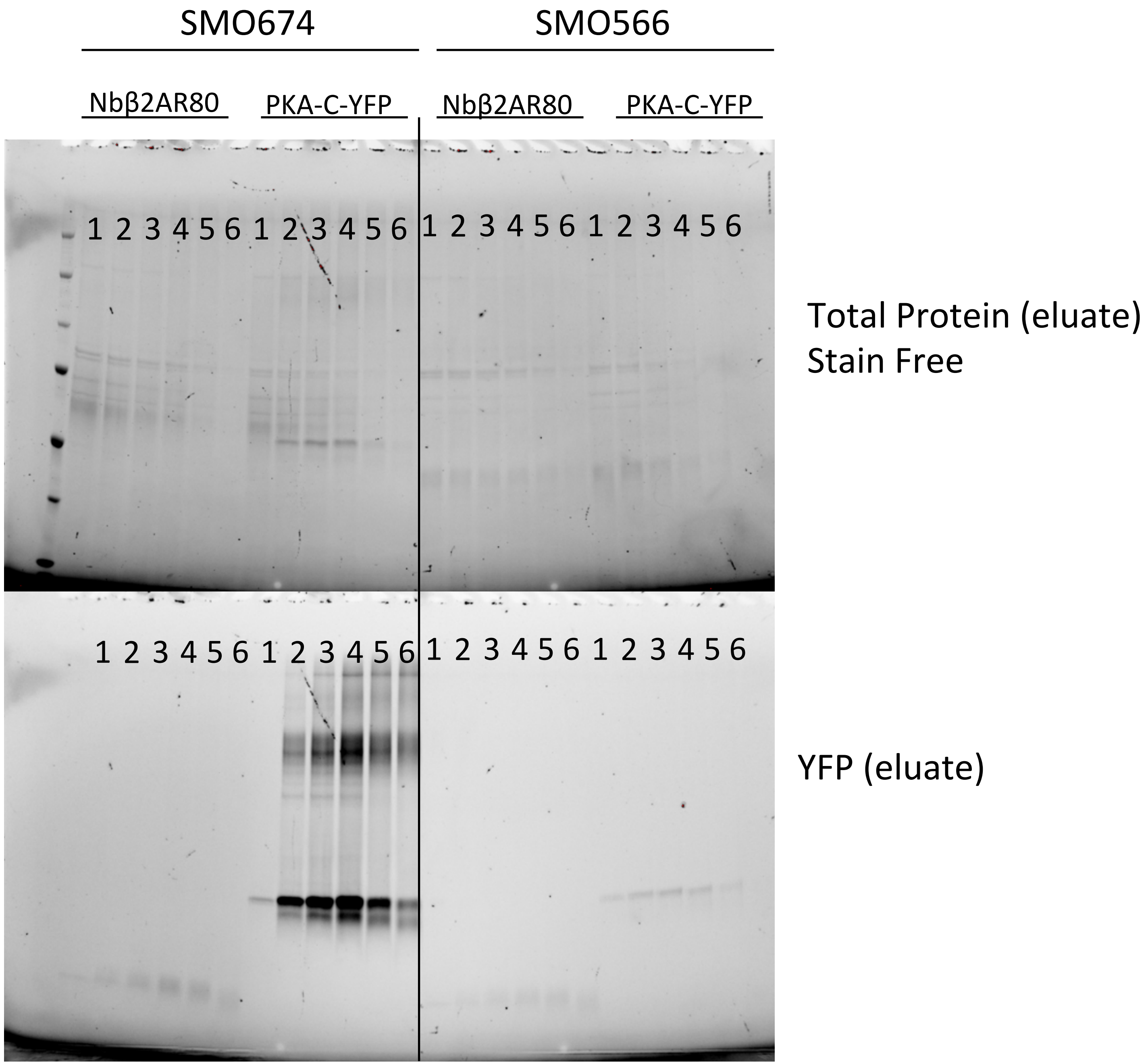

**Fig. S5C**

1st lane = ladder
2nd lane = IMCD3 parental
3rd lane = IMCD3 SMO HiBiT knock-in (clone 45)

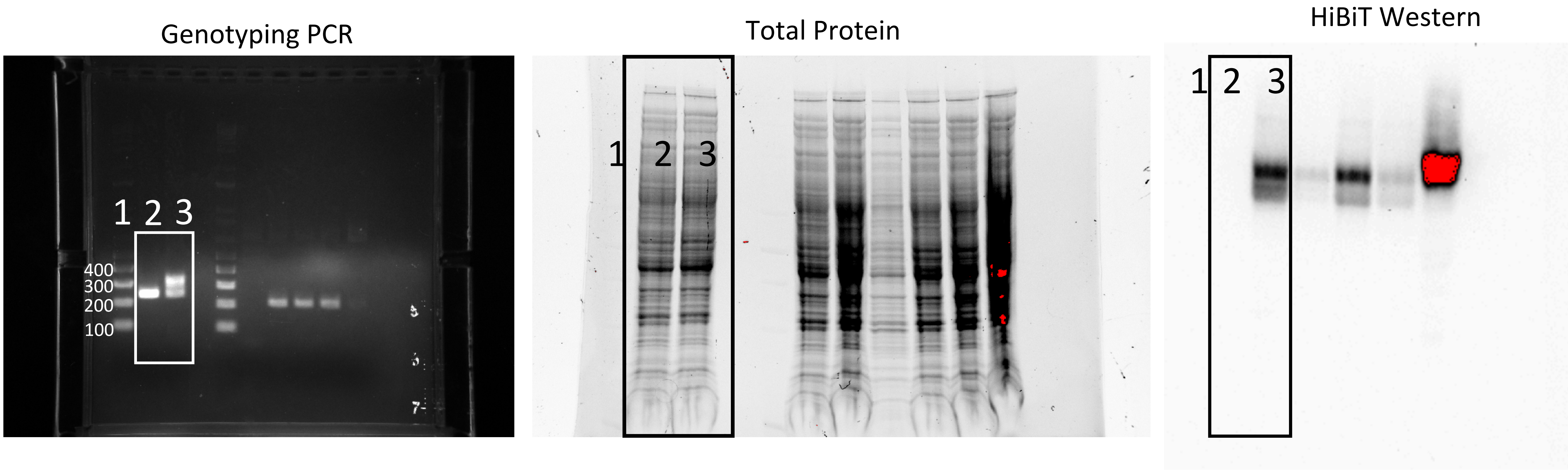

# Fig. S6C

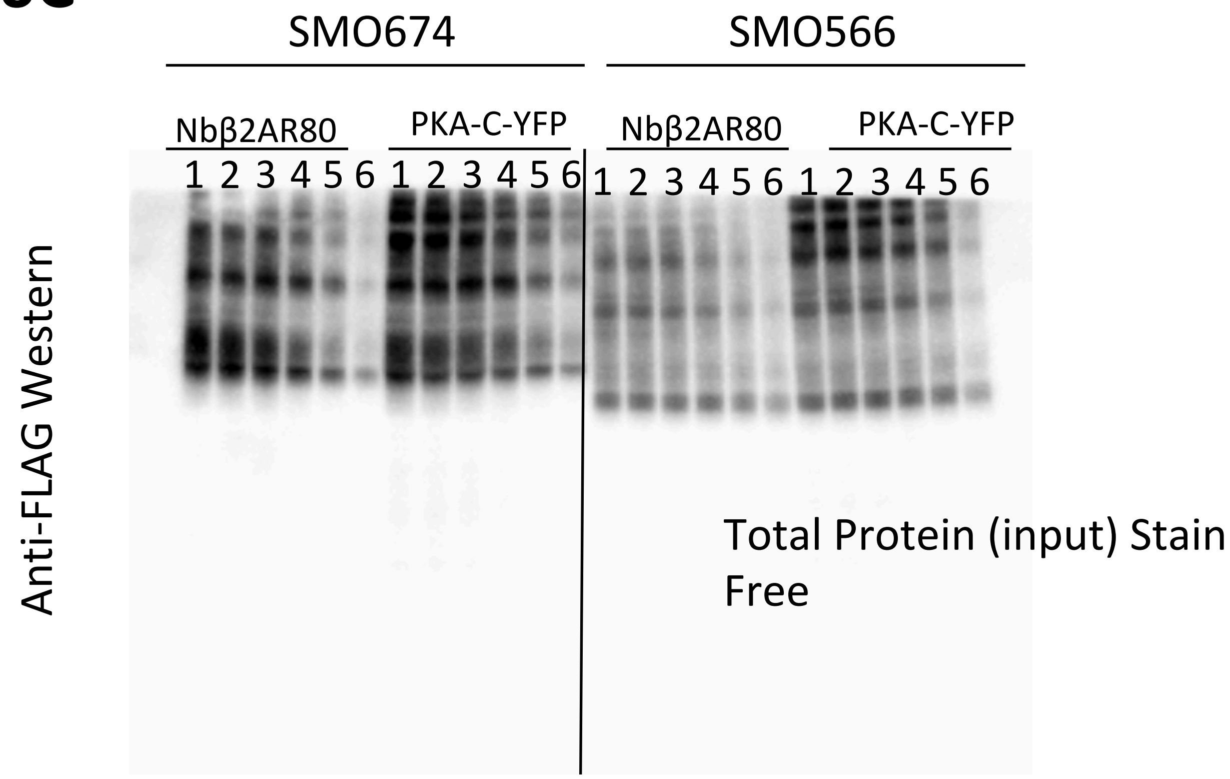


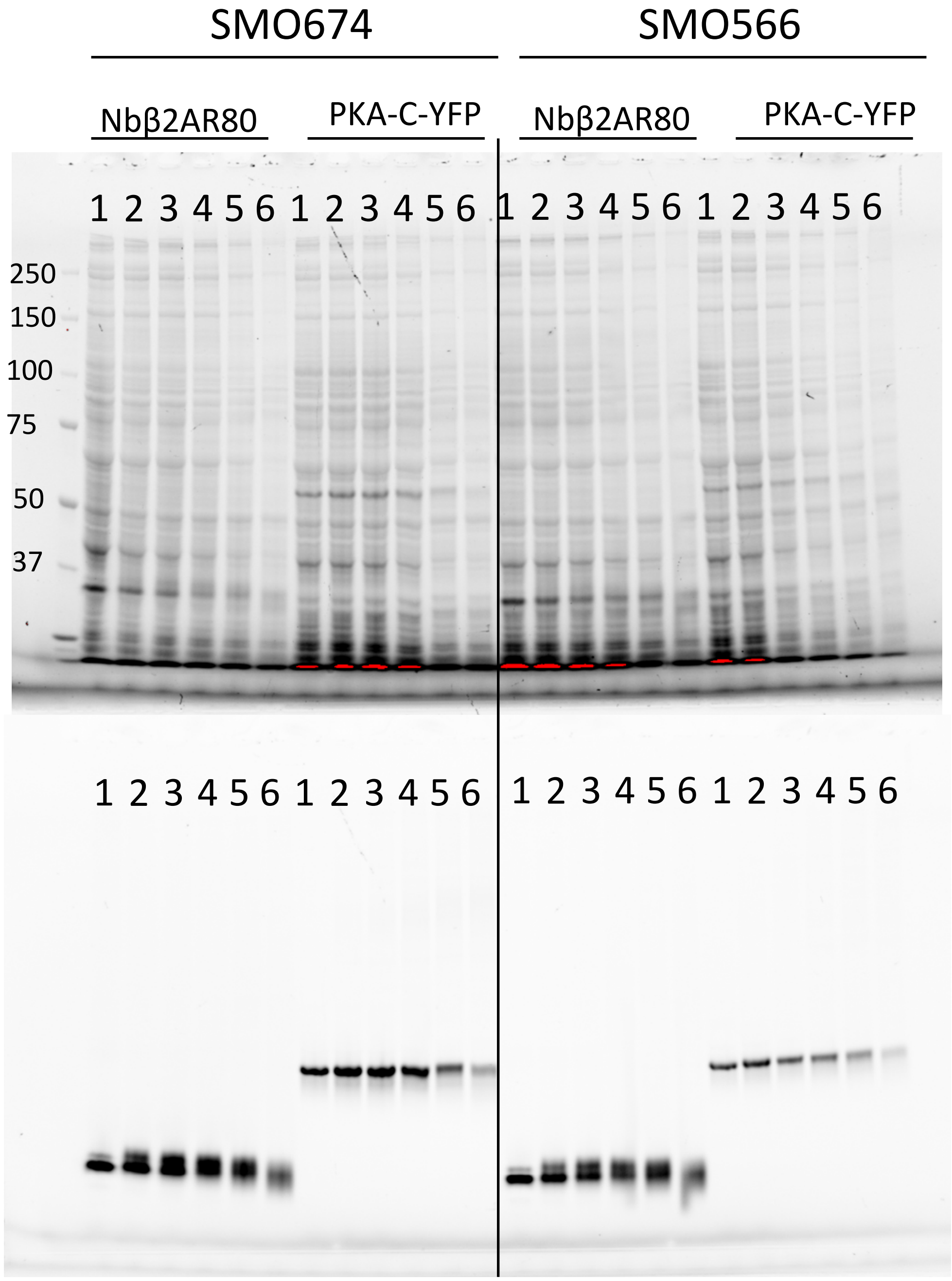


YFP (input)

1= 0mM DSP
2=0.125mM DSP
3=0.25mM DSP
4=0.5mM DSP
5=1mM DSP
6=2mM DSP

# Fig. S7B

Western Blot Anti-PKA-R

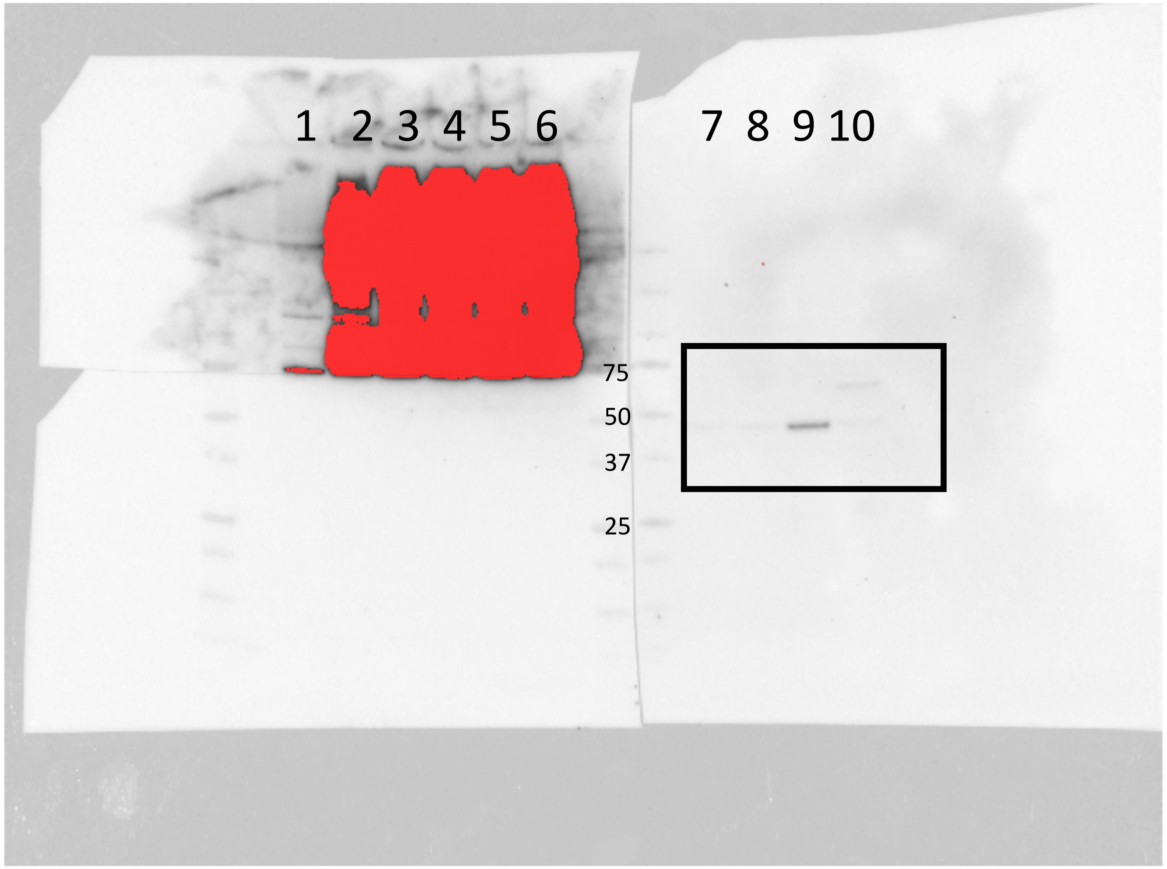

# Fig. S8D

Western Blot

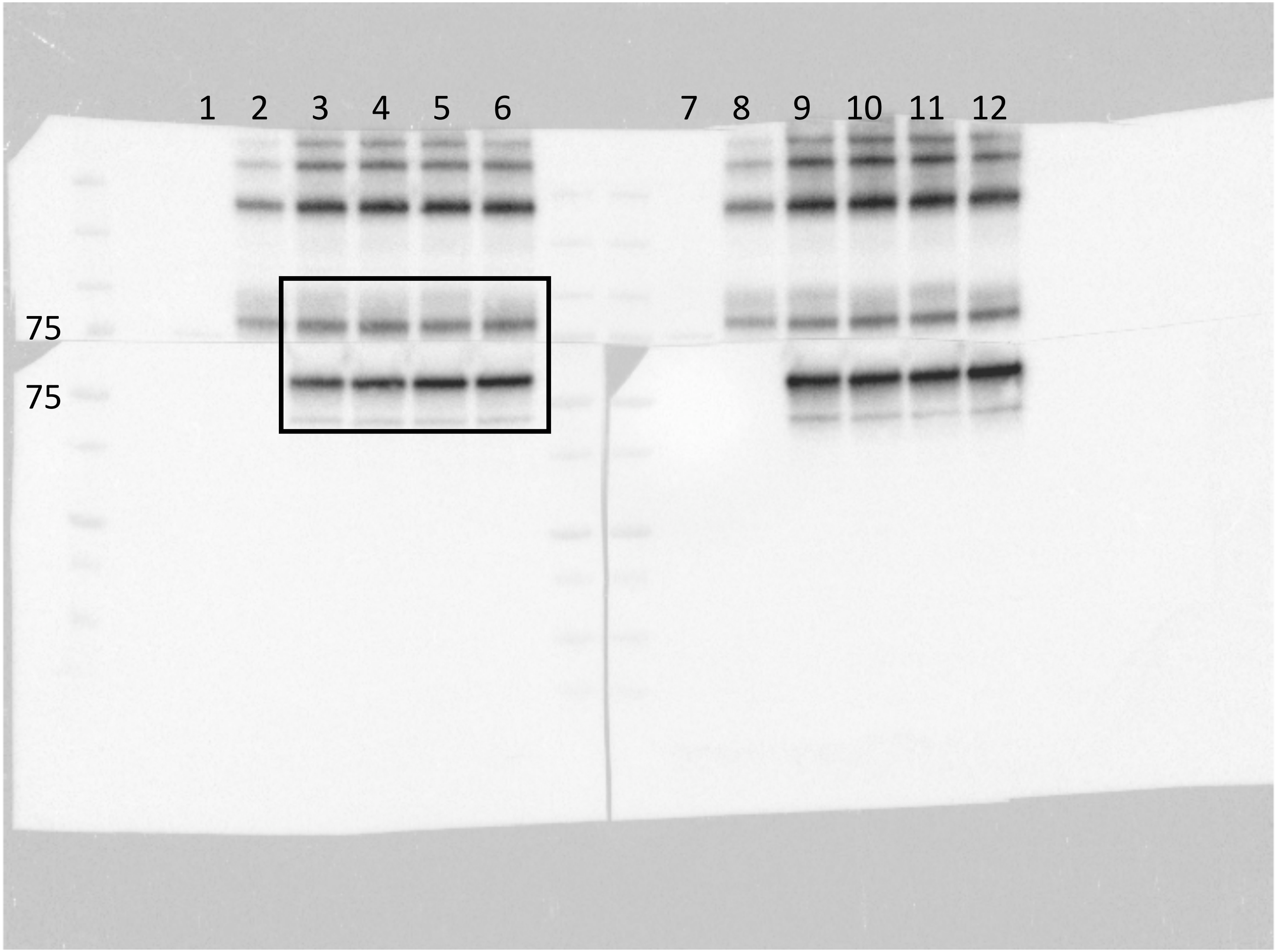

**Fig. 6A**

# ProQ Diamond

Total Protein

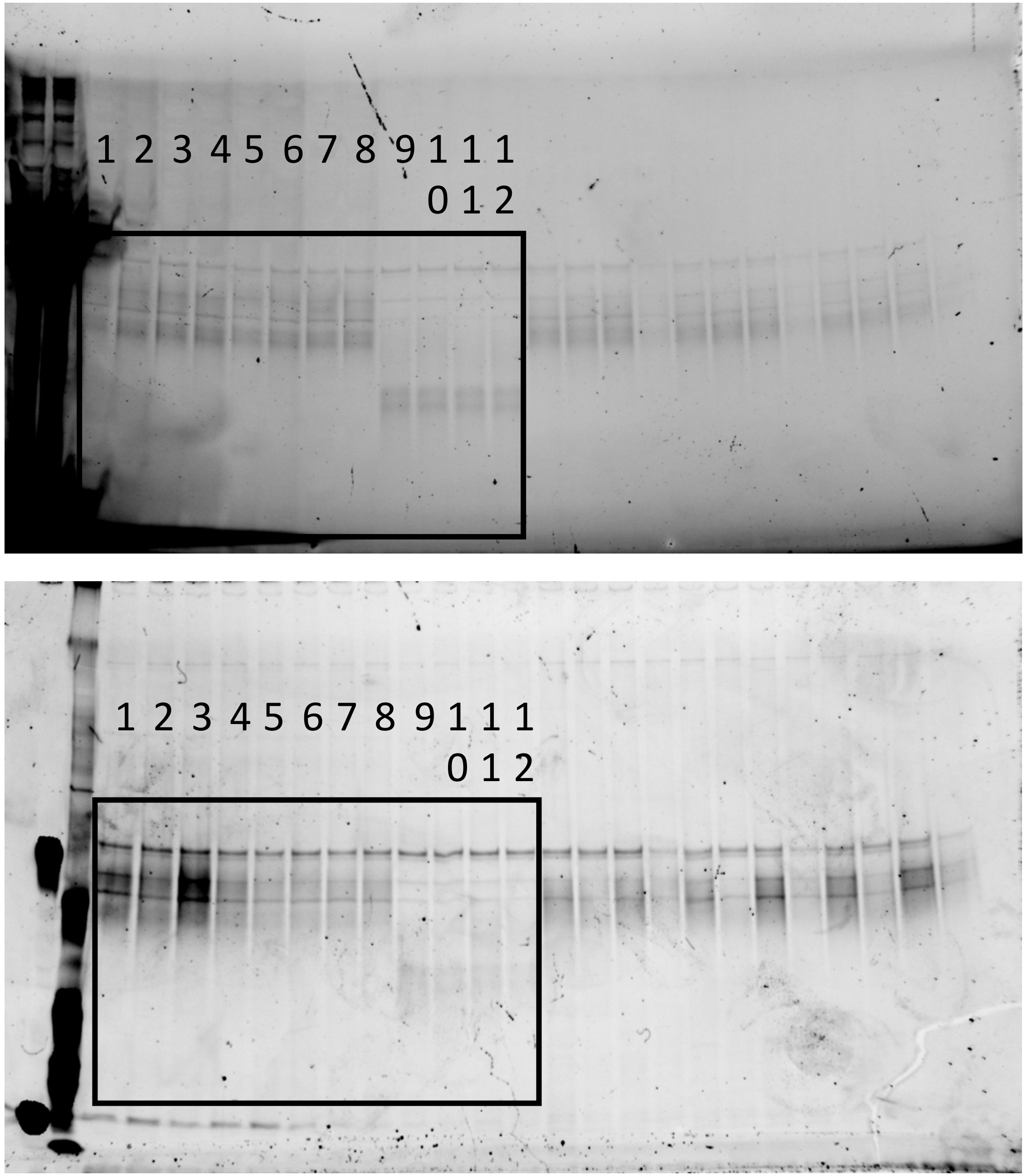


Phospho Protein

**Fig. 7C**

1=SMO674
2=SMO566
3=SMO-NbSmo2
4=SMO-Nbβ2AR80

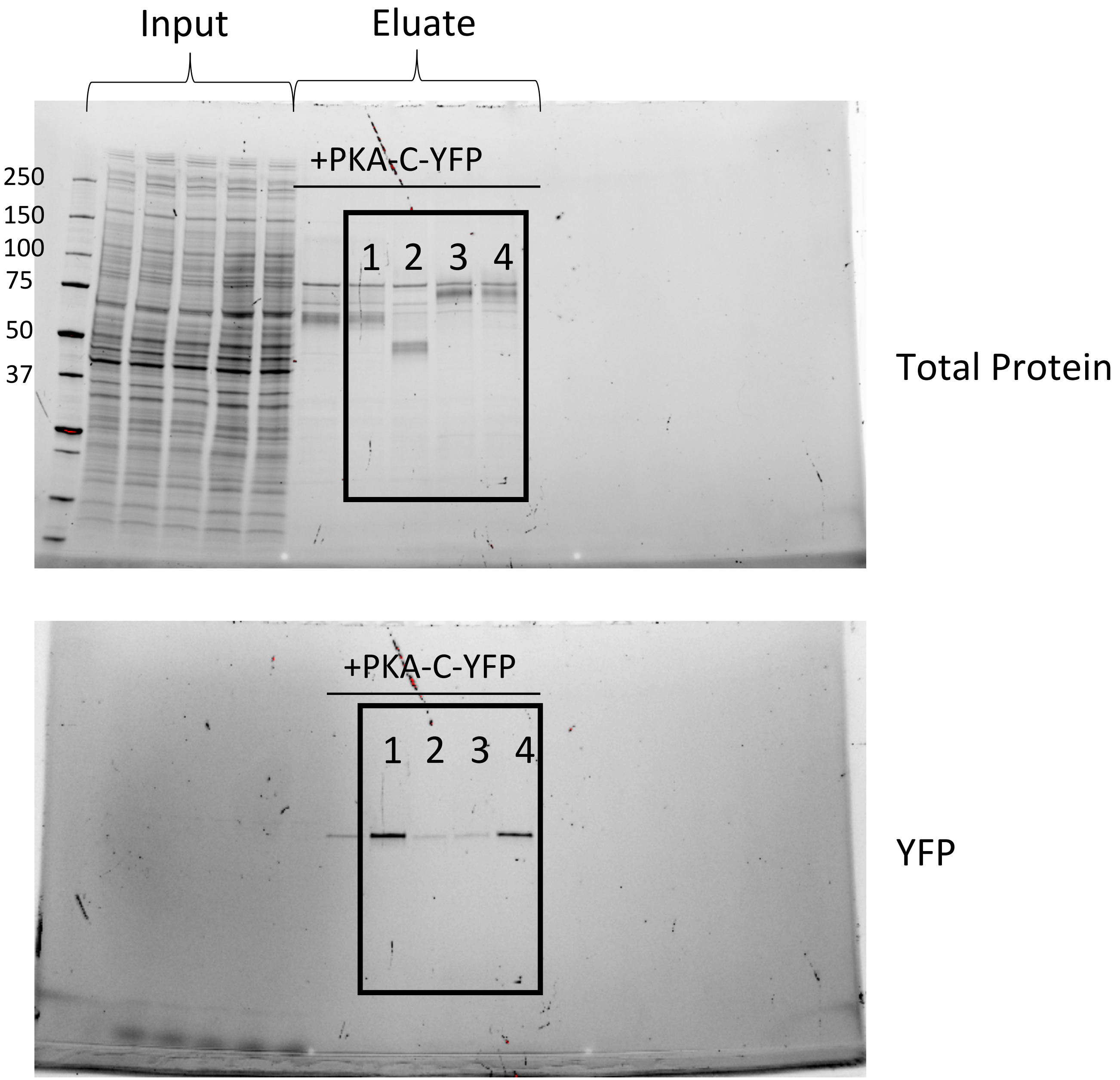

Supplement: S8 Data — (PDF) [file pbio.3001191.s021.pdf]
